# Supplementary material for: mHealth: providing a mindfulness app for women with chronic pelvic pain in gynaecology outpatient clinics: qualitative data analysis of user experience and lessons learnt
Source: BMJ Open. 2020 Mar 12;10(3):e030711. doi: 10.1136/bmjopen-2019-030711 (PMC7069307; doi:10.1136/bmjopen-2019-030711)
Supplement: Supplementary data [file bmjopen-2019-030711supp001.pdf]

APPENDIX 1

Feature	Positive aspects	Negative aspects	Solutions
**Voice**	Liked by some participants, considered soothing and even spiritual	The voice was not relaxing. One had to listen carefully which meant you could not switch off; otherwise you could not hear instruction clearly during meditation.<br><br>It was also too repetitive, after 10 times it felt like a chore and not something to look forward to.	Choice of different voices<br><br>participants should be told it takes time to get into the rhythm of the instructions, but you can get used to it
**General interface aesthetics**	colours really fresh, interface not too busy, with pleasing layout and aesthetics	one participant would prefer different, bright, colours such as purple	None needed
**Graphics**		The pictures were often not appropriate as they focused on the problem (e.g. tooth pain logo) rather than something positive or soothing. Unanticipated problems could arise according to one participant; she found it hard to lose weight and felt the food icons a) did not represent success and b) reminded her of eating.<br><br>One participant found the pictures hard to see.	Psychological theory supports the view that positive images would be advisable

**Animations**	liked	poorly chosen	None needed
**Progress**	One participant liked the way the app helped progress through the stages.<br>Another participant, with cyclical pain and hence cyclical use, did not aim to progress but simply chose to use the baseline video each time.	Three participants were frustrated and even 'angry' and 'patronised' at having to do the same (basic) meditation several times before moving up a stage.<br>There was no indication of goals, duration of each level or gamification rewards.	Goal setting is recommended by psychological theory and could be as simple as using "day 1 of 30" rather than simply Day 1.<br>Changing emojis could show progress, for example from a sad face to a smiley face to a heart; the inbuilt progress function was not felt sufficiently motivating.
**Introduction**		Experienced app users suggested the introduction could be improved for initiates	overview of the entire app and its levels suggested for the start.<br>One participant had done mindfulness before but thought an introduction to mindfulness might be helpful for others.
**Enjoyment**	considered fun by some participants.	One said it was not very 'interesting'.	Include examples of a patient's day with the app to cater for a greater variety of participants<br>It would be good to have other features as drop down options that were fun and

			did not involve meditation.

**Session intensity**		Would be good to choose the duration of the meditation.	15 or 20 mins would be better than 10, which is not enough time shut off and meditate deeply.<br><br>The option to control this was important as even 10 minutes was a difficult commitment for some busy participants (1074, 1075, 1078) or those battling with chronic fatigue (1074)
**Pain modules**		it can be hard to focus on something like using the app when you are in pain.	None needed

Comments on app acceptability and usability made by n=13 patients. Two patients walked through the app with the researcher
