# Supplementary material for: mHealth: providing a mindfulness app for women with chronic pelvic pain in gynaecology outpatient clinics: qualitative data analysis of user experience and lessons learnt
Source: BMJ Open. 2020 Mar 12;10(3):e030711. doi: 10.1136/bmjopen-2019-030711 (PMC7069307; doi:10.1136/bmjopen-2019-030711)
Supplement: Author's manuscript [file bmjopen-2019-030711.draft_revisions.pdf]

BMJ Open

BMJ Open is committed to open peer review. As part of this commitment we make the peer review history of every article we publish publicly available.

When an article is published we post the peer reviewers' comments and the authors' responses online. We also post the versions of the paper that were used during peer review. These are the versions that the peer review comments apply to.

The versions of the paper that follow are the versions that were submitted during the peer review process. They are not the versions of record or the final published versions. They should not be cited or distributed as the published version of this manuscript.

BMJ Open is an open access journal and the full, final, typeset and author-corrected version of record of the manuscript is available on our site with no access controls, subscription charges or pay-per-view fees (http://bmjopen.bmj.com).

If you have any questions on BMJ Open's open peer review process please email info.bmjopen@bmj.com

BMJ Open

Mhealth – Using a Mindfulness App for women with chronic pelvic pain: Qualitative data analysis of user experience and lessons learnt

Journal:	*BMJ Open*
Manuscript ID	bmjopen-2019-030711
Article Type:	Research
Date Submitted by the Author:	15-Apr-2019

Complete List of Authors:	Ball, ELizabeth; Queen Mary University of London - Whitechapel Campus, Yvonne Carter Building<br>Newton, Sian<br>Rohricht, Frank ; East London NHS Foundation Trust<br>Steed, Liz; Barts and The London School of Medicine and Dentistry, Centre for Primary Care and Public Health<br>Birch, Judy; Pelvic Pain Support Network<br>Dodds, Julie; Barts and The London School of Medicine and Dentistry, Women's Health Research Unit<br>Cantalapiedra Calvete, Clara; Barts Health NHS Trust, United Kingdom, Department of Obstetrics and Gynaecology<br>Taylor, Stephanie; Queen Mary University of London, Centre for Primary Care and Public Health<br>Rivas, Carol; University College London

Keywords:	Chronic pelvic pain, mHealth, mindfulness, patient engagement, health app, feasibility study

SCHOLARONE™
Manuscripts

I, the Submitting Author has the right to grant and does grant on behalf of all authors of the Work (as defined in the below author licence), an exclusive licence and/or a non-exclusive licence for contributions from authors who are: i) UK Crown employees; ii) where BMJ has agreed a CC-BY licence shall apply, and/or iii) in accordance with the terms applicable for US Federal Government officers or employees acting as part of their official duties; on a worldwide, perpetual, irrevocable, royalty-free basis to BMJ Publishing Group Ltd ("BMJ") its licensees and where the relevant Journal is co-owned by BMJ to the co-owners of the Journal, to publish the Work in this journal and any other BMJ products and to exploit all rights, as set out in our licence.

The Submitting Author accepts and understands that any supply made under these terms is made by BMJ to the Submitting Author unless you are acting as an employee on behalf of your employer or a postgraduate student of an affiliated institution which is paying any applicable article publishing charge ("APC") for Open Access articles. Where the Submitting Author wishes to make the Work available on an Open Access basis (and intends to pay the relevant APC), the terms of reuse of such Open Access shall be governed by a Creative Commons licence – details of these licences and which Creative Commons licence will apply to this Work are set out in our licence referred to above.

Other than as permitted in any relevant BMJ Author's Self Archiving Policies, I confirm this Work has not been accepted for publication elsewhere, is not being considered for publication elsewhere and does not duplicate material already published. I confirm all authors consent to publication of this Work and authorise the granting of this licence.

For peer review only

Mhealth – Using a Mindfulness App for women with chronic pelvic pain: Qualitative data analysis of user experience and lessons learnt

Ball, Elizabeth
Department of Obstetrics and Gynaecology, Barts Health NHS Trust, United Kingdom, Women's Health Research Unit, Barts and the London School of Medicine and Dentistry, Queen Mary University of London, United Kingdom, Centre for Maternal and child Health Research, City University London

Newton, Sian
Centre for Primary Care and Population Health, Queen Mary University of London, United Kingdom

Rohricht, Frank
East London NHS Foundation Trust, United Kingdom

Steed, Liz
Centre for Primary Care and Population Health, Queen Mary University of London, United Kingdom

Birch, Judy
Pelvic Pain Support Network, UK

Dodds, Julie
Women's Health Research Unit, Barts and the London School of Medicine and Dentistry, Queen Mary University of London, United Kingdom

Cantalapiedra, Clara
Department of Obstetrics and Gynaecology, Barts Health NHS Trust, United Kingdom

Taylor, Stephanie JC
Centre for Primary Care and Population Health, Queen Mary University of London, United Kingdom

Rivas, Carol
Department of Social Science, University College London, United Kingdom

Corresponding author

Carol Rivas, Department of Social Science, University College London, United Kingdom
E-mail: c.rivas@ucl.ac.uk

For peer review only - http://bmjopen.bmj.com/site/about/guidelines.xhtml

Abstract

Objective

To determine whether a pre-existing smartphone App to teach Mindfulness Meditation is acceptable to women with chronic pelvic pain (CPP) and can be integrated into clinical practice within NHS CPP pathways. To inform the design of a potential randomised clinical trial.

Design

A pre-study patient and public involvement (PPI) group to collect feedback on the acceptability of the existing app and study design was followed by a three-arm randomised feasibility trial. In addition, we undertook interviews and focus groups with patients and staff to explore app usability and acceptability.

Setting

Two gynaecology clinics within Barts Health NHS, London, UK.

Participants

Patients with CPP lasting ≥6 months with access to smartphone or PC and understanding of basic English.

Intervention

The intervention was mindfulness meditation content plus additional pain module delivered by smartphone app, active controls received muscle relaxation content by the same app. Passive (waiting list) controls received usual care.

Main outcome measures

Themes on user feedback, app usability and integration and reasons for using/not using the app.

Results

App use was low in both active groups.

Patients in the pre-study PPI group, all volunteers, were enthusiastic about the app (convenience, content, portability, flexibility, ease of use). Women contributing to the interview or focus group data (n=14), from a 'real world' clinic, (some not regular app users) were less positive, citing as barriers lack of opportunities/motivation to use the app, and lack of familiarity

For peer review only - http://bmjopen.bmj.com/site/about/guidelines.xhtml

For peer review only

and capabilities with technology. Staff (n=7) were concerned about the potential need for extra support for patients and staff and considered the app needed organisational backing and peer acceptance.

Conclusion

The opinions of pre-study PPI volunteers meeting in their private time may not represent that of patients recruited at a routine clinic appointment.

It may be more successful to co-design/co-develop an app with typical users than to adapt existing apps for use in real-world clinical populations.

Trial registration and funding

The trial (ISRCTN 10925965) was funded by the UK National Institute of Health Research, Research for Patient Benefit programme (RfPB PB-PG-1013-32025).

Keywords

Chronic pelvic pain, mHealth, mindfulness, Headspace, PPI, patient engagement, feasibility study, health app

For peer review only

For peer review only - http://bmjopen.bmj.com/site/about/guidelines.xhtml

Article Summary; 'Strengths and limitations of this study' (relating to methods)

- The study was designed with the help of a study design group of CPP patients
- Recruitment to the study was good
- The qualitative analysis suggests low acceptability which has implications for evaluations of efficacy
- In depth interviews with participants give learning points for future apps, indicating apps co-designed with patients may be preferable for use within health care than modified commercial apps
- Participant feedback and recruitment showed that the research process per se was successful and can be used in the future.

The original protocol for the study has been published separately (1). The UK National Institute of Health Research supported this work (RfPB PB-PG-1013-32025). There are no competing interests.

Introduction

Smartphone health apps, as one form of mhealth (2), are popular in the UK, our study setting, with more than two-thirds of the UK population using smartphones (3,4,5). Health apps are one of the fastest growing app categories, thus numbers of users are still increasing (6). Currently these apps are usually developed either by researchers or (in the majority) by commercial companies, without collaboration between these groups (7,8). The lack of interaction between researchers and commercial developers in the field of pain-related apps has led to a situation where commercially available apps have not been scientifically validated and apps that have been developed from research projects are not commercially available (9).

We were interested in using an app to support women with chronic pelvic pain (CPP) in a

For peer review only - http://bmjopen.bmj.com/site/about/guidelines.xhtml

clinical setting, where validation of an intervention is important to ensure best care. CPP is defined as a subjective physical and emotional experience of pain in the pelvic area that has been present for at least six months that may or may not have an identifiable pathology. CPP affects up to 24% of women worldwide (10) and accounts for 20% of gynaecological clinic referrals. (11,12) It has considerable impact on patients' quality of life and their income (13), and annual costs to the NHS have been estimated at approximately £326 million.(14) CPP is especially common in younger women, who may be categorised as digital natives, making an app-based intervention particularly appropriate. Despite costly interventions, CPP is often resistant to surgical and medical treatment and appears to respond better to a multimodal, holistic approach, (15) with a focus on coping strategies. Apps with such a focus have been shown to be beneficial in various conditions (16). As one example, evidence from uncontrolled trials (17,18) suggests positive effects of mindfulness meditation (MM) as a coping strategy in CPP. We therefore chose to evaluate MM delivered via an app to women with CPP as our intervention.

Mindfulness meditation (MM) depends on activating the psychological state of mindfulness. This refers to an awareness that emerges by way of paying attention intentionally and non-judgementally, in the present moment, to the unfolding of the moment-by-moment experience. Generally two main complementary approaches have been used for mindfulness meditation 1) exercises focusing attention and 2) monitoring of experiences in the present moment. While systematic reviews show that mindfulness meditation may have positive effects on depression, quality of life and pain symptoms in patients with chronic pain (16,19,20) none of the reviewed papers included meditation delivered via mobile phone apps or women with CPP. Evaluation of an existing app is often appropriate (21) and is both quicker and more cost-effective than designing an app from scratch. We chose to evaluate an existing commercial app platform that teaches mindfulness by guided meditation (Headspace ®), with a ten day basic meditation module followed by a pain module specifically designed for MEMPHIS, the name we gave to the study. The Headspace app was publicly nominated favourite health app

For peer review only - http://bmjopen.bmj.com/site/about/guidelines.xhtml

of 2013,(22) has a 5 star user rating in the Apple™ app shop and has scored top in a systematic review (23) of 23 mindfulness apps using the Mobile Application Rating Scale (visual aesthetics, engagement, functionality or information quality). Headspace has reportedly seen over 15 million downloads up to mid-2018.(24)

We undertook a feasibility study to assess whether or not to proceed with a full randomised controlled trial of a modified Headspace meditation app for women with CPP. In this paper we report on the qualitative interview and focus group data from this study; the protocol and quantitative results have been published/ submitted (1,25).

Methods

The MEMPHIS trial was a three-arm parallel randomised feasibility trial approved by Camden and Kings Cross Research Ethics Committee in 2016 (15/LO/1967). Objectives for the qualitative part of this study were to consider:

1) The acceptability, use and usability of the app in the intended service user population and for health care professionals (doctors, health care assistants, clinical and research nurses)

2) The feasibility of integrating such an app into existing healthcare pathways.

Patient and Public Involvement

We held a Patient and Public Involvement (PPI) group workshop before the study to discuss acceptability of the Headspace app and help us design our study. Women attending the Royal London Hospital CPP clinic were invited to volunteer to use the unmodified Headspace app for a week and then feed back in an evening discussion group. Women were not involved in the design of the modified app. Two patient representatives provided support from the study design stage through recruitment to the interpretation of the results and regularly attended Trial Management Group meetings.

Study recruitment

For peer review only

The trial recruited at two outpatient gynaecology clinics within Barts Health NHS trust in two separate deprived areas of inner East London. Female patients with new or follow-up gynaecology appointments were assessed for eligibility by a researcher in clinic, having been posted a Patient Information Sheet. Women were eligible if they had been suffering with CPP for 6 months or more and had at least a basic understanding of the English language. Women were excluded if they did not meet these criteria or they did not have access to a smartphone or personal computer or were currently using the Headspace app (there were very few of the latter). All patients gave full and informed consent to be randomised and data was collected through all stages of the study.

For the study of quantitative data, 90 patients were allocated randomly in a 1:1:1 ratio to the mindfulness meditation app, a muscle relaxation app active control or the usual care arm. Patients in the two active arms were asked to download the modified app in the clinic with support from a research staff member and were sent a questionnaire about app usability, an analysis of which is reported in a companion paper. (25) We used these data to inform topic guides for the qualitative part of the study, i.e. our outlines of key issues and areas of questioning that were used to guide our semi-structured interviews and focus groups with patients and staff.

Interviews and focus groups

All patients in the two active arms were invited to one of two focus groups at their own study site after the 6-month follow up. We offered telephone interviews as an alternative. All staff participating in the study were invited to attend a staff focus group overseen by the patient representative and facilitated by a researcher. Qualitative outcomes included feedback on app usability and acceptability. In addition, members of the staff focus group (doctors, health care assistants, clinical and research nurses) were asked about the ease of integration into existing NHS pathways. Part of the staff discussion was free flowing with open-ended questions, and part was structured using questions modified from the Normalisation Process

For peer review only - http://bmjopen.bmj.com/site/about/guidelines.xhtml

Theory (NPT) toolkit; NPT is a theory of implementation practices (26). Patients were also asked to 'walk through' the app with researchers and comment on its different specific features, (27) as well as feeding back on our study process. Walkthroughs are often used in developing technologies such as mhealth; patients 'walked through' use of the app, articulating their thought processes while they did so (27). This helped to identify issues or barriers to use of the app from the users' points of view without the need for technical discussions. Results for the walkthrough, showing comments on different features specific to the usability of the intervention app used in our study are shown in Appendix 1; walkthroughs were undertaken by two patients. All data were audio-recorded at point of collection and transcribed, with personal identifying data removed from transcripts. Raw data were stored in a Primary Care Clinical Trial Unit database to clinical trial standards.

Analysis

Analysis was carried out blinded as to which study app was used, and deployed the immersion-crystallisation method (28). Thus, the lead qualitative researcher immersed herself in the data, reading transcripts carefully, then writing down articulated or crystallised patterns or themes that related to the aims and research questions of the study. These were discussed with another researcher from the team, and themes modified as appropriate. This process was repeated until all the data had been examined and all patterns that had been noticed were articulated, discussed and substantiated with exemplar extracts. This approach was considered appropriate since we had a small dataset and we were not aiming to develop conceptual themes but rather to inform the design and development of a randomised controlled trial for the modified app.

We used the SRQR checklist when writing our report (29).

For peer review only

Results

We screened 488 women between May and September 2016 for their eligibility to participate in the study. After exclusions, 90 women gave full consent to participate and were randomised to the intervention arm (31 women), the active control arm (30 women) or the usual care arm (29 women).

Demographics

Women were aged a mean of 35 years, 66% were employed and overall approximately 50% had stayed in full time education until at least the age of 20 though the proportion was least, at 36.7% in the intervention arm. Overall 44% were of white ethnicity though the proportion was lowest in the intervention arm (35.7%) and highest in the usual care arm (53.6%). The second most common self-reported ethnic groups were 'Southern Asian' and 'Black'. Women in the intervention arm were most likely to have experienced CPP for 3-5 years (40.3% of this group), women in the usual care group for over 10 years (42.9% of this group). More women had pain for longer than two years in the intervention arm than in either of the other two arms. All women reported a high pain intensity, with means of 6.8 to 6.9 in the previous week (on a scale from 1-10). Other demographic data are reported in Forbes *et al*. (25) Our qualitative sample was taken from the two active arms but we did not record separate demographic data for the women in this smaller sample.

For peer review only - http://bmjopen.bmj.com/site/about/guidelines.xhtml

Pre-study PPI group

The ten women in the pre-study PPI group were self-selected local women who were familiar with using apps. They anticipated no technical issues even in women who were not used to apps. They considered that the Headspace app would be successfully adopted by patients taking part in the study, given that a smartphone, like CPP, is ‘always with you’. They praised the flexibility of the app, welcomed its portability and were unanimous in saying it was easy to slip off for 10 minutes when at work to use it. As a result, they found they could use it at times when they most needed pain relief as well as to prevent pain and found the app helpful in relieving pain and stress. The group reported being able to meditate without the app, once they had tried it with the app; however, they still preferred to use the app because they found the voice soothing.

App usage in the study

Patient adherence to the app was less than expected from our pre-study PPI group discussions. (25) Only 36% of meditation app patients and 46% of the active control patients used the app at least once. (25)

Thematic analysis

Qualitative data were obtained from 14 patients; 12 preferred a telephone interview, two attended face-to-face interviews, one participant at the University attached to one of the recruiting clinics and one participant at the other recruiting hospital. Patients chose not to attend focus groups. Seven people attended the staff focus group: two recruiting nurses, three clinic nurses one consultant and a representative from the pelvic pain support network.

The qualitative analysis revealed three main themes from all participants combined (the analyst remained blind to app group) regarding usability, and four subthemes:

1. Familiarity and capabilities with app technology
2. Motivations to use the app
    - Perceived benefits

For peer review only - http://bmjopen.bmj.com/site/about/guidelines.xhtml

   - Relation to other therapies

3. Opportunities to use the app:
   - Technology issues getting in the way
   - Life getting in the way

These are explored below.

Familiarity and capabilities with app technology

Around half of the patients were sufficiently familiar with technology and apps to be comfortable using the study apps. However, six participants reported difficulty with them because they were *"not very good at technology*", or were unsure how to get started or use the app effectively without help.

> *I am not good with technical some things that is why the problems I had arisen, right okay. So I consulted with my daughter and she helped me work it out… so I don't try everything. (patient 1002)*

One patient *(1001)* was not used to technical app language; 'help' suggested emotional support to her, for example. Two more changed handsets and therefore did not continue with the app. In all cases these technical difficulties appeared to lead to abandoning of the app or restricted use of its functionality.

Five patients having technical problems suggested possible solutions such as a 'class' or group for first time users, a YouTube orientation video, or a pictorial leaflet. This might include an introduction to meditation and mindfulness as well as the app itself. One woman commented: *"If your market is targeting people who are not using apps then you are going to have to get together and find ways to do this",* (patient 1041); she also suggested we could get ideas from other apps on the market in this regard.

For peer review only - http://bmjopen.bmj.com/site/about/guidelines.xhtml

Given their experiences in the study, staff were concerned about additional staff time needed to support women in using the app. This would sit in tension with one of the original rationales behind choosing an app as the mode of delivery, which was to increase the effective use of staff contact time with patients. Language barriers might compound problems.

Motivations to use the app

Staff, though unaware of the low sustained app use in the study, felt it would be common sense to hold occasional motivating meetings with patients if the intention was for them to use the app long term. The patient data suggested the main motivators or lack of motivation for using the app in our sample, which could be drawn on in such meetings, and which we now consider.

Perceived benefits

Three patients said they only entered the trial to help others through research but were already using alternative forms of pain control. They explained that this meant they were not motivated to actually use the app, perceiving the relative benefit to be small. The failure of such altruism to extend to using the app is a recognised phenomenon in clinical trials that has been called 'weak altruism'. (30) Thus, only one of these three patients persevered. Even though she was one of the women who experienced difficulties with the technology, she explained, "*with something that is as soul destroying as the pain, it is important to help others off the back of other people's misfortune as it were"* (1036). However, she wondered how relevant her data were:

I took steps to improve my situation from a weight loss perspective as well and I've lost a lot of weight which has significantly helped not 100% but it is has significantly helped so I felt a bit fraudulent the last time filling in the forms because , so everything had improved so much

For peer review only - http://bmjopen.bmj.com/site/about/guidelines.xhtml

so from the medical study perspective it was more about the weight loss than the app. So I felt a bit bad that I was still taking part. (patient 1036)

There was no clear pattern regarding the impact of current pain on app use by patients. Six said they used it regardless of pain intensity – sometimes developing a daily routine - while four only used it when in severe pain or expecting to be (e.g. during menstruation). This cyclical or intermittent use in some patients should be considered when looking at our study outcomes.(25) The Headspace app requires regular use to learn and benefit from psychological techniques. To address this, healthcare professional alerts have been effective in other studies, (31) whilst Headspace only has a reminder function that the user can set. This was often not sufficient, as one patient said even with this feature, "*To be quite honest I used it a couple of times and then forgot. And then I [remembered it and] used it more frequently." (patient 1036)*

One patient said medication was not working but the app did, though she was not sure whether this was 'mind over matter' (1065), which was her term for a placebo effect. Three others said it did not reduce their pain. Other benefits were considered good reason for using the app even when participants did not feel that it reduced pain intensity. Alternative or unanticipated benefits were not formally measured or taken into account in the study's effectiveness outcomes (25). For example, ten patients valued the way the app helped them to relax or de-stress or focus and re-assess their life; three of these specifically said they used it to induce a relaxed state to get to sleep. Notably the active control was a relaxation app, so it may be that most of the patients interviewed were in this study arm; we were blind to this. One participant said she did not like the focus on pain per se as her condition impacted on various areas of her life. Even when the app was positively received, women might stop using it because it was too powerful, and they had gained the change they wanted:

I think it was day 3, I could see the change that was happening, I was able to speak up for myself ……I can't explain it, even now I am getting emotional… it's just a lack of focus, I just needed direction. To try and put It into words. To me it meant so much that I have gone back

For peer review only - http://bmjopen.bmj.com/site/about/guidelines.xhtml

to church… I use it outside of the app now I have got from it what was missing, so it's done something to me and for me which is very positive, and I may try it to lose weight but those positive vibes are still there. I can't go back to it because I did not want to go any further because what I got at the time helped me to focus, to change my way of thinking. I used it for about two or three weeks. (patient 1001)

Three patients found the app put them more in tune with their bodies and their breathing, (two of these were among those who also found the app de-stressing) while another found yoga better for that. Six patients, like pre-study PPI group members, also learned to use techniques from the app to alter their stress patterns without the app, having tried it, for example in traffic or by sitting down and taking time out or for general relaxation. Four of these came from the group of ten patients that reported de-stressing as the app's main benefit, and it is not clear whether they had the intervention app or the active control.

Relation to other therapies

Two patients preferred 'pure' meditation. Another considered the app to be "*very much about meditatio*n". It may be that the former two were in the active control and the latter in the intervention arm. An alternative therapy practitioner and two further patients reported that they preferred yoga; one said this was because it focussed on each part of the body in turn.

Three patients thought the app was useful as an adjunct to other methods rather than a replacement for them, for example physical interventions such as Pilates, or listening to classical music.

Opportunities to use the app

Technology issues getting in the way

Staff pointed out that not all patients had smartphones (not appreciating that PCs/tablets were alternatives allowed in this study). Some patients lacked the storage space to load the app on their phones. There were also issues with Wi-Fi connectivity when staff tried to help the patients load the app within the hospital sites. Possible solutions that staff suggested were to

For peer review only - http://bmjopen.bmj.com/site/about/guidelines.xhtml

lend patients phones and to have group upload sessions in a location with good Wi-Fi signal – though they acknowledged the resource implications.

Life getting in the way

Seven patients revealed they preferred to use the app in the evenings because of other life commitments. This meant they did not always use it as a direct response to pain, reducing its potential for contemporaneous effect. One patient who used it in response to pain but only used it once or twice blamed this on having no spare time because of juggling work and children; however, another patient managed despite such commitments.

Barriers to integration for staff

Staff believed that the biggest barrier to clinical adoption of the app was a possible lack of support from the host organisation. It might also be hard to integrate the app within existing professional work practices if the staff in the position of offering the app to patients failed to see its relative advantage over other interventions. Collection of feedback on the app's effectiveness would be necessary for staff to support sustained use. It was felt that staff would need training on how to introduce the app to women in practice, and that complexity and high staff turnover could impede sustained use. An app was also seen as impersonal compared with face-to-face contact, which was more favoured by staff.

Participant comments on the research process

The study questionnaires that were used for the main quantitative outcome measures (25) were acceptable to patients except for some discomfort with a question about sex, which patients considered a delicate question that was missing a 'no sex' option. Most preferred a paper form reflecting their lack of affinity with technology. There were no indications that the study design or study processes had contributed to the participants' lack of engagement with

For peer review only - http://bmjopen.bmj.com/site/about/guidelines.xhtml

the apps. A full summary of patient comments on the study design and procedures is given in Appendix 1.

Discussion

Our study adds to the limited evidence on mHealth app user behaviour and experience (32,33). We were able to explore reasons for low app usage in our feasibility study.(25) The thematic analysis of qualitative data from this study suggests that the low app use in the trial occurred because many patients were not familiar with apps in general or lacked capabilities with technology. Women also stated limited motivation to use the app because of a lack of perceived benefit, or a lack of opportunity to use the app due to Wi-Fi issues or due to other commitments.

Similar findings were reported by Laurie *et al*.(33) who interviewed 16 healthy city-dwelling participants (25-38 years) about their user behaviour before and after 30-40 days of Headspace app exposure. Like us, they reported barriers of busy lives, failure to establish a routine and a lack of perceived benefit; all users in their study tried the app at least once hoping it could deliver a quick fix but were disappointed if this did not happen. In our study many patients failed to perceive a benefit from using the app. Hence excuses stating other commitments may mask a deeper lack of motivation linked to perceptions of benefits.(34)

The advantages and disadvantages of using the app stand-alone were also illustrated by our data. Some suggestions made by participants to improve usage, such as more guidance at the start, seem obvious in hindsight. But they had not been considered because of the feedback from the pre-study PPI group and the commercial success of Headspace. The use of community contacts may be a helpful alternative.(35) Social support can create a community of practice, help to clarify expectations,(36) and improve health outcomes (as shown for example in internet based psychological treatment for depression (37).

For peer review only - http://bmjopen.bmj.com/site/about/guidelines.xhtml

The data suggest that for successful app use we need to understand what motivates individuals with clinical need to use the app and target this, for example by setting appropriate expectations. Incentivisation might also improve motivation. This could be achieved through app gamification (8), or encouragement through integration with patient-clinician face-to-face encounters, which was lacking in our study since the app was used stand-alone. The present study provided extensive technical support but no coaching and incentivising, in keeping with the protocol. Future app studies should take this into account. Participants in our study may have also have benefitted from training and support to improve their app use capabilities and guidance on how to create more opportunities for app use – such as through sharing experiences in clinic support groups. This is in keeping with the COM-B model of behaviour change (38) which our themes matched, though this was only realised after analysis. The COM-B model says that Capability, Opportunity and Motivation are key drivers of behaviour and has been used to develop a number of complex interventions including smartphone apps (e.g. 4).

Lack of engagement after recruitment, or good initial engagement but minimal or inconsistent use, have been reported in other studies (39,40), including in Headspace trials other than our own (41, 42). Settings were a university and a psychiatric inpatients clinic, both in the USA. Inconsistent app use was noted by Wen (43) in junior doctors who used self-guided Headspace. Morrison Wylde (44) compared face-to-face MM with headspace use in novice paediatric nurses. Although, unlike our study there were no dropouts/non-users and also no record of whether or how long the app was used for which is an important omission.

None of these studies included a qualitative component. Yet, each of the Headspace study groups were very different, and so will likely have differed in motivations, contexts for opportunity to use the app, and incentivisations (45). While these aspects were not considered in the other studies, we have been able to do so. Our findings suggest these are important considerations in any study of app use and therefore this study makes a contribution to the

For peer review only - http://bmjopen.bmj.com/site/about/guidelines.xhtml

field. For example, some of the groups in other studies may have differed from ours in likelihood of using mhealth apps in the first place, and familiarity with technology. In-patients may have more time to use the app and more support – and may also have had specific barriers to app use, such as related to setting and to illness.

Patients in the qualitative part of our study tended not to use apps on a regular basis (or at least apps other than simple games), and in terms of our themes, also represented in the COM-B model, may be said to have few capabilities in technology use. They therefore do not represent the typical users of the Headspace app in a commercial setting. Accessing the app regularly requires energy, time and effort, but patients with CPP often suffer from fatigue and anxiety as co-morbidities, perhaps whilst having to juggle family life and work. Therefore, this may be seen as a challenging clinical population in which to trial an app. Further Headspace trials with diabetic (NCT03274362) and pain **(**NCT03495726**)** outpatients are underway.

Our study has also shown that clear consideration of unexpected benefits should be included in future studies and these can be informed by our finding that benefits for patients may be more diffuse than anticipated (e.g. app relieving stress rather than pain). Though we were blind as to whether the patients we interviewed were in the active arm or the intervention arm of the study, there is indication from responses (especially in the patients who did a walkthrough) that many with these extra benefits were in the intervention arm. Our data also suggest that staff benefits may be less than anticipated, as participants sometimes needed a lot of support and scaffolding in technology use at least initially.

Young age, co-morbid anxiety and low educational attainment are predictors for dropping out of web-based interventions according to studies in the field of depression. (46,47) This may be true despite regular phone support (47) though our participants all considered active motivational support from staff or app support groups would have improved app use. Our

For peer review only - http://bmjopen.bmj.com/site/about/guidelines.xhtml

intervention arm participants were particularly likely to be young and with low educational attainment.

Our data suggest that it is important to involve real world end users in the agile design or development or modification of apps in close collaboration with researchers and commercial app developers.(8) Although the evaluation of existing apps has been recommended as a cost-effective and rapid process, (21) our findings suggest that in actual clinical practice these may be problematic.

Strengths and weaknesses of the study and in relation to other studies

One strength of this study is that it creates much-needed evidence in the field of evaluating existing health apps in a clinic population (7,9,21) and recording user experience. This provides us with lessons to be learned.

Researchers conducting interviews and focus groups were: a senior mixed methods medical sociology researcher, a recruiting nurse, a representative from the pelvic pain support network and an experienced health psychologist. Findings were similar across the data and the different backgrounds of the researchers therefore does not appear to have influenced findings. The main analysis was undertaken by the medical sociologist and so the concordance with the COM-B model is not due to background discipline bias.

We were able to recruit successfully, and we obtained valuable information from patients with CPP, who were recruited from a deprived urban area of the UK as typical local clinical patients. However we report a marked discrepancy between the attitudes of the pre-study PPI group of volunteer patients from the local area, who actively put themselves forward for a 7-day trial of the app, and the participants asked to take part when they attended clinics. The opinions of pre-study PPI volunteers meeting in their private time may not be representative of the opinions of patients recruited at a routine clinic appointment. Women in the PPI group were used to using apps, which had led them to be interested in the study in the first place. Whereas

For peer review only

women in the PPI group had all trialled the app at home and work without support from us, many patients from clinic were unable to use their phone beyond calls, texts and photos.

To our knowledge the present observation on failure of PPI work to translate into practice in a trial has not been formally reported before, and is lacking from a recent comprehensive systematic review.(48) PPI involvement is a stipulated requirement when applying for some funding, and the present research findings should be taken into account when drafting guidelines for future PPI involvement in study planning. PPI groups are able to provide significant help and advice in any study but our findings shows the value of adding agile co-development as a requirement for app intervention development as likely to provide a more effective intervention than one informed by PPI alone.

Implications for clinicians and policymakers

Given the patchy use of the app and the way that some participants did not manage to unlock its full functionality, and an indication of diffusion of benefit, more work is needed to see whether the app reduces pain per se. This study is a good example of the need to move away from 'one size fits all' behavioural interventions. Future studies should do more work on implementation before doing an effectiveness trial. This will enable researchers to be more nuanced about saying who the app is effective for, if at all.

Strategies to involve busy, less motivated, and less technologically experienced individuals in PPI and lay app design groups need to be further developed. These groups should include considerable scaffolding, which we have shown extends to study involvement by patients. More care is also needed to obtain PPI input that is representative of the study group with typical users of the target group, taking into account their capabilities, opportunities and motivational aspects. Moreover, we can confirm a recent review suggesting that health apps

For peer review only - http://bmjopen.bmj.com/site/about/guidelines.xhtml

should be co- designed with users (8), rather than presenting them with a pre-existing app. These implications for our study are also generalisable to other technology studies.

Our study did not show the app was ineffective but rather that we could not show any possible effectiveness. Thus, further work is needed on MM app effectiveness in clinical subgroups such as our local users, bearing in mind also that diffuse benefits were found.

Research in context

What is known

1. Chronic pelvic pain has a large impact on patients and the NHS and is difficult to treat.
2. Health outcomes are improved by psychological and lifestyle interventions but are often not addressed due to difficult access or service shortages.
3. Mindfulness meditation has not been investigated in chronic pelvic pain patients but randomised controlled trials show improved health outcomes in other chronic pain conditions.
4. Mhealth apps are increasingly popular.

What the study adds

1. A mindfulness meditation app may not necessarily be taken up by patients with CPP even when it is a commercial success in the general population. Considerable supportive scaffolding may be needed.
2. Use of existing commercial apps in actual clinical practice may be problematic compared with the agile development of apps with collaboration between researchers, clinicians, developers and end users/

For peer review only - http://bmjopen.bmj.com/site/about/guidelines.xhtml

3. Chronic Pain patients are interested in alternatives to drug or surgical treatments and further research is required in this area, including with MM, the benefits of which may extend beyond pain relief itself.
4. PPI groups may be more motivated to use an intervention than a real world clinical group as they are volunteers who are interested in the research topic. This may be particularly problematic for interventions that require considerable capability or motivation in use.

Auhthor contributions

EB, CR, JB, JD, BK, ST, ES, FR, BK, SN and KK contributed to the study design and initial protocol.

CC was a researcher and contributed to the study design

BK and GF provided statistical support and ran the statistical analysis.

CR and LS led the interview and focus group field work and analysis. All authors provided support throughout the trial and contributed towards the final paper.

Role of the funding source

The UK National Institute of Health Research, Research for Patient Benefit (RfPB No. PB-PG1013-32025) funded the MEMPHIS study. The funder had no role in the study design, in the collection, analysis, and interpretation of the data, in the writing of this report, or in the decision to submit the paper for publication. The first and last authors vouch for the integrity, completeness and accuracy of the data and analyses, and for the fidelity of this report to the protocol and statistical analysis plan. The views and opinions expressed herein are those of the authors and do not necessarily reflect those of the RfPB, NIHR, NHS or the Department of Health.

Competing interests

We have read and understood BMJ policy on declaration of interests.

Data sharing

No additional data available.

Transparency

For peer review only - http://bmjopen.bmj.com/site/about/guidelines.xhtml

The lead author confirms that the manuscript is an honest, accurate and transparent account of the study being reported; that no important aspects of the study have been omitted; and that any discrepancies from the study as planned and registered have been explained.

Acknowledgements

We would like to thank all the researchers, consultant obstetricians and gynaecologists and data assistants at each of the recruiting clinics for their hard work in promoting the study, recruiting participants and for data entry. Our thanks go to the Trial Steering Committee; Andrew Horne, Sohinee Bhattacharya, Christina Liossi, and Hulya Guzel for their constant support throughout the trial.

We thank the Pelvic Pain Support Network and Endometriosis UK for their promotion and guidance in developing the study design. We would also like to acknowledge the NIHR RfPB programme for their on-going support.

Lastly, thank you to Headspace Ltd for providing our participants with access to the Headspace platform, designing novel content for the study, and for their continuous support and advise throughout the study.

References

1. Ball E, Newton S, Kahan BC, Forbes G, Wright N, Cantalapiedra Calvete C, et al. Smartphone App Using Mindfulness Meditation for Women With Chronic Pelvic Pain (MEMPHIS): Protocol for a Randomized Feasibility Trial. JMIR Res Protoc. 2018;7(1):e8.
2. Ali EE, Chew L, Yap KY.Evolution and current status of mhealth research: a systematic review. BMJ Innovations 2016;2:33-40
3. Ofcom. The Communications Market Report. Ofcom: United Kingdom 2015. https://www.ofcom.org.uk/research-and-data/multi-sector-research/cmr/cmr15/uk [accessed 21 Macrh 2019]
4. Kayyali R. Peletidi A. Ismail M, Hashim Z. Bandeira P, Bonnah J. Awareness and Use of mHealth Apps: A Study from England. Pharmacy 2017, 5, 33.
5. Sezgin E, Yildirim S, Özkan-Yildirim S, Sumuer E. Current and Emerging mHealth Technologies: Adoption, Implementation, and Use: Springer International Publishing; 2018 2018.
6. Lunden I. 6.1B Smartphone Users Globally By 2020, Overtaking Basic Phone Subscriptions https://techcrunch.com/2015/06/02/6-1b-smartphone-users-globally-by-2020-overtaking-basic-fixed-phone-subscriptions/?guccounter=12015 [cited 2018 28 August 2018].
7. Subhi Y, Bube SH, Rolskov Bojsen S, Skou Thomsen AS, Konge L. Expert Involvement and Adherence to Medical Evidence in Medical Mobile Phone Apps: A Systematic Review. JMIR Mhealth Uhealth. 2015;3(3):e79.
8. Edwards EA, Lumsden J, Rivas C, et al. Gamification for health promotion: systematic review of behaviour change techniques in smartphone apps. BMJ Open. 2016;6(10):e012447. doi:10.1136/bmjopen-2016-012447.

For peer review only - http://bmjopen.bmj.com/site/about/guidelines.xhtml

9. de la Vega R, Miro J. mHealth: a strategic field without a solid scientific soul. a systematic review of pain-related apps. PLoS One. 2014;9(7):e101312.
10. Latthe, P., Latthe, M., Say, L., Gülmezoglu, M., & Khan, K. S. (2006). WHO systematic review of prevalence of chronic pelvic pain: a neglected reproductive health morbidity. BMC public health, 6, 177. doi:10.1186/1471-2458-6-177
11. Ayorinde AA, Macfarlane GJ, Saraswat L, Bhattacharya S. Chronic pelvic pain in women: an epidemiological perspective. Womens Health (Lond). 2015;11(6):851-64.
12. Howard F. The Role of Laparoscopy in Chronic Pelvic Pain: Promise and Pitfalls. Obstetrical & Gynecological Survey. 1993;48(6):357-87.
13. Zondervan KT, Yudkin PL, Vessey MP, Jenkinson CP, Dawes MG, Barlow DH, et al. Chronic pelvic pain in the community--symptoms, investigations, and diagnoses. Am J Obstet Gynecol. 2001;184(6):1149-55.
14. Curtis L. Unit Costs of Health and Social Care 2014 Personal Social Services Research Unit, University of Kent, Canterbury.: University of Kent, Canterbury; 2014 [Available from: https://www.pssru.ac.uk/project-pages/unit-costs/unit-costs-2014/.
15. Peters AA, van Dorst E, Jellis B, van Zuuren E, Hermans J, Trimbos JB. A randomized clinical trial to compare two different approaches in women with chronic pelvic pain. Obstet Gynecol. 1991;77(5):740-4.
16. Ball EF, Nur Shafina Muhammad Sharizan E, Franklin G, Rogozinska E. Does mindfulness meditation improve chronic pain? A systematic review. Curr Opin Obstet Gynecol. 2017;29(6):359-66.
17. Kold M, Hansen T, Vedsted-Hansen H, Forman A. Mindfulness-based psychological intervention for coping with pain in endometriosis. Nordic Psychology. 2012;64(1):2-16.
18. Fox SD FE, Allen RH. Mindfulness meditation for women with chronic pelvic pain: a pilot study. J Reprod Med 2011;56(3-4):158-62.
19. Plaza I, Demarzo MM, Herrera-Mercadal P, Garcia-Campayo J. Mindfulness-based mobile applications: literature review and analysis of current features. JMIR Mhealth Uhealth. 2013;1(2):e24.
20. Hilton L, Hempel S, Ewing BA, Apaydin E, Xenakis L, Newberry S, et al. Mindfulness Meditation for Chronic Pain: Systematic Review and Meta-analysis. Ann Behav Med. 2017;51(2):199-213.
21. Boudreaux ED, Waring ME, Hayes RB, Sadasivam RS, Mullen S, Pagoto S. Evaluating and selecting mobile health apps: strategies for healthcare providers and healthcare organizations. Transl Behav Med. 2014;4(4):363-71.
22. My Health Apps [Available from: http://myhealthapps.net/app/details/127/Headspace-on-the-go] (Accessed 28 August 2018).
23. Mani M, Kavanagh DJ, Hides L, Stoyanov SR. Review and Evaluation of Mindfulness-Based iPhone Apps. JMIR Mhealth Uhealth. 2015;3(3):e82.
24. App Annie 2018 [Available from: https://www.appannie.com/en/] (Accessed 28 August 2018).
25. Forbes G, Newton S, Cantalapiedra C, Birch J, Dodds J, Steed E, Rivas C, Khan KS, Rohricht F, Taylor SJC, Kahan B, Ball E. A smartphone app using psychological approaches for women with chronic pelvic pain (MEMPHIS): a randomised feasibility trial. submitted
26. Murray E, Treweek S, Pope C, MacFarlane A, Ballini L, Dowrick C, et al. Normalisation process theory: a framework for developing, evaluating and implementing complex interventions. BMC Med. 2010;8:63.
27. Gerhardt?Powals J. Cognitive engineering principles for enhancing human?computer performance. International Journal of Human-Computer Interaction. 1996;8(2):189-211.
28. Borkan J. Immersion/Crystallization. In BF Crabtree and WL Miller (Eds) Doing Qualitative Research (2nd edition). Thousand Oaks, CA: Sage Publication; 1999. p. pp. 179-94.
29. O'Brien BC, Harris IB, Beckman TJ, Reed DA, Cook DA. Standards for reporting qualitative research: a synthesis of recommendations. Acad Med. 2014;89(9):1245-1251.

For peer review only - http://bmjopen.bmj.com/site/about/guidelines.xhtml

30. McCann, S. K., Campbell, M. K., & Entwistle, V. A. (2010). Reasons for participating in randomised controlled trials: conditional altruism and considerations for self. Trials, 11, 31. doi:10.1186/1745-6215-11-31
31. Palmer, M., Sutherland, J., Barnard, S., Wynne, A., Rezel, E., Doel, A., Grigsby-Duffy, L., Edwards, S., Russell, S., Hotopf, E., Perel, P., … Free, C. (2018). The effectiveness of smoking cessation, physical activity/diet and alcohol reduction interventions delivered by mobile phones for the prevention of non-communicable diseases: A systematic review of randomised controlled trials. PloS one, 13(1), e0189801. doi:10.1371/journal.pone.0189801
32. Marcolino MS, Oliveira JAQ, D'Agostino M, Ribeiro AL, Alkmim MBM, Novillo-Ortiz D. The Impact of mHealth Interventions: Systematic Review of Systematic Reviews. JMIR Mhealth Uhealth. 2018;6(1):e23.
33. Laurie J, Blandford A. Making time for mindfulness. Int J Med Inform. 2016;96:38-50.
34. Collins M, Shattell M, Thomas SP. Problematic Interviewee Behaviors in Qualitative Research. Western Journal of Nursing Research. 2016;27(2):188-99.
35. Michie S, Richardson M, Johnston M, Abraham C, Francis J, Hardeman W, et al. The behavior change technique taxonomy (v1) of 93 hierarchically clustered techniques: building an international consensus for the reporting of behavior change interventions. Ann Behav Med. 2013;46(1):81-95.
36. Mohr DC, Cuijpers P, Lehman K. Supportive accountability: a model for providing human support to enhance adherence to eHealth interventions. J Med Internet Res. 2011;13(1):e30.
37. Andersson G. Using the Internet to provide cognitive behaviour therapy. Behav Res Ther. 2009;47(3):175-80.
38. Michie S, van Stralen MM, West R. The behaviour change wheel: a new method for characterising and designing behaviour change interventions. Implement Sci. 2011;6:42.
39. Walsh JC, Corbett T, Hogan M, Duggan J, McNamara A. An mHealth Intervention Using a Smartphone App to Increase Walking Behavior in Young Adults: A Pilot Study. JMIR Mhealth Uhealth. 2016;4(3):e109. Published 2016 Sep 22. doi:10.2196/mhealth.5227
40. Geraghty AW, Torres LD, Leykin Y, Perez-Stable EJ, Munoz RF. Understanding attrition from international Internet health interventions: a step towards global eHealth. Health Promot Int. 2013;28(3):442-52.
41. Mistler LA, Ben-Zeev D, Carpenter-Song E, Brunette MF, Friedman MJ. Mobile Mindfulness Intervention on an Acute Psychiatric Unit: Feasibility and Acceptability Study. JMIR Ment Health. 2017;4(3):e34.
42. Noone C, Hogan MJ. A randomised active-controlled trial to examine the effects of an online mindfulness intervention on executive control, critical thinking and key thinking dispositions in a university student sample. BMC Psychol. 2018;6(1):13.
43. Wen L, Sweeney TE, Welton L, Trockel M, Katznelson L. Encouraging Mindfulness in Medical House Staff via Smartphone App: A Pilot Study. Acad Psychiatry. 2017;41(5):646-50.
44. Morrison Wylde C, Mahrer NE, Meyer RML, Gold JI. Mindfulness for Novice Pediatric Nurses: Smartphone Application Versus Traditional Intervention. J Pediatr Nurs. 2017;36:205-12.
45. Lim D, Condon P, DeSteno D. Mindfulness and compassion: an examination of mechanism and scalability. PLoS One. 2015;10(2):e0118221.
46. Arean PA, Hallgren KA, Jordan JT, Gazzaley A, Atkins DC, Heagerty PJ, et al. The Use and Effectiveness of Mobile Apps for Depression: Results From a Fully Remote Clinical Trial. J Med Internet Res. 2016;18(12):e330.
47. Gilbody S, Lewis H, Adamson J, Atherton K, Bailey D, Birtwistle J, et al. Effect of Collaborative Care vs Usual Care on Depressive Symptoms in Older Adults With Subthreshold Depression: The CASPER Randomized Clinical Trial. JAMA. 2017;317(7):728-37.
48. Brett J, Staniszewska S, Mockford C, Herron-Marx S, Hughes J, Tysall C, et al. A systematic review of the impact of patient and public involvement on service users, researchers and communities. Patient. 2014;7(4):387-95.

For peer review only - http://bmjopen.bmj.com/site/about/guidelines.xhtml

For peer review only

For peer review only - http://bmjopen.bmj.com/site/about/guidelines.xhtml

APPENDIX 1

Feature	Positive aspects	Negative aspects	Solutions
**Voice**	Liked by some participants, considered soothing and even spiritual	The voice was not relaxing. One had to listen carefully which meant you could not switch off; otherwise you could not hear instruction clearly during meditation.<br><br>It was also too repetitive, after 10 times it felt like a chore and not something to look forward to.	Choice of different voices<br><br>participants should be told it takes time to get into the rhythm of the instructions, but you can get used to it
**General interface aesthetics**	colours really fresh, interface not too busy, with pleasing layout and aesthetics	one participant would prefer different, bright, colours such as purple	None needed
**Graphics**		The pictures were often not appropriate as they focused on the problem (e.g. tooth pain logo) rather than something positive or soothing. Unanticipated problems could arise according to one participant; she found it hard to lose weight and felt the food icons a) did not represent success and b) reminded her of eating.<br><br>One participant found the pictures hard to see.	Psychological theory supports the view that positive images would be advisable

For peer review only

**Animations**	liked	poorly chosen	None needed
**Progress**	One participant liked the way the app helped progress through the stages.<br><br>Another participant, with cyclical pain and hence cyclical use, did not aim to progress but simply chose to use the baseline video each time.	Three participants were frustrated and even 'angry' and 'patronised' at having to do the same (basic) meditation several times before moving up a stage.<br><br>There was no indication of goals, duration of each level or gamification rewards.	Goal setting is recommended by psychological theory and could be as simple as using "day 1 of 30" rather than simply Day 1.<br><br>Changing emojis could show progress, for example from a sad face to a smiley face to a heart; the inbuilt progress function was not felt sufficiently motivating.
**Introduction**		Experienced app users suggested the introduction could be improved for initiates	overview of the entire app and its levels suggested for the start.<br><br>One participant had done mindfulness before but thought an introduction to mindfulness might be helpful for others.
**Enjoyment**	considered fun by some participants.	One said it was not very 'interesting'.	Include examples of a patient's day with the app to cater for a greater variety of participants<br><br>It would be good to have other features as drop down options that were fun and

For peer review only

			did not involve meditation.

**Session intensity**		Would be good to choose the duration of the meditation.	15 or 20 mins would be better than 10, which is not enough time shut off and meditate deeply.<br>The option to control this was important as even 10 minutes was a difficult commitment for some busy participants (1074, 1075, 1078) or those battling with chronic fatigue (1074)
**Pain modules**		it can be hard to focus on something like using the app when you are in pain.	None needed

Comments on app acceptability and usability made by n=13 patients. Two patients walked through the app with the researcher

For peer review only

Reporting checklist for qualitative study.

Based on the SRQR guidelines.

Instructions to authors

Complete this checklist by entering the page numbers from your manuscript where readers will find each of the items listed below.

Your article may not currently address all the items on the checklist. Please modify your text to include the missing information. If you are certain that an item does not apply, please write "n/a" and provide a short explanation.

Upload your completed checklist as an extra file when you submit to a journal.

In your methods section, say that you used the SRQR reporting guidelines, and cite them as:

O'Brien BC, Harris IB, Beckman TJ, Reed DA, Cook DA. Standards for reporting qualitative research: a synthesis of recommendations. Acad Med. 2014;89(9):1245-1251.

		Reporting Item	Page Number
	#1	Concise description of the nature and topic of the study identifying the study as qualitative or indicating the approach (e.g. ethnography, grounded theory) or data collection methods (e.g. interview, focus group) is recommended	6
	#2	Summary of the key elements of the study using the abstract format of the intended publication; typically includes background, purpose, methods, results and conclusions	2
Problem formulation	#3	Description and signifcance of the problem / phenomenon studied: review of relevant theory and empirical work; problem statement	4 and 5
Purpose or research question	#4	Purpose of the study and specific objectives or questions	5
Qualitative approach and research paradigm	#5	Qualitative approach (e.g. ethnography, grounded theory, case study, phenomenolgy, narrative research) and guiding theory if appropriate; identifying the research paradigm (e.g. postpositivist, constructivist / interpretivist) is also recommended; rationale. The	7

For peer review only

		rationale should briefly discuss the justification for choosing that theory, approach, method or technique rather than other options available; the assumptions and limitations implicit in those choices and how those choices influence study conclusions and transferability. As appropriate the rationale for several items might be discussed together.	
Researcher characteristics and reflexivity	#6	Researchers' characteristics that may influence the research, including personal attributes, qualifications / experience, relationship with participants, assumptions and / or presuppositions; potential or actual interaction between researchers' characteristics and the research questions, approach, methods, results and / or transferability	19
Context	#7	Setting / site and salient contextual factors; rationale	7
Sampling strategy	#8	How and why research participants, documents, or events were selected; criteria for deciding when no further sampling was necessary (e.g. sampling saturation); rationale	7
Ethical issues pertaining to human subjects	#9	Documentation of approval by an appropriate ethics review board and participant consent, or explanation for lack thereof; other confidentiality and data security issues	6
Data collection methods	#10	Types of data collected; details of data collection procedures including (as appropriate) start and stop dates of data collection and analysis, iterative process, triangulation of sources / methods, and modification of procedures in response to evolving study findings; rationale	7
Data collection instruments and technologies	#11	Description of instruments (e.g. interview guides, questionnaires) and devices (e.g. audio recorders) used for data collection; if / how the instruments(s) changed over the course of the study	8
Units of study	#12	Number and relevant characteristics of participants, documents, or events included in the study; level of participation (could be reported in results)	See note 1
Data processing	#13	Methods for processing data prior to and during analysis, including transcription, data entry, data management and security, verification of data integrity, data coding, and anonymisation / deidentification of excerpts	8
Data analysis	#14	Process by which inferences, themes, etc. were identified and	8

For peer review only

		developed, including the researchers involved in data analysis; usually references a specific paradigm or approach; rationale	
Techniques to enhance trustworthiness	#15	Techniques to enhance trustworthiness and credibility of data analysis (e.g. member checking, audit trail, triangulation); rationale	8
Syntheses and interpretation	#16	Main findings (e.g. interpretations, inferences, and themes); might include development of a theory or model, or integration with prior research or theory	8-16
Links to empirical data	#17	Evidence (e.g. quotes, field notes, text excerpts, photographs) to substantiate analytic findings	See note 2
Intergration with prior work, implications, transferability and contribution(s) to the field	#18	Short summary of main findings; explanation of how findings and conclusions connect to, support, elaborate on, or challenge conclusions of earlier scholarship; discussion of scope of application / generalizability; identification of unique contributions(s) to scholarship in a discipline or field	16
Limitations	#19	Trustworthiness and limitations of findings	19
Conflicts of interest	#20	Potential sources of influence of perceived influence on study conduct and conclusions; how these were managed	4
Funding	#21	Sources of funding and other support; role of funders in data collection, interpretation and reporting	3

Author notes

1. 7,8,9 and especially 10

2. 8-16 and appendix

The SRQR checklist is distributed with permission of Wolters Kluwer © 2014 by the Association of American Medical Colleges. This checklist was completed on 26. March 2019 using https://www.goodreports.org/, a tool made by the EQUATOR Network in collaboration with Penelope.ai

For peer review only

BMJ Open

Mhealth – Providing a Mindfulness App for women with chronic pelvic pain in gynaecology outpatient clinics: Qualitative data analysis of user experience and lessons learnt

Journal:	*BMJ Open*
Manuscript ID	bmjopen-2019-030711.R1
Article Type:	Original research
Date Submitted by the Author:	26-Sep-2019
Complete List of Authors:	Ball, ELizabeth; Queen Mary University of London - Whitechapel Campus, Yvonne Carter Building<br>Newton, Sian; QMUL<br>Rohricht, Frank ; East London NHS Foundation Trust<br>Steed, Liz; QMUL<br>Birch, Judy<br>Dodds, Julie; QMUL, Women'shealth Research Unit<br>Cantalapiedra Calvete, Clara; QMUL, Women's Health Research Unit<br>Taylor, Stephanie; QMUL, Center for Primary Care and Population Health<br>Rivas, Carol; University College London
**Primary Subject Heading**:	Obstetrics and gynaecology
Secondary Subject Heading:	Health informatics
Keywords:	Chronic pelvic pain, mHealth, mindfulness, patient engagement, health app, feasibility study

SCHOLARONE™
Manuscripts

I, the Submitting Author has the right to grant and does grant on behalf of all authors of the Work (as defined in the below author licence), an exclusive licence and/or a non-exclusive licence for contributions from authors who are: i) UK Crown employees; ii) where BMJ has agreed a CC-BY licence shall apply, and/or iii) in accordance with the terms applicable for US Federal Government officers or employees acting as part of their official duties; on a worldwide, perpetual, irrevocable, royalty-free basis to BMJ Publishing Group Ltd ("BMJ") its licensees and where the relevant Journal is co-owned by BMJ to the co-owners of the Journal, to publish the Work in this journal and any other BMJ products and to exploit all rights, as set out in our licence.

The Submitting Author accepts and understands that any supply made under these terms is made by BMJ to the Submitting Author unless you are acting as an employee on behalf of your employer or a postgraduate student of an affiliated institution which is paying any applicable article publishing charge ("APC") for Open Access articles. Where the Submitting Author wishes to make the Work available on an Open Access basis (and intends to pay the relevant APC), the terms of reuse of such Open Access shall be governed by a Creative Commons licence – details of these licences and which Creative Commons licence will apply to this Work are set out in our licence referred to above.

Other than as permitted in any relevant BMJ Author's Self Archiving Policies, I confirm this Work has not been accepted for publication elsewhere, is not being considered for publication elsewhere and does not duplicate material already published. I confirm all authors consent to publication of this Work and authorise the granting of this licence.

For peer review only

Mhealth – Providing a Mindfulness App for women with chronic pelvic pain in gynaecology outpatient clinics: Qualitative data analysis of user experience and lessons learnt

Ball, Elizabeth
Department of Obstetrics and Gynaecology, Barts Health NHS Trust, United Kingdom, Women's Health Research Unit, Barts and the London School of Medicine and Dentistry, Queen Mary University of London, United Kingdom, Centre for Maternal and child Health Research, City University London

Newton, Sian
Centre for Primary Care and Population Health, Queen Mary University of London, United Kingdom

Rohricht, Frank
East London NHS Foundation Trust, United Kingdom

Steed, Liz
Centre for Primary Care and Population Health, Queen Mary University of London, United Kingdom

Birch, Judy
Pelvic Pain Support Network, UK

Dodds, Julie
Women's Health Research Unit, Barts and the London School of Medicine and Dentistry, Queen Mary University of London, United Kingdom

Cantalapiedra Calvete, Clara
Department of Obstetrics and Gynaecology, Barts Health NHS Trust, United Kingdom

Taylor, Stephanie JC
Centre for Primary Care and Population Health, Queen Mary University of London, United Kingdom

Rivas, Carol
Department of Social Science, University College London, United Kingdom

Corresponding author

Carol Rivas, Department of Social Science, University College London, United Kingdom
E-mail: c.rivas@ucl.ac.uk

For peer review only

Abstract

Objective

To determine whether a pre-existing smartphone App to teach Mindfulness Meditation is acceptable to women with chronic pelvic pain (CPP) and can be integrated into clinical practice within NHS CPP pathways. To inform the design of a potential randomised clinical trial.

Design

A pre-study patient and public involvement (PPI) group to collect feedback on the acceptability of the existing app and study design was followed by a three-arm randomised feasibility trial. In addition, we undertook interviews and focus groups with patients and staff to explore app usability and acceptability. We also obtained participant comments on the research process, such as acceptability of the study questionnaires.

Setting

Two gynaecology clinics within Barts Health NHS, London, UK.

Participants

Patients with CPP lasting $\geq$6 months with access to smartphone or PC and understanding of basic English.

Intervention

The intervention was mindfulness meditation content plus additional pain module delivered by smartphone app, active controls received muscle relaxation content by the same app. Passive (waiting list) controls received usual care.

Main outcome measures

Themes on user feedback, app usability and integration and reasons for using/not using the app.

Results

App use was low in both active groups.

Patients in the pre-study PPI group, all volunteers, were enthusiastic about the app (convenience, content, portability, flexibility, ease of use). Women contributing to the

For peer review only

interview or focus group data (n=14), from a ‘real world’ clinic, (some not regular app users) were less positive, citing as barriers lack of opportunities/motivation to use the app, and lack of familiarity and capabilities with technology. Staff (n=7) were concerned about the potential need for extra support for patients and staff and considered the app needed organisational backing and peer acceptance.

Conclusion

The opinions of pre-study PPI volunteers meeting in their private time may not represent those of patients recruited at a routine clinic appointment.

It may be more successful to co-design/co-develop an app with typical users than to adapt existing apps for use in real-world clinical populations.

Trial registration and funding

The trial (ISRCTN 10925965) was funded by the UK National Institute of Health Research, Research for Patient Benefit programme (RfPB PB-PG-1013-32025).

Keywords

Chronic pelvic pain, mHealth, mindfulness, Headspace, PPI, patient engagement, feasibility study, health app

For peer review only - http://bmjopen.bmj.com/site/about/guidelines.xhtml

Article Summary; 'Strengths and limitations of this study' (relating to methods)

- The study was designed with the help of a study design group of CPP patients
- Recruitment to the study was good
- The qualitative analysis suggests low acceptability which has implications for evaluations of efficacy
- In depth interviews with participants give learning points for future apps, indicating apps co-designed with patients may be preferable for use within health care than modified commercial apps
- Participant feedback and recruitment showed that the research process per se was successful and can be used in the future.

The original protocol for the study has been published separately. The UK National Institute of Health Research supported this work (RfPB PB-PG-1013-32025). There are no competing interests.

Introduction

Smartphone health apps, as one form of mhealth (1), are popular in the UK, our study setting. With more than two-thirds of the UK population using smartphones (2,3,4), health apps are one of the fastest growing app categories, thus numbers of users are still increasing (5). Currently these apps are usually developed either by researchers or (in the majority) by commercial companies, without collaboration between these groups (6,7). The lack of interaction between researchers and commercial developers in the field of pain-related apps has led to a situation where commercially available apps have not been scientifically validated and apps that have been developed from research projects are not commercially available (8).

For peer review only - http://bmjopen.bmj.com/site/about/guidelines.xhtml

We were interested in using an app to support women with chronic pelvic pain (CPP) in a clinical setting, where validation of an intervention is important to ensure best care. CPP is defined as a subjective physical and emotional experience of pain in the pelvic area that has been present for at least six months that may or may not have an identifiable pathology (9). CPP affects up to 24% of women worldwide (10) and accounts for 20% of gynaecological clinic referrals. (11,12) It has considerable impact on patients' quality of life, including their mental health and their income (13) due to loss of working days and diminished work capacity. Annual costs to the NHS have been estimated at approximately £326 million (14). For endometriosis alone, which is just one cause of CPP, a European study of over 900 women showed average annual total costs per woman of €9579. Costs of productivity loss of €6298 were double the health care costs of €3113 per woman. The latter were due to surgery (29%), monitoring tests (19%) and hospitalization (18%) and physician visits (16%) (15).

Despite costly interventions, CPP is often resistant to surgical and medical treatment and appears to respond better to a multimodal, holistic approach, (16) with a focus on coping strategies. A systematic review of randomised controlled trials (RCTs) by authors has identified mindfulness meditation (MM) as an effective coping strategy in other chronic pain conditions (17). In addition, evidence from uncontrolled trials suggests positive effects of MM for CPP, such as an increased ability to control pain, improvements in mental health, emotional well-being, work and family life and social functioning (18,19), but these have never been examined in an RCT.

We therefore chose to evaluate MM delivered via an app to women with CPP as our intervention. CPP is especially common in younger women, who may be categorised as digital natives, making an app-based intervention particularly appropriate in this group.

In MM the aim is to keep focussed on one's own breathing. Whenever attention wanders to intrusive thoughts and feelings these are simply taken notice of in a neutral way, that is,

For peer review only - http://bmjopen.bmj.com/site/about/guidelines.xhtml

without elaboration or judgements or consideration of action. They are then let go as attention is returned to the breath. The idea is to bring awareness back to the here and now whenever worries and troubles intrude into thoughts.(20)

Generally two main complementary approaches have been used for MM: 1) exercises focusing one's attention to the present moment, and 2) monitoring of experiences in the present moment. While systematic reviews show that MM may have positive effects on depression, quality of life and pain symptoms in patients with chronic pain (17,21,22) and apps with such a focus on chronic disease have been shown to be beneficial in various conditions (23) none of the reviewed papers included meditation delivered via mobile phone apps or in women with CPP.

Evaluation of an existing app is often appropriate (24) and is both quicker and more cost-effective than designing an app from scratch. We chose to evaluate an existing commercial app platform that teaches mindfulness by guided meditation (Headspace ®), with a ten day basic meditation module followed by a pain module specifically designed for the MEMPHIS (Mindfulness meditation using a smart-phone application for women with chronic pelvic pain) study. The Headspace app was publicly nominated favourite health app of 2013,(25) has a 5 star user rating in the Apple™ app shop and has scored top in a systematic review of 23 mindfulness apps using the Mobile Application Rating Scale (visual aesthetics, engagement, functionality or information quality) (26). Headspace had reportedly seen over 15 million downloads up to mid-2018 when our study began.(27) To our knowledge the Headspace app in its original or modified form has not been assessed in any other pain conditions.

We undertook a feasibility study (28) to assess whether or not to proceed with a full randomised controlled trial of the modified Headspace meditation app for women with CPP. In the current paper we report on the qualitative interview and focus group data from this study; the protocol and quantitative results have been published/ submitted (29,30). Our aim

For peer review only - http://bmjopen.bmj.com/site/about/guidelines.xhtml

in this analysis was to determine whether a pre-existing smartphone app to teach MM is acceptable to women with CPP and can be integrated into clinical practice within NHS CPP pathways.

Methods

The MEMPHIS trial was a three-arm parallel randomised feasibility trial approved by Camden and Kings Cross Research Ethics Committee in 2016 (15/LO/1967). The quantitative analysis is published in a companion paper (29); the present paper reports the qualitative analysis.

Objectives for the qualitative part of this study, using interviews and focus groups were to consider:

1) The acceptability, use and usability of the app in the intended service user population and for health care professionals (doctors, health care assistants, clinical and research nurses).
2) The feasibility of integrating such an app into existing healthcare pathways.
3) Feedback on the research process.

We follow the ISO 9241-11 (https://www.iso.org/obp/ui/#iso:std:iso:9241:-11:ed-2:v1:en) concept of technology usability (user friendliness) as the extent to which the app could be satisfactorily used by participants to meditate. By acceptability we mean whether participants could see a reason for using the app when given in clinic, and would be happy to use it for meditation.

Outcomes

The outcomes of this analysis were inductively derived descriptive themes on acceptability, use and usability of the app and feasibility of integrating it into existing pathways.

Intervention Procedures

For peer review only - http://bmjopen.bmj.com/site/about/guidelines.xhtml

Women in the mindfulness meditation group received access to a 60-day progressive mindfulness meditation course delivered via the Headspace app. The first 10 days of the course taught basics of mindfulness meditation. Following this, participants were able to access the module on meditation which was targeted at for chronic pain. This module had been specifically made for this study. Session length was 10 minutes for the first 10 days, 15 min up to day 20 and 20 min up to day 60. The active control group received access to a series of muscle relaxation sessions. These sessions were identical every day, except that their duration increased to mirror the increasing duration of the meditation content being listened to by the intervention group. Usage data are reported elsewhere (30).

Patient and Public Involvement

We held a Patient and Public Involvement (PPI) group workshop before the study to discuss acceptability of the Headspace app and help us design our study. Women attending the Royal London Hospital CPP clinic were invited to volunteer for a week of using the unmodified (normal commercially available) Headspace app (which did not have the pain module at the time we undertook our pre study workshop) and then feed back on their experiences with the app in an evening discussion group. Women were not involved in the design of the modified app. The focus for the PPI group was on the use of the generic MM app.

Two patient representatives provided support from the study design stage through recruitment to the interpretation of the results and regularly attended Trial Management Group meetings.

Study recruitment and eligibility

The trial recruited at two outpatient gynaecology clinics within Barts Health NHS trust in two separate deprived areas of inner East London. Female patients with new or follow-up

For peer review only - http://bmjopen.bmj.com/site/about/guidelines.xhtml

gynaecology appointments were assessed for eligibility by a researcher in clinic, having been posted a Patient Information Sheet. Women were eligible if they had been suffering with CPP for 6 months or more and had at least a basic understanding of the English language, sufficient to follow instructions, as assessed during discussion about the study for informed consent; no women were excluded on this basis. Women were excluded if they did not meet these criteria or they did not have access to a smartphone or personal computer or were currently using the Headspace app (there were very few of the latter, according to the impression of the recruiting nurses). All patients gave full and informed consent to be randomised and data were collected through all stages of the study. All healthcare professionals and research nurses involved in the two clinics were also invited to take part in the feasibility study as the only eligibility criterion for staff. Full enrolment data are provided in Forbes et al (30). A key difference of these patients from those in the PPI group was that their focus was on managing their pain, with the app given explicitly as part of their clinic management support.

For the study of quantitative data, 90 patients were allocated randomly in a 1:1:1 ratio to the mindfulness meditation app, a muscle relaxation app active control or the usual care arm (for full details see (29)). Patients in the two active arms were asked to download the modified app in the clinic with support from a research staff member and were sent a questionnaire about app usability, an analysis of which is reported in a companion paper.(30) We used data from the app usability questionnaire to inform topic guides for the qualitative part of the study. This outlined key usability issues that had been uncovered, to guide our semi-structured interviews and focus groups with patients and staff.

All women in the intervention and active control arms were eligible for the qualitative component of the study, and all staff participating in the study.

Within-study interviews and focus groups

For peer review only - http://bmjopen.bmj.com/site/about/guidelines.xhtml

All patients in the intervention and active control arms were invited to one of two focus groups at their own study site after the 6-month follow up. We offered telephone interviews as an alternative. Patients were asked to ‘walk through’ the app with researchers, articulating their thought processes while they did so and commenting on its different specific features, (31). Walkthroughs are often used in developing technologies such as mhealth. This helped to identify app usability issues or barriers to use of the app from the users’ points of view without the need for technical discussions. Results for the walkthrough, showing comments on different features specific to the usability of the intervention app used in our study are shown in Appendix 1; walkthroughs were undertaken by two patients. Patients also discussed with us their experiences around app usability and acceptability.

Staff were invited to attend a staff focus group overseen by the patient representative and facilitated by a researcher. In addition to considering app usability and acceptability, members of the staff focus group (consultants, health care assistants, clinical and research nurses and a representative from the pelvic pain support network were eligible) were asked about the ease of integration into existing NHS pathways. Part of the staff discussion was free flowing with open-ended questions, which gave us patient-focussed information on app acceptability, and part was structured using questions developed from the Normalisation Process Theory (NPT) toolkit in the way recommended by the NPT developers (32). For example, we asked whether staff could see a purpose to the app in clinical practice, as adding something different, which corresponds to the NPT toolkit question ‘Participants distinguish the intervention from current ways of working’. Since this was a semi-structured approach questions were not rigidly worded. This helped us to consider the feasibility of integration of the app into practice. NPT is a theory of implementation practices that was initially developed for consideration of technology implementation and is in common use (32).

For peer review only - http://bmjopen.bmj.com/site/about/guidelines.xhtml

All data were audio-recorded at point of collection and transcribed, with personal identifying data removed from transcripts. Raw data were stored in a Primary Care Clinical Trial Unit database to clinical trial standards.

Analysis

Analysis of within-study focus groups and interviews was carried out blinded as to which study app was used, and deployed the immersion-crystallisation method (33). Thus, the lead qualitative researcher immersed herself in the data, reading transcripts carefully, then writing down articulated or crystallised patterns or themes that related to the aims and research questions of the study. These were discussed with another researcher from the team, and themes modified as appropriate. This process was repeated until all the data had been examined and all patterns that had been noticed were articulated, discussed and substantiated with exemplar extracts. This approach was considered appropriate since we had a small dataset and we were not aiming to develop conceptual themes but rather to inform the design and development of a randomised controlled trial for the modified app. We used the SRQR checklist when writing our report (34).

Results

We screened 488 women between May and September 2016 for their eligibility to participate in the study. After exclusions, 90 women gave full consent to participate and were randomised to the intervention arm (31 women), the active control arm (30 women) or the usual care arm (29 women).

Demographics

Women in the main feasibility trial (30) were aged a mean of 35 years, 66% were employed and overall approximately 50% had stayed in full time education until at least the age of 20 though the proportion was least, at 36.7% in the intervention arm. Overall 44% were of white

For peer review only - http://bmjopen.bmj.com/site/about/guidelines.xhtml

ethnicity though the proportion was lowest in the intervention arm (35.7%) and highest in the usual care arm (53.6%). The second most common self-reported ethnic groups were 'Southern Asian' and 'Black'. Women in the intervention arm were most likely to have experienced CPP for 3-5 years (40.3% of this group), women in the usual care group for over 10 years (42.9% of this group). More women had pain for longer than two years in the intervention arm than in either of the other two arms. All women reported a high pain intensity, with means of 6.8 to 6.9 in the previous week (on a scale from 1-10) (30). These and other demographic data are reported in more detail in Forbes *et al*. (30) Our qualitative sample was taken from the two active arms and was comprised of 16% of trial participants and 23% of those eligible for the qualitative study. We did not record separate demographic data for the women in this smaller sample.

For peer review only

Pre-study PPI group

The ten women in the pre-study PPI group were self-selected local women who were familiar with using apps and focused on app use per se. They anticipated no technical issues even in women who were not used to apps. They considered that the Headspace app would be successfully adopted by patients taking part in the study, given that a smartphone, like CPP, is ‘always with you’. They praised the flexibility of the app, welcomed its portability and were unanimous in saying it was easy to slip off for 10 minutes when at work to use it. As a result, they found they could use it at times when they most needed pain relief as well as to prevent pain and found the app helpful in relieving pain and stress. The group reported being able to meditate without the app, once they had tried it with the app; however, they still preferred to use the app because they found the voice soothing.

App usage in the study

Patient usage of the app was less than expected from our pre-study PPI group discussions. Only 36% of meditation app patients and 46% of the active control patients used the app at least once. (30)

Thematic analysis of within-study data

Qualitative data were obtained from 14 study patients; 12 preferred a telephone interview, two attended face-to-face interviews, one participant at the University attached to one of the recruiting clinics and one participant at the other recruiting hospital. Patients chose not to attend focus groups. Four of the patients were from the active control arm and 10 from the intervention arm. The two women we met face to face had both used the intervention and neither had progressed beyond the training stage, something that we cannot discount for other participants and which may help to explain reports of lack of effect on pain. Seven people attended the staff focus group: two recruiting nurses, three clinic nurses one consultant and a representative from the pelvic pain support network.

For peer review only - http://bmjopen.bmj.com/site/about/guidelines.xhtml

The qualitative analysis revealed three main themes from all within-study interviews and focus groups combined regarding usability, and four subthemes:

1. Familiarity and capabilities with app technology
2. Motivations to use the app
   - Perceived benefits
   - Relation to other therapies
3. Opportunities to use the app:
   - Technology issues getting in the way
   - Life getting in the way

These are explored below. As the PPI group data were not research data we did not analyse them for themes.

While we initially combined active control and intervention groups in our analysis, we then looked for instances where there was a difference between these two groups. Only where we found this difference in any theme or statement have we specified which group women belonged to.

Familiarity and capabilities with app technology

Around half of the patients were sufficiently familiar with technology and apps to be comfortable using the study apps. However, six participants (all using the intervention, which was more complex than the active control) reported difficulty because they were *"not very good at technology*" (patient 1002, intervention), or were unsure how to get started or use the app effectively without help.

> *I am not good with technical some things that is why the problems I had arisen, right okay. So I consulted with my daughter and she helped me work it out… so I don't try everything. (patient 1002, intervention)*

One further patient *(1001, intervention)* was not used to technical app language; 'help' suggested emotional support to her, for example. Two more (one intervention, one active

For peer review only - http://bmjopen.bmj.com/site/about/guidelines.xhtml

control) changed handsets and therefore did not continue with the app. In all cases these technical difficulties appeared to lead to abandoning of the app or restricted use of its functionality.

Five patients having technical problems suggested possible solutions such as a ‘class’ or group for first time users, a YouTube orientation video, or a pictorial leaflet. This might include an introduction to meditation and mindfulness as well as the app itself. One woman commented: *“If your market is targeting people who are not using apps then you are going to have to get together and find ways to do this”,* (patient 1041, intervention); she also suggested we could get ideas from other apps on the market in this regard.

Given their experiences in the study, staff were concerned about additional staff time needed to support women in using the app. This would sit in tension with one of the original rationales behind choosing an app as the mode of delivery, which was to increase the effective use of staff contact time with patients. Language barriers might compound problems.

Motivations to use the app

Staff, though unaware of the low sustained app use in the study, felt it would be common sense to hold occasional motivating meetings with patients if the intention was for them to use the app long term. The patient data suggested the main motivators or lack of motivation for using the app in our sample, which could be drawn on in such meetings, and which we now consider.

Perceived benefits

Three intervention arm patients said they only entered the trial to help others through research but were already using alternative forms of pain control. They explained that this meant they were not motivated to actually use the app, perceiving the relative benefit to be

For peer review only - http://bmjopen.bmj.com/site/about/guidelines.xhtml

small. The failure of such altruism to extend to using the app is a recognised phenomenon in clinical trials and has been called 'weak altruism'. (35) Thus, only one of these three patients persevered. Even though she was one of the women who experienced difficulties with the technology, she explained, "*with something that is as soul destroying as the pain, it is important to help others off the back of other people's misfortune as it were"* (1036, intervention). However, she wondered how relevant her data were:

I took steps to improve my situation from a weight loss perspective as well and I've lost a lot of weight which has significantly helped not 100% but it is has significantly helped so I felt a bit fraudulent the last time filling in the forms because , so everything had improved so much so from the medical study perspective it was more about the weight loss than the app. So I felt a bit bad that I was still taking part. (patient 1036)

There was no clear pattern regarding the impact of current pain on app use by patients. Six said they used it regardless of pain intensity – sometimes developing a daily routine - while four only used it when in severe pain or expecting to be (e.g. during menstruation). This cyclical or intermittent use in some patients – which was irrespective of study arm - should be considered when looking at our main study outcomes.(30) The Headspace app requires regular use to learn and benefit from psychological techniques. To address this, healthcare professional alerts have been effective in other studies, (36) whilst Headspace only has a reminder function that the user can set. This was often not sufficient, as one patient said even with this feature, "*To be quite honest I used it a couple of times and then forgot. And then I [remembered it and] used it more frequently." (patient 1036, intervention)*

One patient said medication was not working but the app did, though she was not sure whether this was '*mind over matter'* (1065, intervention), which was her term for a placebo effect. Three others said it did not reduce their pain; all three were using the intervention app. The remaining patients considered other benefits were good reason for using the app even when they did not feel that it reduced pain intensity. Alternative or unanticipated benefits were not formally measured or taken into account in the study's effectiveness

For peer review only - http://bmjopen.bmj.com/site/about/guidelines.xhtml

outcomes (30). For example, ten patients valued the way the app helped them to relax or de-stress or focus and re-assess their life; three of these specifically said they used it to induce a relaxed state to get to sleep. Notably the active control was a relaxation app; however this benefit was also reported by many women in the intervention arm. One participant (active control) said she did not like the focus on pain per se as her condition impacted on various areas of her life. Even when the intervention app was positively received, women might stop using it because it was too powerful, and they had gained the change they wanted:

I think it was day 3, I could see the change that was happening, I was able to speak up for myself ……I can't explain it, even now I am getting emotional… it's just a lack of focus, I just needed direction. To try and put It into words. To me it meant so much that I have gone back to church… I use it outside of the app now I have got from it what was missing, so it's done something to me and for me which is very positive, and I may try it to lose weight but those positive vibes are still there. I can't go back to it because I did not want to go any further because what I got at the time helped me to focus, to change my way of thinking. I used it for about two or three weeks. (patient 1001, intervention)

Three intervention app patients found the app put them more in tune with their bodies and their breathing, (two of these were among those who also found the app de-stressing) while another found yoga better for that. Six patients, like pre-study PPI group members, also learned to use techniques from the app to alter their stress patterns without the app, having tried it, for example in traffic or by sitting down and taking time out or for general relaxation.

<u>Relation to other therapies</u>

Two patients (both active control) preferred 'pure' meditation, another considered the app to be "*very much about meditation*" (1041, intervention), which is in keeping with the arms they were in. An alternative therapy practitioner and two further patients reported that they preferred yoga. One (active control) said this was because it focussed on each part of the body in turn.

For peer review only - http://bmjopen.bmj.com/site/about/guidelines.xhtml

Three intervention app patients thought the app was useful as an adjunct to other methods rather than a replacement for them, for example physical interventions such as Pilates, or listening to classical music.

Opportunities to use the app

Technology issues getting in the way

Staff pointed out that not all patients had smartphones (not appreciating that PCs/tablets were alternatives allowed in this study). Some patients lacked the storage space to load the app on their phones. There were also issues with Wi-Fi connectivity when staff tried to help the patients load the app within the hospital sites. Possible solutions that staff suggested were to lend patients phones and to have group upload sessions in a location with good Wi-Fi signal – though they acknowledged the resource implications.

Life getting in the way

Seven patients revealed they preferred to use the app in the evenings because of other life commitments. This meant they did not always use it as a direct response to pain, reducing its potential for contemporaneous effect. One patient who used it in response to pain but only used it once or twice blamed this on having no spare time because of juggling work and children (active control); however, another patient (intervention) managed despite such commitments the fact that she was in the intervention arm may have played a role.

Barriers to integration for staff

Staff believed that the biggest barrier to clinical adoption of the app was a possible lack of support from the host organisation. It might also be hard to integrate the app within existing professional work practices if the staff in the position of offering the app to patients failed to see its relative advantage over other interventions. Collection of feedback on the app's effectiveness would be necessary for staff to support sustained use. It was felt that staff would need training on how to introduce the app to women in practice, and that complexity

For peer review only - http://bmjopen.bmj.com/site/about/guidelines.xhtml

and high staff turnover could impede sustained use. An app was also seen as impersonal compared with face-to-face contact, which was more favoured by staff.

Participant comments on the research process

The study questionnaires that were used for the main quantitative outcome measures (30) were acceptable to patients except for some discomfort with a question about sex, which patients considered a delicate question that was missing a 'no sex' option. Most preferred a paper form reflecting their lack of affinity with technology. There were no indications that the study design or study processes had contributed to the participants' lack of engagement with the apps – with a caveat around support with the technology as mentioned above - though we did not systematically consider this. A full summary of patient comments on the study design and procedures is given in Appendix 1.

Discussion

Our study adds to the limited evidence on mHealth app user behaviour and experience (36,37). The pre-study PPI group (young women, of a generation who were familiar with using apps and who were asked to focus on the study design use of the app) liked the idea of delivering the intervention via an app, praising the contemporary design and flexibility. Hence we expected a similar positive attitude from trial participants, who were of a comparable age, and we assumed would be keen on using apps. Participant feedback revealed that this assumption was too simplistic.

Using our qualitative data, we were able to explore reasons for low app usage that had been recorded in our feasibility study.(30) Our thematic analysis suggests that the low app use in the trial occurred because many patients were not familiar with apps in general or lacked capabilities with technology. This was particularly true for the more complex intervention app. The other themes we report did not differ between groups (although the three cases of weak altruism' all occurred in the intervention arm) which suggests more generic issues that

For peer review only - http://bmjopen.bmj.com/site/about/guidelines.xhtml

can be transferable to other app use situations. For example, women stated limited motivation to use the app because of a lack of perceived benefit, or a lack of opportunity to use the app due to Wi-Fi issues or due to other commitments.

Similar findings were reported by Laurie *et al*.(38) who interviewed 16 healthy city-dwelling participants (25-38 years) about their user behaviour before and after 30-40 days of Headspace app exposure. Like us, they reported barriers of busy lives, failure to establish a routine and a lack of perceived benefit; all users in their study tried the app at least once hoping it could deliver a quick fix but were disappointed if this did not happen. In our study many patients failed to perceive a benefit from using the app. Hence excuses stating other commitments may mask a deeper lack of motivation linked to perceptions of benefits.(39)

The advantages and disadvantages of using the app stand-alone were also illustrated by our data. Some suggestions made by participants to improve usage, such as more guidance at the start, seem obvious in hindsight. But they had not been considered because of the feedback from the pre-study PPI group and the commercial success of Headspace. The use of community contacts may be a helpful alternative.(40) Social support can create a community of practice, help to clarify expectations,(41) and improve health outcomes (as shown for example in internet based psychological treatment for depression (42).

The data suggest that for successful app use we need to understand what motivates individuals with clinical need to use the app for clinical reasons (which our PPI group did not focus on) and target this, for example by setting appropriate expectations. Incentivisation might also improve motivation. This could be achieved through app gamification (7), or encouragement through integration with patient-clinician face-to-face encounters, which was lacking in our study since the app was used stand-alone. The present study provided extensive initial technical support but no coaching and incentivising, in keeping with the protocol. Future app studies should take this into account. Participants in our study may

For peer review only - http://bmjopen.bmj.com/site/about/guidelines.xhtml

have also have benefitted from training and support to improve their app use capabilities and guidance on how to create more opportunities for app use – such as through sharing experiences in clinic support groups. This is in keeping with the COM-B model of behaviour change (43) which our themes matched, though this was only realised after analysis. The COM-B model says that Capability, Opportunity and Motivation are key drivers of behaviour and has been used to develop a number of complex interventions including smartphone apps (e.g. 3).

Lack of engagement after recruitment, or good initial engagement but minimal or inconsistent use, have been reported in other studies (44,45), including in Headspace trials other than our own (46, 47). Settings were a university and a psychiatric inpatients clinic, both in the USA. Inconsistent app use was noted by Wen (48) in junior doctors who used self-guided Headspace. Morrison Wylde (49) compared face-to-face MM with headspace use in novice paediatric nurses. However, unlike our study there were no recorded dropouts/non-users and also no record of whether or how long the app was used for which is an important omission.

None of these studies included a qualitative component. Yet, each of the Headspace study groups were very different, and so will likely have differed in motivations, contexts for opportunity to use the app, and incentivisations (50). While these aspects were not considered in the other studies, our use of qualitative research has enabled us to explore these in more depth. Our findings suggest these are important considerations in any study of app use and therefore this study makes a contribution to the field. For example, some of the groups in other studies may have differed from ours in likelihood of using mhealth apps in the first place, and familiarity with technology. In-patients may have more time to use the app and more support – and may also have had specific barriers to app use, such as related to setting and to illness.

For peer review only

Patients in the qualitative part of our study tended not to use apps on a regular basis (or at least apps other than simple games), and in terms of our themes, also represented in the COM-B model, may be said to have few capabilities in technology use. They therefore do not represent the typical users of the Headspace app in a commercial setting. Accessing the app regularly requires energy, time and effort, but patients with CPP often suffer from fatigue and anxiety as co-morbidities, perhaps whilst having to juggle family life and work. Therefore, this may be seen as a challenging clinical population in which to trial an app. Further Headspace trials with diabetic (NCT03274362) and pain **(**NCT03495726**)** outpatients are underway.

Our study has also shown that clear consideration of unexpected benefits should be included in future studies and these can be informed by our finding that benefits for patients may be more diffuse than anticipated (e.g. app relieving stress rather than pain). Such benefits were found in the active control as well as the intervention arm and so it may be that they represent a placebo effect though the effect could equally be real. Our data also suggest that staff benefits may be less than anticipated, as participants sometimes needed a lot of support and scaffolding in technology use at least initially.

Young age, co-morbid anxiety and low educational attainment are predictors for dropping out of web-based interventions according to studies in the field of depression. (51, 52) This may be true despite regular phone support (52) though our participants all considered active motivational support from staff or app support groups would have improved app use. Our intervention arm participants were particularly likely to be young and with low educational attainment.

Our data suggest that it is important to involve real world end users in the agile design or development or modification of apps in close collaboration with researchers and commercial app developers.(7) Although the evaluation of existing apps has been recommended as a

For peer review only - http://bmjopen.bmj.com/site/about/guidelines.xhtml

cost-effective and rapid process, (24) our findings suggest that in actual clinical practice these may be problematic.

Strengths and weaknesses of the study and in relation to other studies

One strength of this study is that it creates much-needed evidence in the field of evaluating existing health apps in a clinic population (6,8,24) and recording user experience. This provides us with lessons to be learned.

Researchers conducting interviews and focus groups were: a senior mixed methods medical sociology researcher, a recruiting nurse, a representative from the pelvic pain support network and an experienced health psychologist. Findings were similar across the data and the different backgrounds of the researchers therefore does not appear to have influenced findings. The main analysis was undertaken by the medical sociologist and so the concordance with the COM-B model is not due to background discipline bias.

We were able to recruit successfully, and we obtained valuable information from patients with CPP, who were recruited from a deprived urban area of the UK as typical local clinical patients.

However, we report a marked discrepancy between the attitudes of the pre-study PPI group of volunteer patients from the local area, who actively put themselves forward for a 7-day trial of the app, and the participants asked to take part when they attended clinics. The opinions of pre-study PPI volunteers meeting in their private time may not be representative of the opinions of patients recruited at a routine clinic appointment. Women in the PPI group were used to using apps, which had led them to be interested in the study in the first place. Whereas women in the PPI group had all trialled the app at home and work without support from us, many patients from clinic were unable to use their phone beyond calls, texts and photos. Moreover, most of the women we interviewed used the intervention app. We can only speculate as to why this is so but it does mean that concordances and divergences across the intervention and active control arm do need to be treated with circumspection.

For peer review only - http://bmjopen.bmj.com/site/about/guidelines.xhtml

To our knowledge the present observation on failure of PPI work to translate into practice in a trial has not been formally reported before, and is lacking from a recent comprehensive systematic review.(53) PPI involvement is a stipulated requirement when applying for some funding, and the present research findings should be taken into account when drafting guidelines for future PPI involvement in study planning. PPI groups are able to provide significant help and advice in any study but our findings shows the value of adding agile co-development as a requirement for app intervention development as likely to provide a more effective intervention than one informed by PPI alone. Moreover, there is a difference between app use for active clinical management (as with our study participants), and consideration of the potential for app use for this (as with our PPI group).

Implications for clinicians and policymakers

Given the patchy use of the app and the way that some participants did not manage to unlock its full functionality, and an indication of diffusion of benefit, more work is needed to see whether the app reduces pain per se. This study is a good example of the need to move away from 'one size fits all' behavioural interventions. Future studies should do more work on implementation before doing an effectiveness trial. This will enable researchers to be more nuanced about saying who the app is effective for, if at all.

Strategies to involve busy, less motivated, and less technologically experienced individuals in PPI and lay app design groups need to be further developed. These groups should include considerable scaffolding, which we have shown extends to study involvement by patients. More care is also needed to obtain PPI input that is representative of the target group, taking into account their capabilities, opportunities and motivational aspects. It may be useful to give the PPI group a small condition management task that emulates what trial participants will be required to do. Moreover, we can confirm a recent review suggesting that health apps should be co- designed with users (7), rather than presenting them with a

For peer review only - http://bmjopen.bmj.com/site/about/guidelines.xhtml

pre-existing app. These implications for our study are also generalisable to other technology studies.

Research in context

What is known

1. Chronic pelvic pain has a large impact on patients and the NHS and is difficult to treat.
2. Health outcomes are improved by psychological and lifestyle interventions but are often not addressed due to difficult access or service shortages.
3. Mindfulness meditation has not been investigated in chronic pelvic pain patients but randomised controlled trials show improved health outcomes in other chronic pain conditions.
4. Mhealth apps are increasingly popular.

What the study adds

1. A mindfulness meditation app may not necessarily be taken up by patients with CPP even when it is a commercial success in the general population. Considerable supportive scaffolding may be needed.
2. Use of existing commercial apps in actual clinical practice may be problematic compared with the agile development of apps with collaboration between researchers, clinicians, developers and end users.
3. Chronic Pain patients are interested in alternatives to drug or surgical treatments and further research is required in this area, including with MM, the benefits of which may extend beyond pain relief itself.

For peer review only - http://bmjopen.bmj.com/site/about/guidelines.xhtml

4. PPI groups may be more motivated to use an intervention than a real world clinical group as they are volunteers who are interested in the research topic. This may be particularly problematic for interventions that require considerable capability or motivation in use.

Author contributions

EB led the study as the CI. EB and CR were the main authors of the grant application for this study, and co-lead authors of the current paper. All other authors contributed to the study design and initial protocol, provided support throughout the trial and contributed towards the final paper. CR led on the PPI and CR and LS led the interview and focus group field work and analysis reported here. CR, LS, SN, CC, JD and JB were all involved in the field work.

Role of the funding source

The UK National Institute of Health Research, Research for Patient Benefit (RfPB No. PB-PG1013-32025) funded the MEMPHIS study. The funder had no role in the study design, in the collection, analysis, and interpretation of the data, in the writing of this report, or in the decision to submit the paper for publication. The first and last authors vouch for the integrity, completeness and accuracy of the data and analyses, and for the fidelity of this report to the protocol and statistical analysis plan. The views and opinions expressed herein are those of the authors and do not necessarily reflect those of the RfPB, NIHR, NHS or the Department of Health.

Competing interests

We have read and understood BMJ policy on declaration of interests.

Data sharing

The data are collected from a small number of people which could compromise their identity if shared with others. Therefore we are not making them available except under exceptional circumstances which will be determined by the custodian of the data (Elizabeth Ball) on an individual basis.

Transparency

For peer review only

The lead author confirms that the manuscript is an honest, accurate and transparent account of the study being reported; that no important aspects of the study have been omitted; and that any discrepancies from the study as planned and registered have been explained.

Acknowledgements

We would like to thank all the researchers, consultant obstetricians and gynaecologists and data assistants at each of the recruiting clinics for their hard work in promoting the study, recruiting participants and for data entry. Our thanks go to the Trial Steering Committee; Andrew Horne, Sohinee Bhattacharya, Christina Liossi, and Hulya Guzel for their constant support throughout the trial.

We thank the Pelvic Pain Support Network and Endometriosis UK for their promotion and guidance in developing the study design. We would also like to acknowledge the NIHR RfPB programme for their on-going support.

Lastly, thank you to Headspace Ltd for providing our participants with access to the Headspace platform, designing novel content for the study, and for their continuous support and advise throughout the study.

References

1. Ali EE, Chew L, Yap KY. Evolution and current status of mhealth research: a systematic review. BMJ Innovations 2016;2:33-40
2. Ofcom. The Communications Market Report. Ofcom: United Kingdom 2015. https://www.ofcom.org.uk/research-and-data/multi-sector-research/cmr/cmr15/uk [accessed 21 March 2019]
3. Kayyali R. Peletidi A. Ismail M, Hashim Z. Bandeira P, Bonnah J. Awareness and Use of mHealth Apps: A Study from England. Pharmacy 2017, 5, 33.
4. Sezgin E, Yildirim S, Özkan-Yildirim S, Sumuer E. Current and Emerging mHealth Technologies: Adoption, Implementation, and Use: Springer International Publishing; 2018 2018.

For peer review only - http://bmjopen.bmj.com/site/about/guidelines.xhtml

5. Lunden I. 6.1B Smartphone Users Globally By 2020, Overtaking Basic Phone Subscriptions https://techcrunch.com/2015/06/02/6-1b-smartphone-users-globally-by-2020-overtaking-basic-fixed-phone-subscriptions/?guccounter=12015 [cited 2018 28 August 2018].
6. Subhi Y, Bube SH, Rolskov Bojsen S, Skou Thomsen AS, Konge L. Expert Involvement and Adherence to Medical Evidence in Medical Mobile Phone Apps: A Systematic Review. JMIR Mhealth Uhealth. 2015;3(3):e79.
7. Edwards EA, Lumsden J, Rivas C, et al. Gamification for health promotion: systematic review of behaviour change techniques in smartphone apps. BMJ Open. 2016;6(10):e012447. doi:10.1136/bmjopen-2016-012447.
8. de la Vega R, Miro J. mHealth: a strategic field without a solid scientific soul. a systematic review of pain-related apps. PLoS One. 2014;9(7):e101312.
9. Engeler D, Baranowski AP, Borovicka J, et al. European Association of Urology. Guidelines on chronic pelvic pain. http://uroweb.org/wp-content/uploads/EAU-Guidelines-Chronic-Pelvic-Pain-2015.pdf. Accessed Sept 19, 2019.
10. Ahangari, A. Prevalence of chronic pelvic pain among women: an updated review. Pain Physician. 2014 Mar-Apr;17(2):E141-7.
11. Latthe, P., Latthe, M., Say, L., Gülmezoglu, M., & Khan, K. S. (2006). WHO systematic review of prevalence of chronic pelvic pain: a neglected reproductive health morbidity. BMC public health, 6, 177. doi:10.1186/1471-2458-6-177
12. Ayorinde AA, Macfarlane GJ, Saraswat L, Bhattacharya S. Chronic pelvic pain in women: an epidemiological perspective. Womens Health (Lond). 2015;11(6):851-64.
13. Howard F. The Role of Laparoscopy in Chronic Pelvic Pain: Promise and Pitfalls. Obstetrical & Gynecological Survey. 1993;48(6):357-87.
14. Zondervan KT, Yudkin PL, Vessey MP, Jenkinson CP, Dawes MG, Barlow DH, et al. Chronic pelvic pain in the community--symptoms, investigations, and diagnoses. Am J Obstet Gynecol. 2001;184(6):1149-55.

For peer review only - http://bmjopen.bmj.com/site/about/guidelines.xhtml

15. Curtis L. Unit Costs of Health and Social Care 2014 Personal Social Services Research Unit, University of Kent, Canterbury.: University of Kent, Canterbury; 2014 [Available from: https://www.pssru.ac.uk/project-pages/unit-costs/unit-costs-2014/.

16. Simoens S, Dunselman G, Dirksen C, The burden of endometriosis: costs and quality of life of women with endometriosis and treated in referral centres. Hum Reprod. 2012 May;27(5):1292-9. doi: 10.1093/humrep/des073. Epub 2012 Mar 14.

17. Peters AA, van Dorst E, Jellis B, van Zuuren E, Hermans J, Trimbos JB. A randomized clinical trial to compare two different approaches in women with chronic pelvic pain. Obstet Gynecol. 1991;77(5):740-4.

18. Ball EF, Nur Shafina Muhammad Sharizan E, Franklin G, Rogozinska E. Does mindfulness meditation improve chronic pain? A systematic review. Curr Opin Obstet Gynecol. 2017;29(6):359-66.

19. Kold M, Hansen T, Vedsted-Hansen H, Forman A. Mindfulness-based psychological intervention for coping with pain in endometriosis. Nordic Psychology. 2012;64(1):2-16.

20. Fox SD FE, Allen RH. Mindfulness meditation for women with chronic pelvic pain: a pilot study. J Reprod Med 2011;56(3-4):158-62.

21. Bishop SR. Mindfulness: A proposed operational definition. Clinical Psychology: Science and Practice; Autumn 2004; 11, 3; Health Module, pg. 230)

22. Plaza I, Demarzo MM, Herrera-Mercadal P, Garcia-Campayo J. Mindfulness-based mobile applications: literature review and analysis of current features. JMIR Mhealth Uhealth. 2013;1(2):e24.

23. Hilton L, Hempel S, Ewing BA, Apaydin E, Xenakis L, Newberry S, et al. Mindfulness Meditation for Chronic Pain: Systematic Review and Meta-analysis. Ann Behav Med. 2017;51(2):199-213.

24. Wang J,Wang Y, Wei C, Yao NA, Yuan A, Shan Y, Yuan C. Smartphone interventions for long-term health management of chronic diseases: an integrative

For peer review only - http://bmjopen.bmj.com/site/about/guidelines.xhtml

review. Telemed J E Health. 2014 Jun;20(6):570-83. doi: 10.1089/tmj.2013.0243. Epub 2014 May 1.

25. Boudreaux ED, Waring ME, Hayes RB, Sadasivam RS, Mullen S, Pagoto S. Evaluating and selecting mobile health apps: strategies for healthcare providers and healthcare organizations. Transl Behav Med. 2014;4(4):363-71.
26. My Health Apps [Available from: http://myhealthapps.net/app/details/127/Headspace-on-the-go] (Accessed 28 August 2018).
27. Mani M, Kavanagh DJ, Hides L, Stoyanov SR. Review and Evaluation of Mindfulness-Based iPhone Apps. JMIR Mhealth Uhealth. 2015;3(3):e82.
28. App Annie 2018 [Available from: https://www.appannie.com/en/] (Accessed 28 August 2018).
29. Ball E, Newton S, Kahan BC, Forbes G, Wright N, Cantalapiedra Calvete C, et al. Smartphone App Using Mindfulness Meditation for Women With Chronic Pelvic Pain (MEMPHIS): Protocol for a Randomized Feasibility Trial. JMIR Res Protoc. 2018;7(1):e8.
30. Forbes G, Newton S, Cantalapiedra C, Birch J, Dodds J, Steed E, Rivas C, Khan KS, Rohricht F, Taylor SJC, Kahan B, Ball E. A smartphone app using psychological approaches for women with chronic pelvic pain (MEMPHIS): a randomised feasibility trial. submitted
31. Gerhardt-Powals J. Cognitive engineering principles for enhancing human-computer performance. International Journal of Human-Computer Interaction. 1996;8(2):189-211.
32. Murray E, Treweek S, Pope C, MacFarlane A, Ballini L, Dowrick C, et al. Normalisation process theory: a framework for developing, evaluating and implementing complex interventions. BMC Med. 2010;8:63.
33. Borkan J. Immersion/Crystallization. In BF Crabtree and WL Miller (Eds) Doing Qualitative Research (2nd edition). Thousand Oaks, CA: Sage Publication; 1999. p. pp. 179-94.

For peer review only - http://bmjopen.bmj.com/site/about/guidelines.xhtml

34. O'Brien BC, Harris IB, Beckman TJ, Reed DA, Cook DA. Standards for reporting qualitative research: a synthesis of recommendations. Acad Med. 2014;89(9):1245-1251.

35. McCann, S. K., Campbell, M. K., & Entwistle, V. A. (2010). Reasons for participating in randomised controlled trials: conditional altruism and considerations for self. Trials, 11, 31. doi:10.1186/1745-6215-11-31

36. Palmer, M., Sutherland, J., Barnard, S., Wynne, A., Rezel, E., Doel, A., Grigsby-Duffy, L., Edwards, S., Russell, S., Hotopf, E., Perel, P. Free, C. (2018). The effectiveness of smoking cessation, physical activity/diet and alcohol reduction interventions delivered by mobile phones for the prevention of non-communicable diseases: A systematic review of randomised controlled trials. PloS one, 13(1), e0189801. doi:10.1371/journal.pone.0189801

37. Marcolino MS, Oliveira JAQ, D'Agostino M, Ribeiro AL, Alkmim MBM, Novillo-Ortiz D. The Impact of mHealth Interventions: Systematic Review of Systematic Reviews. JMIR Mhealth Uhealth. 2018;6(1):e23.

38. Laurie J, Blandford A. Making time for mindfulness. Int J Med Inform. 2016;96:38-50.

39. Collins M, Shattell M, Thomas SP. Problematic Interviewee Behaviors in Qualitative Research. Western Journal of Nursing Research. 2016;27(2):188-99.

40. Michie S, Richardson M, Johnston M, Abraham C, Francis J, Hardeman W, et al. The behavior change technique taxonomy (v1) of 93 hierarchically clustered techniques: building an international consensus for the reporting of behavior change interventions. Ann Behav Med. 2013;46(1):81-95.

41. Mohr DC, Cuijpers P, Lehman K. Supportive accountability: a model for providing human support to enhance adherence to eHealth interventions. J Med Internet Res. 2011;13(1):e30.

42. Andersson G. Using the Internet to provide cognitive behaviour therapy. Behav Res Ther. 2009;47(3):175-80.

For peer review only

For peer review only - http://bmjopen.bmj.com/site/about/guidelines.xhtml

43. Michie S, van Stralen MM, West R. The behaviour change wheel: a new method for characterising and designing behaviour change interventions. Implement Sci. 2011;6:42.

44. Walsh JC, Corbett T, Hogan M, Duggan J, McNamara A. An mHealth Intervention Using a Smartphone App to Increase Walking Behavior in Young Adults: A Pilot Study. JMIR Mhealth Uhealth. 2016;4(3):e109. Published 2016 Sep 22. doi:10.2196/mhealth.5227

45. Geraghty AW, Torres LD, Leykin Y, Perez-Stable EJ, Munoz RF. Understanding attrition from international Internet health interventions: a step towards global eHealth. Health Promot Int. 2013;28(3):442-52.

46. Mistler LA, Ben-Zeev D, Carpenter-Song E, Brunette MF, Friedman MJ. Mobile Mindfulness Intervention on an Acute Psychiatric Unit: Feasibility and Acceptability Study. JMIR Ment Health. 2017;4(3):e34.

47. Noone C, Hogan MJ. A randomised active-controlled trial to examine the effects of an online mindfulness intervention on executive control, critical thinking and key thinking dispositions in a university student sample. BMC Psychol. 2018;6(1):13.

48. Wen L, Sweeney TE, Welton L, Trockel M, Katznelson L. Encouraging Mindfulness in Medical House Staff via Smartphone App: A Pilot Study. Acad Psychiatry. 2017;41(5):646-50.

49. Morrison Wylde C, Mahrer NE, Meyer RML, Gold JI. Mindfulness for Novice Pediatric Nurses: Smartphone Application Versus Traditional Intervention. J Pediatr Nurs. 2017;36:205-12.

50. Lim D, Condon P, DeSteno D. Mindfulness and compassion: an examination of mechanism and scalability. PLoS One. 2015;10(2):e0118221.

51. Arean PA, Hallgren KA, Jordan JT, Gazzaley A, Atkins DC, Heagerty PJ, et al. The Use and Effectiveness of Mobile Apps for Depression: Results From a Fully Remote Clinical Trial. J Med Internet Res. 2016;18(12):e330.

For peer review only - http://bmjopen.bmj.com/site/about/guidelines.xhtml

52. Gilbody S, Lewis H, Adamson J, Atherton K, Bailey D, Birtwistle J, et al. Effect of Collaborative Care vs Usual Care on Depressive Symptoms in Older Adults With Subthreshold Depression: The CASPER Randomized Clinical Trial. JAMA. 2017;317(7):728-37.

53. Brett J, Staniszewska S, Mockford C, Herron-Marx S, Hughes J, Tysall C, et al. A systematic review of the impact of patient and public involvement on service users, researchers and communities. Patient. 2014;7(4):387-95.

For peer review only

For peer review only - http://bmjopen.bmj.com/site/about/guidelines.xhtml

APPENDIX 1

**Feature**	**Positive aspects**	**Negative aspects**	**Solutions**
**Voice**	Liked by some participants, considered soothing and even spiritual	The voice was not relaxing. One had to listen carefully which meant you could not switch off; otherwise you could not hear instruction clearly during meditation.<br><br>It was also too repetitive, after 10 times it felt like a chore and not something to look forward to.	Choice of different voices<br><br>participants should be told it takes time to get into the rhythm of the instructions, but you can get used to it
**General interface aesthetics**	colours really fresh, interface not too busy, with pleasing layout and aesthetics	one participant would prefer different, bright, colours such as purple	None needed
**Graphics**		The pictures were often not appropriate as they focused on the problem (e.g. tooth pain logo) rather than something positive or soothing. Unanticipated problems could arise according to one participant; she found it hard to lose weight and felt the food icons a) did not represent success and b) reminded her of eating.<br><br>One participant found the pictures hard to see.	Psychological theory supports the view that positive images would be advisable

For peer review only

**Animations**	liked	poorly chosen	None needed
**Progress**	One participant liked the way the app helped progress through the stages.<br><br>Another participant, with cyclical pain and hence cyclical use, did not aim to progress but simply chose to use the baseline video each time.	Three participants were frustrated and even 'angry' and 'patronised' at having to do the same (basic) meditation several times before moving up a stage.<br><br>There was no indication of goals, duration of each level or gamification rewards.	Goal setting is recommended by psychological theory and could be as simple as using "day 1 of 30" rather than simply Day 1.<br><br>Changing emojis could show progress, for example from a sad face to a smiley face to a heart; the inbuilt progress function was not felt sufficiently motivating.
**Introduction**		Experienced app users suggested the introduction could be improved for initiates	overview of the entire app and its levels suggested for the start.<br><br>One participant had done mindfulness before but thought an introduction to mindfulness might be helpful for others.
**Enjoyment**	considered fun by some participants.	One said it was not very 'interesting'.	Include examples of a patient's day with the app to cater for a greater variety of participants<br><br>It would be good to have other features as drop down options that were fun and

For peer review only

			did not involve meditation.

**Session intensity**		Would be good to choose the duration of the meditation.	15 or 20 mins would be better than 10, which is not enough time shut off and meditate deeply.<br><br>The option to control this was important as even 10 minutes was a difficult commitment for some busy participants (1074, 1075, 1078) or those battling with chronic fatigue (1074)
**Pain modules**		it can be hard to focus on something like using the app when you are in pain.	None needed

Comments on app acceptability and usability made by n=13 patients. Two patients walked through the app with the researcher

For peer review only

Reporting checklist for qualitative study.

Based on the SRQR guidelines.

Instructions to authors

Complete this checklist by entering the page numbers from your manuscript where readers will find each of the items listed below.

Your article may not currently address all the items on the checklist. Please modify your text to include the missing information. If you are certain that an item does not apply, please write "n/a" and provide a short explanation.

Upload your completed checklist as an extra file when you submit to a journal.

In your methods section, say that you used the SRQR reporting guidelines, and cite them as:

O'Brien BC, Harris IB, Beckman TJ, Reed DA, Cook DA. Standards for reporting qualitative research: a synthesis of recommendations. Acad Med. 2014;89(9):1245-1251.

		Reporting Item	Page Number
	#1	Concise description of the nature and topic of the study identifying the study as qualitative or indicating the approach (e.g. ethnography, grounded theory) or data collection methods (e.g. interview, focus group) is recommended	6
	#2	Summary of the key elements of the study using the abstract format of the intended publication; typically includes background, purpose, methods, results and conclusions	2
Problem formulation	#3	Description and signifcance of the problem / phenomenon studied: review of relevant theory and empirical work; problem statement	4 and 5
Purpose or research question	#4	Purpose of the study and specific objectives or questions	5
Qualitative approach and research paradigm	#5	Qualitative approach (e.g. ethnography, grounded theory, case study, phenomenolgy, narrative research) and guiding theory if appropriate; identifying the research paradigm (e.g. postpositivist, constructivist / interpretivist) is also recommended; rationale. The	7

For peer review only

		rationale should briefly discuss the justification for choosing that theory, approach, method or technique rather than other options available; the assumptions and limitations implicit in those choices and how those choices influence study conclusions and transferability. As appropriate the rationale for several items might be discussed together.	
Researcher characteristics and reflexivity	#6	Researchers' characteristics that may influence the research, including personal attributes, qualifications / experience, relationship with participants, assumptions and / or presuppositions; potential or actual interaction between researchers' characteristics and the research questions, approach, methods, results and / or transferability	19
Context	#7	Setting / site and salient contextual factors; rationale	7
Sampling strategy	#8	How and why research participants, documents, or events were selected; criteria for deciding when no further sampling was necessary (e.g. sampling saturation); rationale	7
Ethical issues pertaining to human subjects	#9	Documentation of approval by an appropriate ethics review board and participant consent, or explanation for lack thereof; other confidentiality and data security issues	6
Data collection methods	#10	Types of data collected; details of data collection procedures including (as appropriate) start and stop dates of data collection and analysis, iterative process, triangulation of sources / methods, and modification of procedures in response to evolving study findings; rationale	7
Data collection instruments and technologies	#11	Description of instruments (e.g. interview guides, questionnaires) and devices (e.g. audio recorders) used for data collection; if / how the instruments(s) changed over the course of the study	8
Units of study	#12	Number and relevant characteristics of participants, documents, or events included in the study; level of participation (could be reported in results)	See note 1
Data processing	#13	Methods for processing data prior to and during analysis, including transcription, data entry, data management and security, verification of data integrity, data coding, and anonymisation / deidentification of excerpts	8
Data analysis	#14	Process by which inferences, themes, etc. were identified and	8

For peer review only

		developed, including the researchers involved in data analysis; usually references a specific paradigm or approach; rationale	
Techniques to enhance trustworthiness	#15	Techniques to enhance trustworthiness and credibility of data analysis (e.g. member checking, audit trail, triangulation); rationale	8
Syntheses and interpretation	#16	Main findings (e.g. interpretations, inferences, and themes); might include development of a theory or model, or integration with prior research or theory	8-16
Links to empirical data	#17	Evidence (e.g. quotes, field notes, text excerpts, photographs) to substantiate analytic findings	See note 2
Intergration with prior work, implications, transferability and contribution(s) to the field	#18	Short summary of main findings; explanation of how findings and conclusions connect to, support, elaborate on, or challenge conclusions of earlier scholarship; discussion of scope of application / generalizability; identification of unique contributions(s) to scholarship in a discipline or field	16
Limitations	#19	Trustworthiness and limitations of findings	19
Conflicts of interest	#20	Potential sources of influence of perceived influence on study conduct and conclusions; how these were managed	4
Funding	#21	Sources of funding and other support; role of funders in data collection, interpretation and reporting	3

Author notes

1. 7,8,9 and especially 10

2. 8-16 and appendix

The SRQR checklist is distributed with permission of Wolters Kluwer © 2014 by the Association of American Medical Colleges. This checklist was completed on 26. March 2019 using https://www.goodreports.org/, a tool made by the EQUATOR Network in collaboration with Penelope.ai

For peer review only

BMJ Open

Mhealth – Providing a Mindfulness App for women with chronic pelvic pain in gynaecology outpatient clinics: Qualitative data analysis of user experience and lessons learnt

Journal:	*BMJ Open*
Manuscript ID	bmjopen-2019-030711.R2
Article Type:	Original research
Date Submitted by the Author:	30-Nov-2019
Complete List of Authors:	Ball, ELizabeth; Queen Mary University of London - Whitechapel Campus, Yvonne Carter Building<br>Newton, Sian; QMUL<br>Rohricht, Frank ; East London NHS Foundation Trust<br>Steed, Liz; QMUL<br>Birch, Judy<br>Dodds, Julie; QMUL, Women'shealth Research Unit<br>Cantalapiedra Calvete, Clara; QMUL, Women's Health Research Unit<br>Taylor, Stephanie; QMUL, Center for Primary Care and Population Health<br>Rivas, Carol; University College London
**Primary Subject Heading**:	Obstetrics and gynaecology
Secondary Subject Heading:	Health informatics
Keywords:	Chronic pelvic pain, mHealth, mindfulness, patient engagement, health app, feasibility study

SCHOLARONE™
Manuscripts

I, the Submitting Author has the right to grant and does grant on behalf of all authors of the Work (as defined in the below author licence), an exclusive licence and/or a non-exclusive licence for contributions from authors who are: i) UK Crown employees; ii) where BMJ has agreed a CC-BY licence shall apply, and/or iii) in accordance with the terms applicable for US Federal Government officers or employees acting as part of their official duties; on a worldwide, perpetual, irrevocable, royalty-free basis to BMJ Publishing Group Ltd ("BMJ") its licensees and where the relevant Journal is co-owned by BMJ to the co-owners of the Journal, to publish the Work in this journal and any other BMJ products and to exploit all rights, as set out in our licence.

The Submitting Author accepts and understands that any supply made under these terms is made by BMJ to the Submitting Author unless you are acting as an employee on behalf of your employer or a postgraduate student of an affiliated institution which is paying any applicable article publishing charge ("APC") for Open Access articles. Where the Submitting Author wishes to make the Work available on an Open Access basis (and intends to pay the relevant APC), the terms of reuse of such Open Access shall be governed by a Creative Commons licence – details of these licences and which Creative Commons licence will apply to this Work are set out in our licence referred to above.

Other than as permitted in any relevant BMJ Author's Self Archiving Policies, I confirm this Work has not been accepted for publication elsewhere, is not being considered for publication elsewhere and does not duplicate material already published. I confirm all authors consent to publication of this Work and authorise the granting of this licence.

For peer review only

Mhealth – Providing a Mindfulness App for women with chronic pelvic pain in gynaecology outpatient clinics: Qualitative data analysis of user experience and lessons learnt

Ball, Elizabeth
Department of Obstetrics and Gynaecology, Barts Health NHS Trust, United Kingdom, Women's Health Research Unit, Barts and the London School of Medicine and Dentistry, Queen Mary University of London, United Kingdom, Centre for Maternal and child Health Research, City University London

Newton, Sian
Centre for Primary Care and Population Health, Queen Mary University of London, United Kingdom

Rohricht, Frank
East London NHS Foundation Trust, United Kingdom

Steed, Liz
Centre for Primary Care and Population Health, Queen Mary University of London, United Kingdom

Birch, Judy
Pelvic Pain Support Network, UK

Dodds, Julie
Women's Health Research Unit, Barts and the London School of Medicine and Dentistry, Queen Mary University of London, United Kingdom

Cantalapiedra Calvete, Clara
Department of Obstetrics and Gynaecology, Barts Health NHS Trust, United Kingdom

Taylor, Stephanie JC
Centre for Primary Care and Population Health, Queen Mary University of London, United Kingdom

Rivas, Carol
Department of Social Science, University College London, United Kingdom

Corresponding author

Carol Rivas, Department of Social Science, University College London, United Kingdom
E-mail: c.rivas@ucl.ac.uk

For peer review only

Abstract

Objective

To determine whether a pre-existing smartphone App to teach Mindfulness Meditation is acceptable to women with chronic pelvic pain (CPP) and can be integrated into clinical practice within NHS CPP pathways. To inform the design of a potential randomised clinical trial.

Design

A pre-study patient and public involvement (PPI) group to collect feedback on the acceptability of the existing app and study design was followed by a three-arm randomised feasibility trial. In addition, we undertook interviews and focus groups with patients and staff to explore app usability and acceptability. We also obtained participant comments on the research process, such as acceptability of the study questionnaires.

Setting

Two gynaecology clinics within Barts Health NHS, London, UK.

Participants

Patients with CPP lasting $\geq$6 months with access to smartphone or PC and understanding of basic English.

Intervention

The intervention was mindfulness meditation content plus additional pain module delivered by smartphone app, active controls received muscle relaxation content by the same app. Passive (waiting list) controls received usual care.

Main outcome measures

Themes on user feedback, app usability and integration and reasons for using/not using the app.

Results

App use was low in both active groups.

Patients in the pre-study PPI group, all volunteers, were enthusiastic about the app (convenience, content, portability, flexibility, ease of use). Women contributing to the

For peer review only - http://bmjopen.bmj.com/site/about/guidelines.xhtml

interview or focus group data (n=14), from a ‘real world’ clinic, (some not regular app users) were less positive, citing as barriers lack of opportunities/motivation to use the app, and lack of familiarity and capabilities with technology. Staff (n=7) were concerned about the potential need for extra support for patients and staff and considered the app needed organisational backing and peer acceptance.

Conclusion

The opinions of pre-study PPI volunteers meeting in their private time may not represent those of patients recruited at a routine clinic appointment.

It may be more successful to co-design/co-develop an app with typical users than to adapt existing apps for use in real-world clinical populations.

Trial registration and funding

The trial (ISRCTN 10925965) was funded by the UK National Institute of Health Research, Research for Patient Benefit programme (RfPB PB-PG-1013-32025).

Keywords

Chronic pelvic pain, mHealth, mindfulness, Headspace, PPI, patient engagement, feasibility study, health app

For peer review only - http://bmjopen.bmj.com/site/about/guidelines.xhtml

Article Summary; 'Strengths and limitations of this study' (relating to methods)

- The study was designed with the help of a study design group of CPP patients
- Patient recruitment to the study was good
- Unusually our study focused on a deprived urban area of the UK and considered typical local clinical patients
- The qualitative evaluation included the perspectives of both patitents and a variety of healthcare staff
- Patients in the qualitative evaluation preferred telephone interviews over the offered face to face focus groups.

The original protocol for the study has been published separately. The UK National Institute of Health Research supported this work (RfPB PB-PG-1013-32025). There are no competing interests.

Introduction

Smartphone health apps, as one form of mhealth (1), are popular in the UK, our study setting. With more than two-thirds of the UK population using smartphones (2,3,4), health apps are one of the fastest growing app categories, thus numbers of users are still increasing (5). Currently these apps are usually developed either by researchers or (in the majority) by commercial companies, without collaboration between these groups (6,7). The lack of interaction between researchers and commercial developers in the field of pain-related apps has led to a situation where commercially available apps have not been scientifically validated and apps that have been developed from research projects are not commercially available (8).

For peer review only - http://bmjopen.bmj.com/site/about/guidelines.xhtml

We were interested in using an app to support women with chronic pelvic pain (CPP) in a clinical setting, where validation of an intervention is important to ensure best care. CPP is defined as a subjective physical and emotional experience of pain in the pelvic area that has been present for at least six months that may or may not have an identifiable pathology (9). CPP affects up to 24% of women worldwide (10) and accounts for 20% of gynaecological clinic referrals. (11,12) It has considerable impact on patients' quality of life, including their mental health and their income (13) due to loss of working days and diminished work capacity. Annual costs to the NHS have been estimated at approximately £326 million (14). For endometriosis alone, which is just one cause of CPP, a European study of over 900 women showed average annual total costs per woman of €9579. Costs of productivity loss of €6298 were double the health care costs of €3113 per woman. The latter were due to surgery (29%), monitoring tests (19%) and hospitalization (18%) and physician visits (16%) (15).

Despite costly interventions, CPP is often resistant to surgical and medical treatment and appears to respond better to a multimodal, holistic approach, (16) with a focus on coping strategies. A systematic review of randomised controlled trials (RCTs) by authors has identified mindfulness meditation (MM) as an effective coping strategy in other chronic pain conditions (17). In addition, evidence from uncontrolled trials suggests positive effects of MM for CPP, such as an increased ability to control pain, improvements in mental health, emotional well-being, work and family life and social functioning (18,19), but these have never been examined in an RCT.

We therefore chose to evaluate MM delivered via an app to women with CPP as our intervention. CPP is especially common in younger women, who may be categorised as digital natives, making an app-based intervention particularly appropriate in this group.

In MM the aim is to keep focussed on one's own breathing. Whenever attention wanders to intrusive thoughts and feelings these are simply taken notice of in a neutral way, that is,

For peer review only - http://bmjopen.bmj.com/site/about/guidelines.xhtml

without elaboration or judgements or consideration of action. They are then let go as attention is returned to the breath. The idea is to bring awareness back to the here and now whenever worries and troubles intrude into thoughts.(20)

Generally two main complementary approaches have been used for MM: 1) exercises focusing one's attention to the present moment, and 2) monitoring of experiences in the present moment. While systematic reviews show that MM may have positive effects on depression, quality of life and pain symptoms in patients with chronic pain (17,21,22) and apps with such a focus on chronic disease have been shown to be beneficial in various conditions (23) none of the reviewed papers included meditation delivered via mobile phone apps or in women with CPP.

Evaluation of an existing app is often appropriate (24) and is both quicker and more cost-effective than designing an app from scratch. We chose to evaluate an existing commercial app platform that teaches mindfulness by guided meditation (Headspace ®), with a ten day basic meditation module followed by a pain module specifically designed for the MEMPHIS (Mindfulness meditation using a smart-phone application for women with chronic pelvic pain) study. The Headspace app was publicly nominated favourite health app of 2013,(25) has a 5 star user rating in the Apple™ app shop and has scored top in a systematic review of 23 mindfulness apps using the Mobile Application Rating Scale (visual aesthetics, engagement, functionality or information quality) (26). Headspace had reportedly seen over 15 million downloads up to mid-2018 when our study began.(27) To our knowledge the Headspace app in its original or modified form has not been assessed in any other pain conditions.

We undertook a three-arm parallel randomised feasibility trial (MEMPHIS) (28) to assess whether or not to proceed with a full randomised controlled trial of the modified Headspace meditation app for women with CPP. In the current paper we report on the qualitative interview and focus group data from this study; the protocol and quantitative results have been

For peer review only - http://bmjopen.bmj.com/site/about/guidelines.xhtml

published/ submitted (29,30). Our aim for the qualitative part of this study was to determine whether a pre-existing smartphone app to teach MM is acceptable to women with CPP and can be integrated into clinical practice within NHS CPP pathways. Objectives were to consider:

1) The acceptability, use and usability of the app in the intended service user population and for health care professionals (doctors, health care assistants, clinical and research nurses)

2) The feasibility of integrating such an app into existing healthcare pathways

3) The usefulness of having a distinct patient group to advise us on the study design.

Methods

Outcomes

The outcomes of this analysis were inductively derived descriptive themes on acceptability, use and usability of the app and feasibility of integrating it into existing pathways. We follow the ISO 9241-11 (https://www.iso.org/obp/ui/#iso:std:iso:9241:-11:ed-2:v1:en) concept of technology usability (user friendliness) as the extent to which the app could be satisfactorily used by participants to meditate. By acceptability we mean whether participants could see a reason for using the app when given in clinic, and would be happy to use it for meditation.

Intervention Procedures

Women in the mindfulness meditation group received access to a 60-day progressive mindfulness meditation course delivered via the Headspace app. The first 10 days of the course taught basics of mindfulness meditation. Following this, participants were able to access the module on meditation which was targeted at for chronic pain. This module had

For peer review only - http://bmjopen.bmj.com/site/about/guidelines.xhtml

been specifically made for this study. Session length was 10 minutes for the first 10 days, 15 min up to day 20 and 20 min up to day 60. The active control group received access to a series of muscle relaxation sessions. These sessions were identical every day, except that their duration increased to mirror the increasing duration of the meditation content being listened to by the intervention group. Usage data are reported elsewhere (30).

Patient and Public Involvement

We held a Patient and Public Involvement (PPI) group workshop before the study to discuss acceptability of the Headspace app and help us design our study. Women attending the Royal London Hospital CPP clinic were invited to volunteer for a week of using the unmodified (normal commercially available) Headspace app (which did not have the pain module at the time we undertook our pre study workshop) and then feed back on their experiences with the app in an evening discussion group. Women were not involved in the design of the modified app. The focus for the PPI group was on the use of the generic MM app.

Two patient representatives provided support from the study design stage through recruitment to the interpretation of the results and regularly attended Trial Management Group meetings.

For peer review only

Study recruitment and eligibility

The trial recruited at two outpatient gynaecology clinics within Barts Health NHS trust in two separate deprived areas of inner East London. Female patients with new or follow-up gynaecology appointments were assessed for eligibility by a researcher in clinic, having been posted a Patient Information Sheet. Women were eligible if they had been suffering with CPP for 6 months or more and had at least a basic understanding of the English language, sufficient to follow instructions, as assessed during discussion about the study for

For peer review only - http://bmjopen.bmj.com/site/about/guidelines.xhtml

informed consent; no women were excluded on this basis. Women were excluded if they did not meet these criteria or they did not have access to a smartphone or personal computer or were currently using the Headspace app (there were very few of the latter, according to the impression of the recruiting nurses). All patients gave full and informed consent to be randomised and data were collected through all stages of the study. All healthcare professionals and research nurses involved in the two clinics were also invited to take part in the feasibility study as the only eligibility criterion for staff. Full enrolment data are provided in Forbes et al (30). A key difference of these patients from those in the PPI group was that their focus was on managing their pain, with the app given explicitly as part of their clinic management support.

For the study of quantitative data, 90 patients were allocated randomly in a 1:1:1 ratio to the mindfulness meditation app, a muscle relaxation app active control or the usual care arm (for full details see (29)). Patients in the two active arms were asked to download the modified app in the clinic with support from a research staff member and were sent a questionnaire about app usability, an analysis of which is reported in a companion paper.(30) We used data from the app usability questionnaire to inform topic guides for the qualitative part of the study. This outlined key usability issues that had been uncovered, to guide our semi-structured interviews and focus groups with patients and staff.

All women in the intervention and active control arms were eligible for the qualitative component of the study, and all staff participating in the study.

Within-study interviews and focus groups

All patients in the intervention and active control arms were invited to one of two focus groups at their own study site after the 6-month follow up. We offered telephone interviews as an alternative. Patients were asked to 'walk through' the app with researchers, articulating their thought processes while they did so and commenting on its different specific features,

For peer review only - http://bmjopen.bmj.com/site/about/guidelines.xhtml

(31). Walkthroughs are often used in developing technologies such as mhealth. This helped to identify app usability issues or barriers to use of the app from the users' points of view without the need for technical discussions. Results for the walkthrough, showing comments on different features specific to the usability of the intervention app used in our study are shown in Appendix 1; walkthroughs were undertaken by two patients. Patients also discussed with us their experiences around app usability and acceptability.

Staff were invited to attend a staff focus group overseen by the patient representative and facilitated by a researcher. In addition to considering app usability and acceptability, members of the staff focus group (consultants, health care assistants, clinical and research nurses and a representative from the pelvic pain support network were eligible) were asked about the ease of integration into existing NHS pathways. Part of the staff discussion was free flowing with open-ended questions, which gave us patient-focussed information on app acceptability, and part was structured using questions developed from the Normalisation Process Theory (NPT) toolkit in the way recommended by the NPT developers (32). For example, we asked whether staff could see a purpose to the app in clinical practice, as adding something different, which corresponds to the NPT toolkit question 'Participants distinguish the intervention from current ways of working'. Since this was a semi-structured approach questions were not rigidly worded. This helped us to consider the feasibility of integration of the app into practice. NPT is a theory of implementation practices that was initially developed for consideration of technology implementation and is in common use (32).

All data were audio-recorded at point of collection and transcribed, with personal identifying data removed from transcripts. Raw data were stored in a Primary Care Clinical Trial Unit database to clinical trial standards.

Analysis

For peer review only

For peer review only - http://bmjopen.bmj.com/site/about/guidelines.xhtml

Analysis of within-study focus groups and interviews was carried out blinded as to which study app was used, and deployed the immersion-crystallisation method (33). Thus, the lead qualitative researcher immersed herself in the data, reading transcripts carefully, then writing down articulated or crystallised patterns or themes that related to the aims and research questions of the study. These were discussed with another researcher from the team, and themes modified as appropriate. This process was repeated until all the data had been examined and all patterns that had been noticed were articulated, discussed and substantiated with exemplar extracts. This approach was considered appropriate since we had a small dataset and we were not aiming to develop conceptual themes but rather to inform the design and development of a randomised controlled trial for the modified app. We used the SRQR checklist when writing our report (34).

Ethics

The MEMPHIS trial was a three-arm parallel randomised feasibility trial approved by Camden and Kings Cross Research Ethics Committee in 2016 (15/LO/1967).

Results

We screened 488 women between May and September 2016 for their eligibility to participate in the study. After exclusions, 90 women gave full consent to participate and were randomised to the intervention arm (31 women), the active control arm (30 women) or the usual care arm (29 women).

Demographics

Women in the main feasibility trial (30) were aged a mean of 35 years, 66% were employed and overall approximately 50% had stayed in full time education until at least the age of 20 though the proportion was least, at 36.7% in the intervention arm. Overall 44% were of white ethnicity though the proportion was lowest in the intervention arm (35.7%) and highest in the usual care arm (53.6%). The second most common self-reported ethnic groups were

For peer review only - http://bmjopen.bmj.com/site/about/guidelines.xhtml

‘Southern Asian’ and ‘Black’. Women in the intervention arm were most likely to have experienced CPP for 3-5 years (40.3% of this group), women in the usual care group for over 10 years (42.9% of this group). More women had pain for longer than two years in the intervention arm than in either of the other two arms. All women reported a high pain intensity, with means of 6.8 to 6.9 in the previous week (on a scale from 1-10) (30). These and other demographic data are reported in more detail in Forbes *et al*. (30) Our qualitative sample was taken from the two active arms and was comprised of 16% of trial participants and 23% of those eligible for the qualitative study. We did not record separate demographic data for the women in this smaller sample.

For peer review only

For peer review only - http://bmjopen.bmj.com/site/about/guidelines.xhtml

Pre-study PPI group

The ten women in the pre-study PPI group were self-selected local women who were familiar with using apps and focused on app use per se. They anticipated no technical issues even in women who were not used to apps. They considered that the Headspace app would be successfully adopted by patients taking part in the study, given that a smartphone, like CPP, is ‘always with you’. They praised the flexibility of the app, welcomed its portability and were unanimous in saying it was easy to slip off for 10 minutes when at work to use it. As a result, they found they could use it at times when they most needed pain relief as well as to prevent pain and found the app helpful in relieving pain and stress. The group reported being able to meditate without the app, once they had tried it with the app; however, they still preferred to use the app because they found the voice soothing.

App usage in the study

Patient usage of the app was less than expected from our pre-study PPI group discussions. Only 36% of meditation app patients and 46% of the active control patients used the app at least once. (30)

Thematic analysis of within-study data

Qualitative data were obtained from 14 study patients; 12 preferred a telephone interview, two attended face-to-face interviews, one participant at the University attached to one of the recruiting clinics and one participant at the other recruiting hospital. Patients chose not to attend focus groups. Four of the patients were from the active control arm and 10 from the intervention arm. The two women we met face to face had both used the intervention and neither had progressed beyond the training stage, something that we cannot discount for other participants and which may help to explain reports of lack of effect on pain. Seven people attended the staff focus group: two recruiting nurses, three clinic nurses one consultant and a representative from the pelvic pain support network.

For peer review only - http://bmjopen.bmj.com/site/about/guidelines.xhtml

The qualitative analysis revealed three main themes from all within-study interviews and focus groups combined regarding usability, and four subthemes:

1. Familiarity and capabilities with app technology
2. Motivations to use the app
    - Perceived benefits
    - Relation to other therapies
3. Opportunities to use the app:
    - Technology issues getting in the way
    - Life getting in the way

These are explored below. As the PPI group data were not research data we did not analyse them for themes.

While we initially combined active control and intervention groups in our analysis, we then looked for instances where there was a difference between these two groups. Only where we found this difference in any theme or statement have we specified which group women belonged to.

Familiarity and capabilities with app technology

Around half of the patients were sufficiently familiar with technology and apps to be comfortable using the study apps. However, six participants (all using the intervention, which was more complex than the active control) reported difficulty because they were *"not very good at technology*" (patient 1002, intervention), or were unsure how to get started or use the app effectively without help.

> *I am not good with technical some things that is why the problems I had arisen, right okay. So I consulted with my daughter and she helped me work it out… so I don't try everything. (patient 1002, intervention)*

One further patient *(1001, intervention)* was not used to technical app language; 'help' suggested emotional support to her, for example. Two more (one intervention, one active

control) changed handsets and therefore did not continue with the app. In all cases these technical difficulties appeared to lead to abandoning of the app or restricted use of its functionality.

Five patients having technical problems suggested possible solutions such as a 'class' or group for first time users, a YouTube orientation video, or a pictorial leaflet. This might include an introduction to meditation and mindfulness as well as the app itself. One woman commented: *"If your market is targeting people who are not using apps then you are going to have to get together and find ways to do this",* (patient 1041, intervention); she also suggested we could get ideas from other apps on the market in this regard.

Given their experiences in the study, staff were concerned about additional staff time needed to support women in using the app. This would sit in tension with one of the original rationales behind choosing an app as the mode of delivery, which was to increase the effective use of staff contact time with patients. Language barriers might compound problems.

Motivations to use the app

Staff, though unaware of the low sustained app use in the study, felt it would be common sense to hold occasional motivating meetings with patients if the intention was for them to use the app long term. The patient data suggested the main motivators or lack of motivation for using the app in our sample, which could be drawn on in such meetings, and which we now consider.

Perceived benefits

Three intervention arm patients said they only entered the trial to help others through research but were already using alternative forms of pain control. They explained that this meant they were not motivated to actually use the app, perceiving the relative benefit to be

For peer review only - http://bmjopen.bmj.com/site/about/guidelines.xhtml

small. The failure of such altruism to extend to using the app is a recognised phenomenon in clinical trials and has been called 'weak altruism'. (35) Thus, only one of these three patients persevered. Even though she was one of the women who experienced difficulties with the technology, she explained, "*with something that is as soul destroying as the pain, it is important to help others off the back of other people's misfortune as it were"* (1036, intervention). However, she wondered how relevant her data were:

I took steps to improve my situation from a weight loss perspective as well and I've lost a lot of weight which has significantly helped not 100% but it is has significantly helped so I felt a bit fraudulent the last time filling in the forms because , so everything had improved so much so from the medical study perspective it was more about the weight loss than the app. So I felt a bit bad that I was still taking part. (patient 1036)

There was no clear pattern regarding the impact of current pain on app use by patients. Six said they used it regardless of pain intensity – sometimes developing a daily routine - while four only used it when in severe pain or expecting to be (e.g. during menstruation). This cyclical or intermittent use in some patients – which was irrespective of study arm - should be considered when looking at our main study outcomes.(30) The Headspace app requires regular use to learn and benefit from psychological techniques. To address this, healthcare professional alerts have been effective in other studies, (36) whilst Headspace only has a reminder function that the user can set. This was often not sufficient, as one patient said even with this feature, "*To be quite honest I used it a couple of times and then forgot. And then I [remembered it and] used it more frequently." (patient 1036, intervention)*

One patient said medication was not working but the app did, though she was not sure whether this was '*mind over matter'* (1065, intervention), which was her term for a placebo effect. Three others said it did not reduce their pain; all three were using the intervention app. The remaining patients considered other benefits were good reason for using the app even when they did not feel that it reduced pain intensity. Alternative or unanticipated benefits were not formally measured or taken into account in the study's effectiveness

For peer review only - http://bmjopen.bmj.com/site/about/guidelines.xhtml

outcomes (30). For example, ten patients valued the way the app helped them to relax or de-stress or focus and re-assess their life; three of these specifically said they used it to induce a relaxed state to get to sleep. Notably the active control was a relaxation app; however this benefit was also reported by many women in the intervention arm. One participant (active control) said she did not like the focus on pain per se as her condition impacted on various areas of her life. Even when the intervention app was positively received, women might stop using it because it was too powerful, and they had gained the change they wanted:

I think it was day 3, I could see the change that was happening, I was able to speak up for myself ……I can't explain it, even now I am getting emotional… it's just a lack of focus, I just needed direction. To try and put It into words. To me it meant so much that I have gone back to church… I use it outside of the app now I have got from it what was missing, so it's done something to me and for me which is very positive, and I may try it to lose weight but those positive vibes are still there. I can't go back to it because I did not want to go any further because what I got at the time helped me to focus, to change my way of thinking. I used it for about two or three weeks. (patient 1001, intervention)

Three intervention app patients found the app put them more in tune with their bodies and their breathing, (two of these were among those who also found the app de-stressing) while another found yoga better for that. Six patients, like pre-study PPI group members, also learned to use techniques from the app to alter their stress patterns without the app, having tried it, for example in traffic or by sitting down and taking time out or for general relaxation.

<u>Relation to other therapies</u>

Two patients (both active control) preferred 'pure' meditation, another considered the app to be "*very much about meditatio*n" (1041, intervention), which is in keeping with the arms they were in. An alternative therapy practitioner and two further patients reported that they preferred yoga. One (active control) said this was because it focussed on each part of the body in turn.

For peer review only - http://bmjopen.bmj.com/site/about/guidelines.xhtml

Three intervention app patients thought the app was useful as an adjunct to other methods rather than a replacement for them, for example physical interventions such as Pilates, or listening to classical music.

Opportunities to use the app

Technology issues getting in the way

Staff pointed out that not all patients had smartphones (not appreciating that PCs/tablets were alternatives allowed in this study). Some patients lacked the storage space to load the app on their phones. There were also issues with Wi-Fi connectivity when staff tried to help the patients load the app within the hospital sites. Possible solutions that staff suggested were to lend patients phones and to have group upload sessions in a location with good Wi-Fi signal – though they acknowledged the resource implications.

Life getting in the way

Seven patients revealed they preferred to use the app in the evenings because of other life commitments. This meant they did not always use it as a direct response to pain, reducing its potential for contemporaneous effect. One patient who used it in response to pain but only used it once or twice blamed this on having no spare time because of juggling work and children (active control); however, another patient (intervention) managed despite such commitments the fact that she was in the intervention arm may have played a role.

Barriers to integration for staff

Staff believed that the biggest barrier to clinical adoption of the app was a possible lack of support from the host organisation. It might also be hard to integrate the app within existing professional work practices if the staff in the position of offering the app to patients failed to see its relative advantage over other interventions. Collection of feedback on the app's effectiveness would be necessary for staff to support sustained use. It was felt that staff would need training on how to introduce the app to women in practice, and that complexity

For peer review only - http://bmjopen.bmj.com/site/about/guidelines.xhtml

and high staff turnover could impede sustained use. An app was also seen as impersonal compared with face-to-face contact, which was more favoured by staff.

Participant comments on the research process

The study questionnaires that were used for the main quantitative outcome measures (30) were acceptable to patients except for some discomfort with a question about sex, which patients considered a delicate question that was missing a 'no sex' option. Most preferred a paper form reflecting their lack of affinity with technology. There were no indications that the study design or study processes had contributed to the participants' lack of engagement with the apps – with a caveat around support with the technology as mentioned above - though we did not systematically consider this. A full summary of patient comments on the study design and procedures is given in Appendix 1.

Discussion

Our study adds to the limited evidence on mHealth app user behaviour and experience (36,37). The pre-study PPI group (young women, of a generation who were familiar with using apps and who were asked to focus on the study design use of the app) liked the idea of delivering the intervention via an app, praising the contemporary design and flexibility. Hence we expected a similar positive attitude from trial participants, who were of a comparable age, and we assumed would be keen on using apps. Participant feedback revealed that this assumption was too simplistic.

Using our qualitative data, we were able to explore reasons for low app usage that had been recorded in our feasibility study.(30) Our thematic analysis suggests that the low app use in the trial occurred because many patients were not familiar with apps in general or lacked capabilities with technology. This was particularly true for the more complex intervention app. The other themes we report did not differ between groups (although the three cases of weak altruism' all occurred in the intervention arm) which suggests more generic issues that

For peer review only - http://bmjopen.bmj.com/site/about/guidelines.xhtml

can be transferable to other app use situations. For example, women stated limited motivation to use the app because of a lack of perceived benefit, or a lack of opportunity to use the app due to Wi-Fi issues or due to other commitments.

Similar findings were reported by Laurie *et al*.(38) who interviewed 16 healthy city-dwelling participants (25-38 years) about their user behaviour before and after 30-40 days of Headspace app exposure. Like us, they reported barriers of busy lives, failure to establish a routine and a lack of perceived benefit; all users in their study tried the app at least once hoping it could deliver a quick fix but were disappointed if this did not happen. In our study many patients failed to perceive a benefit from using the app. Hence excuses stating other commitments may mask a deeper lack of motivation linked to perceptions of benefits.(39)

The advantages and disadvantages of using the app stand-alone were also illustrated by our data. Some suggestions made by participants to improve usage, such as more guidance at the start, seem obvious in hindsight. But they had not been considered because of the feedback from the pre-study PPI group and the commercial success of Headspace. The use of community contacts may be a helpful alternative.(40) Social support can create a community of practice, help to clarify expectations,(41) and improve health outcomes (as shown for example in internet based psychological treatment for depression (42).

The data suggest that for successful app use we need to understand what motivates individuals with clinical need to use the app for clinical reasons (which our PPI group did not focus on) and target this, for example by setting appropriate expectations. Incentivisation might also improve motivation. This could be achieved through app gamification (7), or encouragement through integration with patient-clinician face-to-face encounters, which was lacking in our study since the app was used stand-alone. The present study provided extensive initial technical support but no coaching and incentivising, in keeping with the protocol. Future app studies should take this into account. Participants in our study may

For peer review only - http://bmjopen.bmj.com/site/about/guidelines.xhtml

have also have benefitted from training and support to improve their app use capabilities and guidance on how to create more opportunities for app use – such as through sharing experiences in clinic support groups. This is in keeping with the COM-B model of behaviour change (43) which our themes matched, though this was only realised after analysis. The COM-B model says that Capability, Opportunity and Motivation are key drivers of behaviour and has been used to develop a number of complex interventions including smartphone apps (e.g. 3).

Lack of engagement after recruitment, or good initial engagement but minimal or inconsistent use, have been reported in other studies (44,45), including in Headspace trials other than our own (46, 47). Settings were a university and a psychiatric inpatients clinic, both in the USA. Inconsistent app use was noted by Wen (48) in junior doctors who used self-guided Headspace. Morrison Wylde (49) compared face-to-face MM with headspace use in novice paediatric nurses. However, unlike our study there were no recorded dropouts/non-users and also no record of whether or how long the app was used for which is an important omission.

None of these studies included a qualitative component. Yet, each of the Headspace study groups were very different, and so will likely have differed in motivations, contexts for opportunity to use the app, and incentivisations (50). While these aspects were not considered in the other studies, our use of qualitative research has enabled us to explore these in more depth. Our findings suggest these are important considerations in any study of app use and therefore this study makes a contribution to the field. For example, some of the groups in other studies may have differed from ours in likelihood of using mhealth apps in the first place, and familiarity with technology. In-patients may have more time to use the app and more support – and may also have had specific barriers to app use, such as related to setting and to illness.

For peer review only

Patients in the qualitative part of our study tended not to use apps on a regular basis (or at least apps other than simple games), and in terms of our themes, also represented in the COM-B model, may be said to have few capabilities in technology use. They therefore do not represent the typical users of the Headspace app in a commercial setting. Accessing the app regularly requires energy, time and effort, but patients with CPP often suffer from fatigue and anxiety as co-morbidities, perhaps whilst having to juggle family life and work. Therefore, this may be seen as a challenging clinical population in which to trial an app. Further Headspace trials with diabetic (NCT03274362) and pain **(**NCT03495726**)** outpatients are underway.

Our study has also shown that clear consideration of unexpected benefits should be included in future studies and these can be informed by our finding that benefits for patients may be more diffuse than anticipated (e.g. app relieving stress rather than pain). Such benefits were found in the active control as well as the intervention arm and so it may be that they represent a placebo effect though the effect could equally be real. Our data also suggest that staff benefits may be less than anticipated, as participants sometimes needed a lot of support and scaffolding in technology use at least initially.

Young age, co-morbid anxiety and low educational attainment are predictors for dropping out of web-based interventions according to studies in the field of depression. (51, 52) This may be true despite regular phone support (52) though our participants all considered active motivational support from staff or app support groups would have improved app use. Our intervention arm participants were particularly likely to be young and with low educational attainment.

Our data suggest that it is important to involve real world end users in the agile design or development or modification of apps in close collaboration with researchers and commercial app developers.(7) Although the evaluation of existing apps has been recommended as a

For peer review only - http://bmjopen.bmj.com/site/about/guidelines.xhtml

cost-effective and rapid process, (24) our findings suggest that in actual clinical practice these may be problematic.

Strengths and weaknesses of the study and in relation to other studies

One strength of this study is that it creates much-needed evidence in the field of evaluating existing health apps in a clinic population (6,8,24) and recording user experience. This provides us with lessons to be learned.

Researchers conducting interviews and focus groups were: a senior mixed methods medical sociology researcher, a recruiting nurse, a representative from the pelvic pain support network and an experienced health psychologist. Findings were similar across the data and the different backgrounds of the researchers therefore does not appear to have influenced findings. The main analysis was undertaken by the medical sociologist and so the concordance with the COM-B model is not due to background discipline bias.

We were able to recruit successfully, and we obtained valuable information from patients with CPP, who were recruited from a deprived urban area of the UK as typical local clinical patients.

However, we report a marked discrepancy between the attitudes of the pre-study PPI group of volunteer patients from the local area, who actively put themselves forward for a 7-day trial of the app, and the participants asked to take part when they attended clinics. The opinions of pre-study PPI volunteers meeting in their private time may not be representative of the opinions of patients recruited at a routine clinic appointment. Women in the PPI group were used to using apps, which had led them to be interested in the study in the first place. Whereas women in the PPI group had all trialled the app at home and work without support from us, many patients from clinic were unable to use their phone beyond calls, texts and photos. Moreover, most of the women we interviewed used the intervention app. We can only speculate as to why this is so but it does mean that concordances and divergences across the intervention and active control arm do need to be treated with circumspection.

For peer review only - http://bmjopen.bmj.com/site/about/guidelines.xhtml

To our knowledge the present observation on failure of PPI work to translate into practice in a trial has not been formally reported before, and is lacking from a recent comprehensive systematic review.(53) PPI involvement is a stipulated requirement when applying for some funding, and the present research findings should be taken into account when drafting guidelines for future PPI involvement in study planning. PPI groups are able to provide significant help and advice in any study but our findings shows the value of adding agile co-development as a requirement for app intervention development as likely to provide a more effective intervention than one informed by PPI alone. Moreover, there is a difference between app use for active clinical management (as with our study participants), and consideration of the potential for app use for this (as with our PPI group).

Implications for clinicians and policymakers

Given the patchy use of the app and the way that some participants did not manage to unlock its full functionality, and an indication of diffusion of benefit, more work is needed to see whether the app reduces pain per se. This study is a good example of the need to move away from 'one size fits all' behavioural interventions. Future studies should do more work on implementation before doing an effectiveness trial. This will enable researchers to be more nuanced about saying who the app is effective for, if at all.

Strategies to involve busy, less motivated, and less technologically experienced individuals in PPI and lay app design groups need to be further developed. These groups should include considerable scaffolding, which we have shown extends to study involvement by patients. More care is also needed to obtain PPI input that is representative of the target group, taking into account their capabilities, opportunities and motivational aspects. It may be useful to give the PPI group a small condition management task that emulates what trial participants will be required to do. Moreover, we can confirm a recent review suggesting

For peer review only - http://bmjopen.bmj.com/site/about/guidelines.xhtml

that health apps should be co- designed with users (7), rather than presenting them with a pre-existing app. These implications for our study are also generalisable to other technology studies.

What the study adds

1. A mindfulness meditation app may not necessarily be taken up by patients with CPP even when it is a commercial success in the general population. Considerable supportive scaffolding may be needed.
2. Use of existing commercial apps in actual clinical practice may be problematic compared with the agile development of apps with collaboration between researchers, clinicians, developers and end users.
3. Chronic Pain patients are interested in alternatives to drug or surgical treatments and further research is required in this area, including with MM, the benefits of which may extend beyond pain relief itself.
4. PPI groups may be more motivated to use an intervention than a real world clinical group as they are volunteers who are interested in the research topic. This may be particularly problematic for interventions that require considerable capability or motivation in use.

For peer review only

Author contributions

EB led the study as the CI. EB and CR were the main authors of the grant application for this study, and co-lead authors of the current paper. FR, SJCT, JD, JB, SN and LS contributed to the study design and initial protocol. All authors provided support throughout the trial and contributed towards the final paper. CR led on the PPI and CR and LS led the interview and focus group field work and analysis reported here. CR, LS, SN, CCC, JD and JB were all involved in the field work.

Role of the funding source

The UK National Institute of Health Research, Research for Patient Benefit (RfPB No. PB-PG1013-32025) funded the MEMPHIS study. The funder had no role in the study design, in

For peer review only - http://bmjopen.bmj.com/site/about/guidelines.xhtml

the collection, analysis, and interpretation of the data, in the writing of this report, or in the decision to submit the paper for publication. The first and last authors vouch for the integrity, completeness and accuracy of the data and analyses, and for the fidelity of this report to the protocol and statistical analysis plan. The views and opinions expressed herein are those of the authors and do not necessarily reflect those of the RfPB, NIHR, NHS or the Department of Health.

Competing interests

We have read and understood BMJ policy on declaration of interests.

Data sharing

The data are collected from a small number of people which could compromise their identity if shared with others. Therefore we are not making them available except under exceptional circumstances which will be determined by the custodian of the data (Elizabeth Ball) on an individual basis.

Transparency

The lead author confirms that the manuscript is an honest, accurate and transparent account of the study being reported; that no important aspects of the study have been omitted; and that any discrepancies from the study as planned and registered have been explained.

Acknowledgements

We would like to thank all the researchers, consultant obstetricians and gynaecologists and data assistants at each of the recruiting clinics for their hard work in promoting the study, recruiting participants and for data entry. Our thanks go to the Trial Steering Committee; Andrew Horne, Sohinee Bhattacharya, Christina Liossi, and Hulya Guzel for their constant support throughout the trial.

We thank the Pelvic Pain Support Network and Endometriosis UK for their promotion and guidance in developing the study design. We would also like to acknowledge the NIHR RfPB programme for their on-going support.

Lastly, thank you to Headspace Ltd for providing our participants with access to the Headspace platform, designing novel content for the study, and for their continuous support and advise throughout the study.

For peer review only - http://bmjopen.bmj.com/site/about/guidelines.xhtml

References

1. Ali EE, Chew L, Yap KY. Evolution and current status of mhealth research: a systematic review. BMJ Innovations 2016;2:33-40
2. Ofcom. The Communications Market Report. Ofcom: United Kingdom 2015. https://www.ofcom.org.uk/research-and-data/multi-sector-research/cmr/cmr15/uk [accessed 21 March 2019]
3. Kayyali R. Peletidi A. Ismail M, Hashim Z. Bandeira P, Bonnah J. Awareness and Use of mHealth Apps: A Study from England. Pharmacy 2017, 5, 33.
4. Sezgin E, Yildirim S, Özkan-Yildirim S, Sumuer E. Current and Emerging mHealth Technologies: Adoption, Implementation, and Use: Springer International Publishing; 2018 2018.
5. Lunden I. 6.1B Smartphone Users Globally By 2020, Overtaking Basic Phone Subscriptions https://techcrunch.com/2015/06/02/6-1b-smartphone-users-globally-by-2020-overtaking-basic-fixed-phone-subscriptions/?guccounter=12015 [cited 2018 28 August 2018].
6. Subhi Y, Bube SH, Rolskov Bojsen S, Skou Thomsen AS, Konge L. Expert Involvement and Adherence to Medical Evidence in Medical Mobile Phone Apps: A Systematic Review. JMIR Mhealth Uhealth. 2015;3(3):e79.
7. Edwards EA, Lumsden J, Rivas C, et al. Gamification for health promotion: systematic review of behaviour change techniques in smartphone apps. BMJ Open. 2016;6(10):e012447. doi:10.1136/bmjopen-2016-012447.
8. de la Vega R, Miro J. mHealth: a strategic field without a solid scientific soul. a systematic review of pain-related apps. PLoS One. 2014;9(7):e101312.
9. Engeler D, Baranowski AP, Borovicka J, et al. European Association of Urology. Guidelines on chronic pelvic pain. http://uroweb.org/wp-content/uploads/EAU-Guidelines-Chronic-Pelvic-Pain-2015.pdf. Accessed Sept 19, 2019.

For peer review only - http://bmjopen.bmj.com/site/about/guidelines.xhtml

10. Ahangari, A. Prevalence of chronic pelvic pain among women: an updated review. Pain Physician. 2014 Mar-Apr;17(2):E141-7.

11. Latthe, P., Latthe, M., Say, L., Gülmezoglu, M., & Khan, K. S. (2006). WHO systematic review of prevalence of chronic pelvic pain: a neglected reproductive health morbidity. BMC public health, 6, 177. doi:10.1186/1471-2458-6-177

12. Ayorinde AA, Macfarlane GJ, Saraswat L, Bhattacharya S. Chronic pelvic pain in women: an epidemiological perspective. Womens Health (Lond). 2015;11(6):851-64.

13. Howard F. The Role of Laparoscopy in Chronic Pelvic Pain: Promise and Pitfalls. Obstetrical & Gynecological Survey. 1993;48(6):357-87.

14. Zondervan KT, Yudkin PL, Vessey MP, Jenkinson CP, Dawes MG, Barlow DH, et al. Chronic pelvic pain in the community--symptoms, investigations, and diagnoses. Am J Obstet Gynecol. 2001;184(6):1149-55.

15. Curtis L. Unit Costs of Health and Social Care 2014 Personal Social Services Research Unit, University of Kent, Canterbury.: University of Kent, Canterbury; 2014 [Available from: https://www.pssru.ac.uk/project-pages/unit-costs/unit-costs-2014/.

16. Simoens S, Dunselman G, Dirksen C, The burden of endometriosis: costs and quality of life of women with endometriosis and treated in referral centres. Hum Reprod. 2012 May;27(5):1292-9. doi: 10.1093/humrep/des073. Epub 2012 Mar 14.

17. Peters AA, van Dorst E, Jellis B, van Zuuren E, Hermans J, Trimbos JB. A randomized clinical trial to compare two different approaches in women with chronic pelvic pain. Obstet Gynecol. 1991;77(5):740-4.

18. Ball EF, Nur Shafina Muhammad Sharizan E, Franklin G, Rogozinska E. Does mindfulness meditation improve chronic pain? A systematic review. Curr Opin Obstet Gynecol. 2017;29(6):359-66.

19. Kold M, Hansen T, Vedsted-Hansen H, Forman A. Mindfulness-based psychological intervention for coping with pain in endometriosis. Nordic Psychology. 2012;64(1):2-16.

For peer review only - http://bmjopen.bmj.com/site/about/guidelines.xhtml

20. Fox SD FE, Allen RH. Mindfulness meditation for women with chronic pelvic pain: a pilot study. J Reprod Med 2011;56(3-4):158-62.

21. Bishop SR. Mindfulness: A proposed operational definition. Clinical Psychology: Science and Practice; Autumn 2004; 11, 3; Health Module, pg. 230)

22. Plaza I, Demarzo MM, Herrera-Mercadal P, Garcia-Campayo J. Mindfulness-based mobile applications: literature review and analysis of current features. JMIR Mhealth Uhealth. 2013;1(2):e24.

23. Hilton L, Hempel S, Ewing BA, Apaydin E, Xenakis L, Newberry S, et al. Mindfulness Meditation for Chronic Pain: Systematic Review and Meta-analysis. Ann Behav Med. 2017;51(2):199-213.

24. Wang J,Wang Y, Wei C, Yao NA, Yuan A, Shan Y, Yuan C. Smartphone interventions for long-term health management of chronic diseases: an integrative review. Telemed J E Health. 2014 Jun;20(6):570-83. doi: 10.1089/tmj.2013.0243. Epub 2014 May 1.

25. Boudreaux ED, Waring ME, Hayes RB, Sadasivam RS, Mullen S, Pagoto S. Evaluating and selecting mobile health apps: strategies for healthcare providers and healthcare organizations. Transl Behav Med. 2014;4(4):363-71.

26. My Health Apps [Available from: http://myhealthapps.net/app/details/127/Headspace-on-the-go] (Accessed 28 August 2018).

27. Mani M, Kavanagh DJ, Hides L, Stoyanov SR. Review and Evaluation of Mindfulness-Based iPhone Apps. JMIR Mhealth Uhealth. 2015;3(3):e82.

28. App Annie 2018 [Available from: https://www.appannie.com/en/] (Accessed 28 August 2018).

29. Ball E, Newton S, Kahan BC, Forbes G, Wright N, Cantalapiedra Calvete C, et al. Smartphone App Using Mindfulness Meditation for Women With Chronic Pelvic Pain (MEMPHIS): Protocol for a Randomized Feasibility Trial. JMIR Res Protoc. 2018;7(1):e8.

For peer review only - http://bmjopen.bmj.com/site/about/guidelines.xhtml

30. Forbes G, Newton S, Cantalapiedra C, Birch J, Dodds J, Steed E, Rivas C, Khan KS, Rohricht F, Taylor SJC, Kahan B, Ball E. A smartphone app using psychological approaches for women with chronic pelvic pain (MEMPHIS): a randomised feasibility trial. submitted
31. Gerhardt-Powals J. Cognitive engineering principles for enhancing human-computer performance. International Journal of Human-Computer Interaction. 1996;8(2):189-211.
32. Murray E, Treweek S, Pope C, MacFarlane A, Ballini L, Dowrick C, et al. Normalisation process theory: a framework for developing, evaluating and implementing complex interventions. BMC Med. 2010;8:63.
33. Borkan J. Immersion/Crystallization. In BF Crabtree and WL Miller (Eds) Doing Qualitative Research (2nd edition). Thousand Oaks, CA: Sage Publication; 1999. p. pp. 179-94.
34. O'Brien BC, Harris IB, Beckman TJ, Reed DA, Cook DA. Standards for reporting qualitative research: a synthesis of recommendations. Acad Med. 2014;89(9):1245-1251.
35. McCann, S. K., Campbell, M. K., & Entwistle, V. A. (2010). Reasons for participating in randomised controlled trials: conditional altruism and considerations for self. Trials, 11, 31. doi:10.1186/1745-6215-11-31
36. Palmer, M., Sutherland, J., Barnard, S., Wynne, A., Rezel, E., Doel, A., Grigsby-Duffy, L., Edwards, S., Russell, S., Hotopf, E., Perel, P. Free, C. (2018). The effectiveness of smoking cessation, physical activity/diet and alcohol reduction interventions delivered by mobile phones for the prevention of non-communicable diseases: A systematic review of randomised controlled trials. PloS one, 13(1), e0189801. doi:10.1371/journal.pone.0189801
37. Marcolino MS, Oliveira JAQ, D'Agostino M, Ribeiro AL, Alkmim MBM, Novillo-Ortiz D. The Impact of mHealth Interventions: Systematic Review of Systematic Reviews. JMIR Mhealth Uhealth. 2018;6(1):e23.

For peer review only - http://bmjopen.bmj.com/site/about/guidelines.xhtml

38. Laurie J, Blandford A. Making time for mindfulness. Int J Med Inform. 2016;96:38-50.

39. Collins M, Shattell M, Thomas SP. Problematic Interviewee Behaviors in Qualitative Research. Western Journal of Nursing Research. 2016;27(2):188-99.

40. Michie S, Richardson M, Johnston M, Abraham C, Francis J, Hardeman W, et al. The behavior change technique taxonomy (v1) of 93 hierarchically clustered techniques: building an international consensus for the reporting of behavior change interventions. Ann Behav Med. 2013;46(1):81-95.

41. Mohr DC, Cuijpers P, Lehman K. Supportive accountability: a model for providing human support to enhance adherence to eHealth interventions. J Med Internet Res. 2011;13(1):e30.

42. Andersson G. Using the Internet to provide cognitive behaviour therapy. Behav Res Ther. 2009;47(3):175-80.

43. Michie S, van Stralen MM, West R. The behaviour change wheel: a new method for characterising and designing behaviour change interventions. Implement Sci. 2011;6:42.

44. Walsh JC, Corbett T, Hogan M, Duggan J, McNamara A. An mHealth Intervention Using a Smartphone App to Increase Walking Behavior in Young Adults: A Pilot Study. JMIR Mhealth Uhealth. 2016;4(3):e109. Published 2016 Sep 22. doi:10.2196/mhealth.5227

45. Geraghty AW, Torres LD, Leykin Y, Perez-Stable EJ, Munoz RF. Understanding attrition from international Internet health interventions: a step towards global eHealth. Health Promot Int. 2013;28(3):442-52.

46. Mistler LA, Ben-Zeev D, Carpenter-Song E, Brunette MF, Friedman MJ. Mobile Mindfulness Intervention on an Acute Psychiatric Unit: Feasibility and Acceptability Study. JMIR Ment Health. 2017;4(3):e34.

47. Noone C, Hogan MJ. A randomised active-controlled trial to examine the effects of an online mindfulness intervention on executive control, critical thinking and key thinking dispositions in a university student sample. BMC Psychol. 2018;6(1):13.

For peer review only - http://bmjopen.bmj.com/site/about/guidelines.xhtml

48. Wen L, Sweeney TE, Welton L, Trockel M, Katznelson L. Encouraging Mindfulness in Medical House Staff via Smartphone App: A Pilot Study. Acad Psychiatry. 2017;41(5):646-50.

49. Morrison Wylde C, Mahrer NE, Meyer RML, Gold JI. Mindfulness for Novice Pediatric Nurses: Smartphone Application Versus Traditional Intervention. J Pediatr Nurs. 2017;36:205-12.

50. Lim D, Condon P, DeSteno D. Mindfulness and compassion: an examination of mechanism and scalability. PLoS One. 2015;10(2):e0118221.

51. Arean PA, Hallgren KA, Jordan JT, Gazzaley A, Atkins DC, Heagerty PJ, et al. The Use and Effectiveness of Mobile Apps for Depression: Results From a Fully Remote Clinical Trial. J Med Internet Res. 2016;18(12):e330.

52. Gilbody S, Lewis H, Adamson J, Atherton K, Bailey D, Birtwistle J, et al. Effect of Collaborative Care vs Usual Care on Depressive Symptoms in Older Adults With Subthreshold Depression: The CASPER Randomized Clinical Trial. JAMA. 2017;317(7):728-37.

53. Brett J, Staniszewska S, Mockford C, Herron-Marx S, Hughes J, Tysall C, et al. A systematic review of the impact of patient and public involvement on service users, researchers and communities. Patient. 2014;7(4):387-95.

For peer review only

For peer review only - http://bmjopen.bmj.com/site/about/guidelines.xhtml

APPENDIX 1

Feature	Positive aspects	Negative aspects	Solutions
**Voice**	Liked by some participants, considered soothing and even spiritual	The voice was not relaxing. One had to listen carefully which meant you could not switch off; otherwise you could not hear instruction clearly during meditation.<br><br>It was also too repetitive, after 10 times it felt like a chore and not something to look forward to.	Choice of different voices<br><br>participants should be told it takes time to get into the rhythm of the instructions, but you can get used to it
**General interface aesthetics**	colours really fresh, interface not too busy, with pleasing layout and aesthetics	one participant would prefer different, bright, colours such as purple	None needed
**Graphics**		The pictures were often not appropriate as they focused on the problem (e.g. tooth pain logo) rather than something positive or soothing. Unanticipated problems could arise according to one participant; she found it hard to lose weight and felt the food icons a) did not represent success and b) reminded her of eating.<br><br>One participant found the pictures hard to see.	Psychological theory supports the view that positive images would be advisable

For peer review only

**Animations**	liked	poorly chosen	None needed
**Progress**	One participant liked the way the app helped progress through the stages.<br><br>Another participant, with cyclical pain and hence cyclical use, did not aim to progress but simply chose to use the baseline video each time.	Three participants were frustrated and even 'angry' and 'patronised' at having to do the same (basic) meditation several times before moving up a stage.<br><br>There was no indication of goals, duration of each level or gamification rewards.	Goal setting is recommended by psychological theory and could be as simple as using "day 1 of 30" rather than simply Day 1.<br><br>Changing emojis could show progress, for example from a sad face to a smiley face to a heart; the inbuilt progress function was not felt sufficiently motivating.
**Introduction**		Experienced app users suggested the introduction could be improved for initiates	overview of the entire app and its levels suggested for the start.<br><br>One participant had done mindfulness before but thought an introduction to mindfulness might be helpful for others.
**Enjoyment**	considered fun by some participants.	One said it was not very 'interesting'.	Include examples of a patient's day with the app to cater for a greater variety of participants<br><br>It would be good to have other features as drop down options that were fun and

For peer review only

			did not involve meditation.

**Session intensity**		Would be good to choose the duration of the meditation.	15 or 20 mins would be better than 10, which is not enough time shut off and meditate deeply.<br>The option to control this was important as even 10 minutes was a difficult commitment for some busy participants (1074, 1075, 1078) or those battling with chronic fatigue (1074)
**Pain modules**		it can be hard to focus on something like using the app when you are in pain.	None needed

Comments on app acceptability and usability made by n=13 patients. Two patients walked through the app with the researcher

For peer review only

Reporting checklist for qualitative study.

Based on the SRQR guidelines.

Instructions to authors

Complete this checklist by entering the page numbers from your manuscript where readers will find each of the items listed below.

Your article may not currently address all the items on the checklist. Please modify your text to include the missing information. If you are certain that an item does not apply, please write "n/a" and provide a short explanation.

Upload your completed checklist as an extra file when you submit to a journal.

In your methods section, say that you used the SRQR reporting guidelines, and cite them as:

O'Brien BC, Harris IB, Beckman TJ, Reed DA, Cook DA. Standards for reporting qualitative research: a synthesis of recommendations. Acad Med. 2014;89(9):1245-1251.

		Reporting Item	Page Number
	#1	Concise description of the nature and topic of the study identifying the study as qualitative or indicating the approach (e.g. ethnography, grounded theory) or data collection methods (e.g. interview, focus group) is recommended	6
	#2	Summary of the key elements of the study using the abstract format of the intended publication; typically includes background, purpose, methods, results and conclusions	2
Problem formulation	#3	Description and signifcance of the problem / phenomenon studied: review of relevant theory and empirical work; problem statement	4 and 5
Purpose or research question	#4	Purpose of the study and specific objectives or questions	5
Qualitative approach and research paradigm	#5	Qualitative approach (e.g. ethnography, grounded theory, case study, phenomenolgy, narrative research) and guiding theory if appropriate; identifying the research paradigm (e.g. postpositivist, constructivist / interpretivist) is also recommended; rationale. The	7

For peer review only

		rationale should briefly discuss the justification for choosing that theory, approach, method or technique rather than other options available; the assumptions and limitations implicit in those choices and how those choices influence study conclusions and transferability. As appropriate the rationale for several items might be discussed together.	
Researcher characteristics and reflexivity	#6	Researchers' characteristics that may influence the research, including personal attributes, qualifications / experience, relationship with participants, assumptions and / or presuppositions; potential or actual interaction between researchers' characteristics and the research questions, approach, methods, results and / or transferability	19
Context	#7	Setting / site and salient contextual factors; rationale	7
Sampling strategy	#8	How and why research participants, documents, or events were selected; criteria for deciding when no further sampling was necessary (e.g. sampling saturation); rationale	7
Ethical issues pertaining to human subjects	#9	Documentation of approval by an appropriate ethics review board and participant consent, or explanation for lack thereof; other confidentiality and data security issues	6
Data collection methods	#10	Types of data collected; details of data collection procedures including (as appropriate) start and stop dates of data collection and analysis, iterative process, triangulation of sources / methods, and modification of procedures in response to evolving study findings; rationale	7
Data collection instruments and technologies	#11	Description of instruments (e.g. interview guides, questionnaires) and devices (e.g. audio recorders) used for data collection; if / how the instruments(s) changed over the course of the study	8
Units of study	#12	Number and relevant characteristics of participants, documents, or events included in the study; level of participation (could be reported in results)	See note 1
Data processing	#13	Methods for processing data prior to and during analysis, including transcription, data entry, data management and security, verification of data integrity, data coding, and anonymisation / deidentification of excerpts	8
Data analysis	#14	Process by which inferences, themes, etc. were identified and	8

For peer review only

		developed, including the researchers involved in data analysis; usually references a specific paradigm or approach; rationale	
Techniques to enhance trustworthiness	#15	Techniques to enhance trustworthiness and credibility of data analysis (e.g. member checking, audit trail, triangulation); rationale	8
Syntheses and interpretation	#16	Main findings (e.g. interpretations, inferences, and themes); might include development of a theory or model, or integration with prior research or theory	8-16
Links to empirical data	#17	Evidence (e.g. quotes, field notes, text excerpts, photographs) to substantiate analytic findings	See note 2
Intergration with prior work, implications, transferability and contribution(s) to the field	#18	Short summary of main findings; explanation of how findings and conclusions connect to, support, elaborate on, or challenge conclusions of earlier scholarship; discussion of scope of application / generalizability; identification of unique contributions(s) to scholarship in a discipline or field	16
Limitations	#19	Trustworthiness and limitations of findings	19
Conflicts of interest	#20	Potential sources of influence of perceived influence on study conduct and conclusions; how these were managed	4
Funding	#21	Sources of funding and other support; role of funders in data collection, interpretation and reporting	3

Author notes

1. 7,8,9 and especially 10

2. 8-16 and appendix

The SRQR checklist is distributed with permission of Wolters Kluwer © 2014 by the Association of American Medical Colleges. This checklist was completed on 26. March 2019 using https://www.goodreports.org/, a tool made by the EQUATOR Network in collaboration with Penelope.ai

For peer review only

BMJ Open

Mhealth – Providing a Mindfulness App for women with chronic pelvic pain in gynaecology outpatient clinics: Qualitative data analysis of user experience and lessons learnt

Journal:	*BMJ Open*
Manuscript ID	bmjopen-2019-030711.R3
Article Type:	Original research
Date Submitted by the Author:	04-Dec-2019

Complete List of Authors:	Ball, ELizabeth; Queen Mary University of London - Whitechapel Campus, Yvonne Carter Building<br>Newton, Sian; QMUL<br>Rohricht, Frank ; East London NHS Foundation Trust<br>Steed, Liz; QMUL<br>Birch, Judy<br>Dodds, Julie; QMUL, Women'shealth Research Unit<br>Cantalapiedra Calvete, Clara; QMUL, Women's Health Research Unit<br>Taylor, Stephanie; QMUL, Center for Primary Care and Population Health<br>Rivas, Carol; University College London

**Primary Subject Heading**:	Obstetrics and gynaecology
Secondary Subject Heading:	Health informatics
Keywords:	Chronic pelvic pain, mHealth, mindfulness, patient engagement, health app, feasibility study

SCHOLARONE™
Manuscripts

I, the Submitting Author has the right to grant and does grant on behalf of all authors of the Work (as defined in the below author licence), an exclusive licence and/or a non-exclusive licence for contributions from authors who are: i) UK Crown employees; ii) where BMJ has agreed a CC-BY licence shall apply, and/or iii) in accordance with the terms applicable for US Federal Government officers or employees acting as part of their official duties; on a worldwide, perpetual, irrevocable, royalty-free basis to BMJ Publishing Group Ltd ("BMJ") its licensees and where the relevant Journal is co-owned by BMJ to the co-owners of the Journal, to publish the Work in this journal and any other BMJ products and to exploit all rights, as set out in our licence.

The Submitting Author accepts and understands that any supply made under these terms is made by BMJ to the Submitting Author unless you are acting as an employee on behalf of your employer or a postgraduate student of an affiliated institution which is paying any applicable article publishing charge ("APC") for Open Access articles. Where the Submitting Author wishes to make the Work available on an Open Access basis (and intends to pay the relevant APC), the terms of reuse of such Open Access shall be governed by a Creative Commons licence – details of these licences and which Creative Commons licence will apply to this Work are set out in our licence referred to above.

Other than as permitted in any relevant BMJ Author's Self Archiving Policies, I confirm this Work has not been accepted for publication elsewhere, is not being considered for publication elsewhere and does not duplicate material already published. I confirm all authors consent to publication of this Work and authorise the granting of this licence.

For peer review only

Mhealth – Providing a Mindfulness App for women with chronic pelvic pain in gynaecology outpatient clinics: Qualitative data analysis of user experience and lessons learnt

Ball, Elizabeth
Department of Obstetrics and Gynaecology, Barts Health NHS Trust, United Kingdom, Women's Health Research Unit, Barts and the London School of Medicine and Dentistry, Queen Mary University of London, United Kingdom, Centre for Maternal and child Health Research, City University London

Newton, Sian
Centre for Primary Care and Population Health, Queen Mary University of London, United Kingdom

Rohricht, Frank
East London NHS Foundation Trust, United Kingdom

Steed, Liz
Centre for Primary Care and Population Health, Queen Mary University of London, United Kingdom

Birch, Judy
Pelvic Pain Support Network, UK

Dodds, Julie
Women's Health Research Unit, Barts and the London School of Medicine and Dentistry, Queen Mary University of London, United Kingdom

Cantalapiedra Calvete, Clara
Department of Obstetrics and Gynaecology, Barts Health NHS Trust, United Kingdom

Taylor, Stephanie JC
Centre for Primary Care and Population Health, Queen Mary University of London, United Kingdom

Rivas, Carol
Department of Social Science, University College London, United Kingdom

Corresponding author

Carol Rivas, Department of Social Science, University College London, United Kingdom
E-mail: c.rivas@ucl.ac.uk

For peer review only

Abstract

Objective

To determine whether a pre-existing smartphone App to teach Mindfulness Meditation is acceptable to women with chronic pelvic pain (CPP) and can be integrated into clinical practice within NHS CPP pathways. To inform the design of a potential randomised clinical trial.

Design

A pre-study patient and public involvement (PPI) group to collect feedback on the acceptability of the existing app and study design was followed by a three-arm randomised feasibility trial. In addition, we undertook interviews and focus groups with patients and staff to explore app usability and acceptability. We also obtained participant comments on the research process, such as acceptability of the study questionnaires.

Setting

Two gynaecology clinics within Barts Health NHS, London, UK.

Participants

Patients with CPP lasting ≥6 months with access to smartphone or PC and understanding of basic English.

Intervention

The intervention was mindfulness meditation content plus additional pain module delivered by smartphone app, active controls received muscle relaxation content by the same app. Passive (waiting list) controls received usual care.

Main outcome measures

Themes on user feedback, app usability and integration and reasons for using/not using the app.

Results

App use was low in both active groups.

Patients in the pre-study PPI group, all volunteers, were enthusiastic about the app (convenience, content, portability, flexibility, ease of use). Women contributing to the

For peer review only - http://bmjopen.bmj.com/site/about/guidelines.xhtml

interview or focus group data (n=14), from a ‘real world’ clinic, (some not regular app users) were less positive, citing as barriers lack of opportunities/motivation to use the app, and lack of familiarity and capabilities with technology. Staff (n=7) were concerned about the potential need for extra support for patients and staff and considered the app needed organisational backing and peer acceptance.

Conclusion

The opinions of pre-study PPI volunteers meeting in their private time may not represent those of patients recruited at a routine clinic appointment.

It may be more successful to co-design/co-develop an app with typical users than to adapt existing apps for use in real-world clinical populations.

Trial registration and funding

The trial (ISRCTN 10925965) was funded by the UK National Institute of Health Research, Research for Patient Benefit programme (RfPB PB-PG-1013-32025).

Keywords

Chronic pelvic pain, mHealth, mindfulness, Headspace, PPI, patient engagement, feasibility study, health app

For peer review only

Article Summary: 'Strengths and limitations of this study'

- The study was designed with the help of a study design group of CPP patients
- Patient recruitment to the study was good
- Unusually our study focused on a deprived urban area of the UK and considered typical local clinical patients
- The qualitative evaluation included the perspectives of both patients and a variety of healthcare staff
- Patients in the qualitative evaluation preferred telephone interviews over the offered face to face focus groups.

The original protocol for the study has been published separately. The UK National Institute of Health Research supported this work (RfPB PB-PG-1013-32025). There are no competing interests.

Introduction

Smartphone health apps, as one form of mhealth (1), are popular in the UK, our study setting. With more than two-thirds of the UK population using smartphones (2,3,4), health apps are one of the fastest growing app categories, thus numbers of users are still increasing (5). Currently these apps are usually developed either by researchers or (in the majority) by commercial companies, without collaboration between these groups (6,7). The lack of interaction between researchers and commercial developers in the field of pain-related apps has led to a situation where commercially available apps have not been scientifically validated and apps that have been developed from research projects are not commercially available (8).

For peer review only - http://bmjopen.bmj.com/site/about/guidelines.xhtml

We were interested in using an app to support women with chronic pelvic pain (CPP) in a clinical setting, where validation of an intervention is important to ensure best care. CPP is defined as a subjective physical and emotional experience of pain in the pelvic area that has been present for at least six months that may or may not have an identifiable pathology (9). CPP affects up to 24% of women worldwide (10) and accounts for 20% of gynaecological clinic referrals. (11,12) It has considerable impact on patients' quality of life, including their mental health and their income (13) due to loss of working days and diminished work capacity. Annual costs to the NHS have been estimated at approximately £326 million (14). For endometriosis alone, which is just one cause of CPP, a European study of over 900 women showed average annual total costs per woman of €9579. Costs of productivity loss of €6298 were double the health care costs of €3113 per woman. The latter were due to surgery (29%), monitoring tests (19%) and hospitalization (18%) and physician visits (16%) (15).

Despite costly interventions, CPP is often resistant to surgical and medical treatment and appears to respond better to a multimodal, holistic approach, (16) with a focus on coping strategies. A systematic review of randomised controlled trials (RCTs) by authors has identified mindfulness meditation (MM) as an effective coping strategy in other chronic pain conditions (17). In addition, evidence from uncontrolled trials suggests positive effects of MM for CPP, such as an increased ability to control pain, improvements in mental health, emotional well-being, work and family life and social functioning (18,19), but these have never been examined in an RCT.

We therefore chose to evaluate MM delivered via an app to women with CPP as our intervention. CPP is especially common in younger women, who may be categorised as digital natives, making an app-based intervention particularly appropriate in this group.

In MM the aim is to keep focussed on one's own breathing. Whenever attention wanders to intrusive thoughts and feelings these are simply taken notice of in a neutral way, that is,

For peer review only - http://bmjopen.bmj.com/site/about/guidelines.xhtml

without elaboration or judgements or consideration of action. They are then let go as attention is returned to the breath. The idea is to bring awareness back to the here and now whenever worries and troubles intrude into thoughts.(20)

Generally two main complementary approaches have been used for MM: 1) exercises focusing one's attention to the present moment, and 2) monitoring of experiences in the present moment. While systematic reviews show that MM may have positive effects on depression, quality of life and pain symptoms in patients with chronic pain (17,21,22) and apps with such a focus on chronic disease have been shown to be beneficial in various conditions (23) none of the reviewed papers included meditation delivered via mobile phone apps or in women with CPP.

Evaluation of an existing app is often appropriate (24) and is both quicker and more cost-effective than designing an app from scratch. We chose to evaluate an existing commercial app platform that teaches mindfulness by guided meditation (Headspace ®), with a ten day basic meditation module followed by a pain module specifically designed for the MEMPHIS (Mindfulness meditation using a smart-phone application for women with chronic pelvic pain) study. The Headspace app was publicly nominated favourite health app of 2013,(25) has a 5 star user rating in the Apple™ app shop and has scored top in a systematic review of 23 mindfulness apps using the Mobile Application Rating Scale (visual aesthetics, engagement, functionality or information quality) (26). Headspace had reportedly seen over 15 million downloads up to mid-2018 when our study began.(27) To our knowledge the Headspace app in its original or modified form has not been assessed in any other pain conditions.

We undertook a three-arm parallel randomised feasibility trial (MEMPHIS) (28) to assess whether or not to proceed with a full randomised controlled trial of the modified Headspace meditation app for women with CPP. In the current paper we report on the qualitative interview and focus group data from this study; the protocol and quantitative results have been

For peer review only - http://bmjopen.bmj.com/site/about/guidelines.xhtml

published/ submitted (29,30). Our aim for the qualitative part of this study was to determine whether a pre-existing smartphone app to teach MM is acceptable to women with CPP and can be integrated into clinical practice within NHS CPP pathways. Objectives were to consider:

1) The acceptability, use and usability of the app in the intended service user population and for health care professionals (doctors, health care assistants, clinical and research nurses)

2) The feasibility of integrating such an app into existing healthcare pathways

3) The usefulness of having a distinct patient group to advise us on the study design.

Methods

Outcomes

The outcomes of this analysis were inductively derived descriptive themes on acceptability, use and usability of the app and feasibility of integrating it into existing pathways. We follow the ISO 9241-11 (https://www.iso.org/obp/ui/#iso:std:iso:9241:-11:ed-2:v1:en) concept of technology usability (user friendliness) as the extent to which the app could be satisfactorily used by participants to meditate. By acceptability we mean whether participants could see a reason for using the app when given in clinic, and would be happy to use it for meditation.

Intervention Procedures

Women in the mindfulness meditation group received access to a 60-day progressive mindfulness meditation course delivered via the Headspace app. The first 10 days of the course taught basics of mindfulness meditation. Following this, participants were able to access the module on meditation which was targeted at for chronic pain. This module had

For peer review only - http://bmjopen.bmj.com/site/about/guidelines.xhtml

been specifically made for this study. Session length was 10 minutes for the first 10 days, 15 min up to day 20 and 20 min up to day 60. The active control group received access to a series of muscle relaxation sessions. These sessions were identical every day, except that their duration increased to mirror the increasing duration of the meditation content being listened to by the intervention group. Usage data are reported elsewhere (30).

Patient and Public Involvement

We held a Patient and Public Involvement (PPI) group workshop before the study to discuss acceptability of the Headspace app and help us design our study. Women attending the Royal London Hospital CPP clinic were invited to volunteer for a week of using the unmodified (normal commercially available) Headspace app (which did not have the pain module at the time we undertook our pre study workshop) and then feed back on their experiences with the app in an evening discussion group. Women were not involved in the design of the modified app. The focus for the PPI group was on the use of the generic MM app.

Two patient representatives provided support from the study design stage through recruitment to the interpretation of the results and regularly attended Trial Management Group meetings.

For peer review only

Study recruitment and eligibility

The trial recruited at two outpatient gynaecology clinics within Barts Health NHS trust in two separate deprived areas of inner East London. Female patients with new or follow-up gynaecology appointments were assessed for eligibility by a researcher in clinic, having been posted a Patient Information Sheet. Women were eligible if they had been suffering with CPP for 6 months or more and had at least a basic understanding of the English language, sufficient to follow instructions, as assessed during discussion about the study for

For peer review only - http://bmjopen.bmj.com/site/about/guidelines.xhtml

informed consent; no women were excluded on this basis. Women were excluded if they did not meet these criteria or they did not have access to a smartphone or personal computer or were currently using the Headspace app (there were very few of the latter, according to the impression of the recruiting nurses). All patients gave full and informed consent to be randomised and data were collected through all stages of the study. All healthcare professionals and research nurses involved in the two clinics were also invited to take part in the feasibility study as the only eligibility criterion for staff. Full enrolment data are provided in Forbes et al (30). A key difference of these patients from those in the PPI group was that their focus was on managing their pain, with the app given explicitly as part of their clinic management support.

For the study of quantitative data, 90 patients were allocated randomly in a 1:1:1 ratio to the mindfulness meditation app, a muscle relaxation app active control or the usual care arm (for full details see (29)). Patients in the two active arms were asked to download the modified app in the clinic with support from a research staff member and were sent a questionnaire about app usability, an analysis of which is reported in a companion paper.(30) We used data from the app usability questionnaire to inform topic guides for the qualitative part of the study. This outlined key usability issues that had been uncovered, to guide our semi-structured interviews and focus groups with patients and staff.

All women in the intervention and active control arms were eligible for the qualitative component of the study, and all staff participating in the study.

Within-study interviews and focus groups

All patients in the intervention and active control arms were invited to one of two focus groups at their own study site after the 6-month follow up. We offered telephone interviews as an alternative. Patients were asked to 'walk through' the app with researchers, articulating their thought processes while they did so and commenting on its different specific features,

For peer review only - http://bmjopen.bmj.com/site/about/guidelines.xhtml

(31). Walkthroughs are often used in developing technologies such as mhealth. This helped to identify app usability issues or barriers to use of the app from the users' points of view without the need for technical discussions. Results for the walkthrough, showing comments on different features specific to the usability of the intervention app used in our study are shown in Appendix 1; walkthroughs were undertaken by two patients. Patients also discussed with us their experiences around app usability and acceptability.

Staff were invited to attend a staff focus group overseen by the patient representative and facilitated by a researcher. In addition to considering app usability and acceptability, members of the staff focus group (consultants, health care assistants, clinical and research nurses and a representative from the pelvic pain support network were eligible) were asked about the ease of integration into existing NHS pathways. Part of the staff discussion was free flowing with open-ended questions, which gave us patient-focussed information on app acceptability, and part was structured using questions developed from the Normalisation Process Theory (NPT) toolkit in the way recommended by the NPT developers (32). For example, we asked whether staff could see a purpose to the app in clinical practice, as adding something different, which corresponds to the NPT toolkit question 'Participants distinguish the intervention from current ways of working'. Since this was a semi-structured approach questions were not rigidly worded. This helped us to consider the feasibility of integration of the app into practice. NPT is a theory of implementation practices that was initially developed for consideration of technology implementation and is in common use (32).

All data were audio-recorded at point of collection and transcribed, with personal identifying data removed from transcripts. Raw data were stored in a Primary Care Clinical Trial Unit database to clinical trial standards.

Analysis

For peer review only - http://bmjopen.bmj.com/site/about/guidelines.xhtml

Analysis of within-study focus groups and interviews was carried out blinded as to which study app was used, and deployed the immersion-crystallisation method (33). Thus, the lead qualitative researcher immersed herself in the data, reading transcripts carefully, then writing down articulated or crystallised patterns or themes that related to the aims and research questions of the study. These were discussed with another researcher from the team, and themes modified as appropriate. This process was repeated until all the data had been examined and all patterns that had been noticed were articulated, discussed and substantiated with exemplar extracts. This approach was considered appropriate since we had a small dataset and we were not aiming to develop conceptual themes but rather to inform the design and development of a randomised controlled trial for the modified app. We used the SRQR checklist when writing our report (34).

Ethics

The MEMPHIS trial was a three-arm parallel randomised feasibility trial approved by Camden and Kings Cross Research Ethics Committee in 2016 (15/LO/1967).

Results

We screened 488 women between May and September 2016 for their eligibility to participate in the study. After exclusions, 90 women gave full consent to participate and were randomised to the intervention arm (31 women), the active control arm (30 women) or the usual care arm (29 women).

Demographics

Women in the main feasibility trial (30) were aged a mean of 35 years, 66% were employed and overall approximately 50% had stayed in full time education until at least the age of 20 though the proportion was least, at 36.7% in the intervention arm. Overall 44% were of white ethnicity though the proportion was lowest in the intervention arm (35.7%) and highest in the usual care arm (53.6%). The second most common self-reported ethnic groups were

For peer review only - http://bmjopen.bmj.com/site/about/guidelines.xhtml

'Southern Asian' and 'Black'. Women in the intervention arm were most likely to have experienced CPP for 3-5 years (40.3% of this group), women in the usual care group for over 10 years (42.9% of this group). More women had pain for longer than two years in the intervention arm than in either of the other two arms. All women reported a high pain intensity, with means of 6.8 to 6.9 in the previous week (on a scale from 1-10) (30). These and other demographic data are reported in more detail in Forbes *et al*. (30) Our qualitative sample was taken from the two active arms and was comprised of 16% of trial participants and 23% of those eligible for the qualitative study. We did not record separate demographic data for the women in this smaller sample.

For peer review only

For peer review only - http://bmjopen.bmj.com/site/about/guidelines.xhtml

Pre-study PPI group

The ten women in the pre-study PPI group were self-selected local women who were familiar with using apps and focused on app use per se. They anticipated no technical issues even in women who were not used to apps. They considered that the Headspace app would be successfully adopted by patients taking part in the study, given that a smartphone, like CPP, is 'always with you'. They praised the flexibility of the app, welcomed its portability and were unanimous in saying it was easy to slip off for 10 minutes when at work to use it. As a result, they found they could use it at times when they most needed pain relief as well as to prevent pain and found the app helpful in relieving pain and stress. The group reported being able to meditate without the app, once they had tried it with the app; however, they still preferred to use the app because they found the voice soothing.

App usage in the study

Patient usage of the app was less than expected from our pre-study PPI group discussions. Only 36% of meditation app patients and 46% of the active control patients used the app at least once. (30)

Thematic analysis of within-study data

Qualitative data were obtained from 14 study patients; 12 preferred a telephone interview, two attended face-to-face interviews, one participant at the University attached to one of the recruiting clinics and one participant at the other recruiting hospital. Patients chose not to attend focus groups. Four of the patients were from the active control arm and 10 from the intervention arm. The two women we met face to face had both used the intervention and neither had progressed beyond the training stage, something that we cannot discount for other participants and which may help to explain reports of lack of effect on pain. Seven people attended the staff focus group: two recruiting nurses, three clinic nurses one consultant and a representative from the pelvic pain support network.

For peer review only - http://bmjopen.bmj.com/site/about/guidelines.xhtml

The qualitative analysis revealed three main themes from all within-study interviews and focus groups combined regarding usability, and four subthemes:

1. Familiarity and capabilities with app technology
2. Motivations to use the app
   - Perceived benefits
   - Relation to other therapies
3. Opportunities to use the app:
   - Technology issues getting in the way
   - Life getting in the way

These are explored below. As the PPI group data were not research data we did not analyse them for themes.

While we initially combined active control and intervention groups in our analysis, we then looked for instances where there was a difference between these two groups. Only where we found this difference in any theme or statement have we specified which group women belonged to.

Familiarity and capabilities with app technology

Around half of the patients were sufficiently familiar with technology and apps to be comfortable using the study apps. However, six participants (all using the intervention, which was more complex than the active control) reported difficulty because they were *"not very good at technology*" (patient 1002, intervention), or were unsure how to get started or use the app effectively without help.

> *I am not good with technical some things that is why the problems I had arisen, right okay. So I consulted with my daughter and she helped me work it out… so I don't try everything. (patient 1002, intervention)*

One further patient *(1001, intervention)* was not used to technical app language; 'help' suggested emotional support to her, for example. Two more (one intervention, one active

control) changed handsets and therefore did not continue with the app. In all cases these technical difficulties appeared to lead to abandoning of the app or restricted use of its functionality.

Five patients having technical problems suggested possible solutions such as a ‘class’ or group for first time users, a YouTube orientation video, or a pictorial leaflet. This might include an introduction to meditation and mindfulness as well as the app itself. One woman commented: *“If your market is targeting people who are not using apps then you are going to have to get together and find ways to do this”,* (patient 1041, intervention); she also suggested we could get ideas from other apps on the market in this regard.

Given their experiences in the study, staff were concerned about additional staff time needed to support women in using the app. This would sit in tension with one of the original rationales behind choosing an app as the mode of delivery, which was to increase the effective use of staff contact time with patients. Language barriers might compound problems.

Motivations to use the app

Staff, though unaware of the low sustained app use in the study, felt it would be common sense to hold occasional motivating meetings with patients if the intention was for them to use the app long term. The patient data suggested the main motivators or lack of motivation for using the app in our sample, which could be drawn on in such meetings, and which we now consider.

<u>Perceived benefits</u>

Three intervention arm patients said they only entered the trial to help others through research but were already using alternative forms of pain control. They explained that this meant they were not motivated to actually use the app, perceiving the relative benefit to be

For peer review only - http://bmjopen.bmj.com/site/about/guidelines.xhtml

small. The failure of such altruism to extend to using the app is a recognised phenomenon in clinical trials and has been called ‘weak altruism’. (35) Thus, only one of these three patients persevered. Even though she was one of the women who experienced difficulties with the technology, she explained, “*with something that is as soul destroying as the pain, it is important to help others off the back of other people's misfortune as it were”* (1036, intervention). However, she wondered how relevant her data were:

I took steps to improve my situation from a weight loss perspective as well and I’ve lost a lot of weight which has significantly helped not 100% but it is has significantly helped so I felt a bit fraudulent the last time filling in the forms because , so everything had improved so much so from the medical study perspective it was more about the weight loss than the app. So I felt a bit bad that I was still taking part. (patient 1036)

There was no clear pattern regarding the impact of current pain on app use by patients. Six said they used it regardless of pain intensity – sometimes developing a daily routine - while four only used it when in severe pain or expecting to be (e.g. during menstruation). This cyclical or intermittent use in some patients – which was irrespective of study arm - should be considered when looking at our main study outcomes.(30) The Headspace app requires regular use to learn and benefit from psychological techniques. To address this, healthcare professional alerts have been effective in other studies, (36) whilst Headspace only has a reminder function that the user can set. This was often not sufficient, as one patient said even with this feature, “*To be quite honest I used it a couple of times and then forgot. And then I [remembered it and] used it more frequently.” (patient 1036, intervention)*

One patient said medication was not working but the app did, though she was not sure whether this was ‘*mind over matter’* (1065, intervention), which was her term for a placebo effect. Three others said it did not reduce their pain; all three were using the intervention app. The remaining patients considered other benefits were good reason for using the app even when they did not feel that it reduced pain intensity. Alternative or unanticipated benefits were not formally measured or taken into account in the study’s effectiveness

For peer review only - http://bmjopen.bmj.com/site/about/guidelines.xhtml

outcomes (30). For example, ten patients valued the way the app helped them to relax or de-stress or focus and re-assess their life; three of these specifically said they used it to induce a relaxed state to get to sleep. Notably the active control was a relaxation app; however this benefit was also reported by many women in the intervention arm. One participant (active control) said she did not like the focus on pain per se as her condition impacted on various areas of her life. Even when the intervention app was positively received, women might stop using it because it was too powerful, and they had gained the change they wanted:

I think it was day 3, I could see the change that was happening, I was able to speak up for myself ……I can't explain it, even now I am getting emotional… it's just a lack of focus, I just needed direction. To try and put It into words. To me it meant so much that I have gone back to church… I use it outside of the app now I have got from it what was missing, so it's done something to me and for me which is very positive, and I may try it to lose weight but those positive vibes are still there. I can't go back to it because I did not want to go any further because what I got at the time helped me to focus, to change my way of thinking. I used it for about two or three weeks. (patient 1001, intervention)

Three intervention app patients found the app put them more in tune with their bodies and their breathing, (two of these were among those who also found the app de-stressing) while another found yoga better for that. Six patients, like pre-study PPI group members, also learned to use techniques from the app to alter their stress patterns without the app, having tried it, for example in traffic or by sitting down and taking time out or for general relaxation.

Relation to other therapies

Two patients (both active control) preferred 'pure' meditation, another considered the app to be "*very much about meditatio*n" (1041, intervention), which is in keeping with the arms they were in. An alternative therapy practitioner and two further patients reported that they preferred yoga. One (active control) said this was because it focussed on each part of the body in turn.

For peer review only - http://bmjopen.bmj.com/site/about/guidelines.xhtml

Three intervention app patients thought the app was useful as an adjunct to other methods rather than a replacement for them, for example physical interventions such as Pilates, or listening to classical music.

Opportunities to use the app

Technology issues getting in the way

Staff pointed out that not all patients had smartphones (not appreciating that PCs/tablets were alternatives allowed in this study). Some patients lacked the storage space to load the app on their phones. There were also issues with Wi-Fi connectivity when staff tried to help the patients load the app within the hospital sites. Possible solutions that staff suggested were to lend patients phones and to have group upload sessions in a location with good Wi-Fi signal – though they acknowledged the resource implications.

Life getting in the way

Seven patients revealed they preferred to use the app in the evenings because of other life commitments. This meant they did not always use it as a direct response to pain, reducing its potential for contemporaneous effect. One patient who used it in response to pain but only used it once or twice blamed this on having no spare time because of juggling work and children (active control); however, another patient (intervention) managed despite such commitments the fact that she was in the intervention arm may have played a role.

Barriers to integration for staff

Staff believed that the biggest barrier to clinical adoption of the app was a possible lack of support from the host organisation. It might also be hard to integrate the app within existing professional work practices if the staff in the position of offering the app to patients failed to see its relative advantage over other interventions. Collection of feedback on the app's effectiveness would be necessary for staff to support sustained use. It was felt that staff would need training on how to introduce the app to women in practice, and that complexity

For peer review only - http://bmjopen.bmj.com/site/about/guidelines.xhtml

and high staff turnover could impede sustained use. An app was also seen as impersonal compared with face-to-face contact, which was more favoured by staff.

Participant comments on the research process

The study questionnaires that were used for the main quantitative outcome measures (30) were acceptable to patients except for some discomfort with a question about sex, which patients considered a delicate question that was missing a 'no sex' option. Most preferred a paper form reflecting their lack of affinity with technology. There were no indications that the study design or study processes had contributed to the participants' lack of engagement with the apps – with a caveat around support with the technology as mentioned above - though we did not systematically consider this. A full summary of patient comments on the study design and procedures is given in Appendix 1.

Discussion

Our study adds to the limited evidence on mHealth app user behaviour and experience (36,37). The pre-study PPI group (young women, of a generation who were familiar with using apps and who were asked to focus on the study design use of the app) liked the idea of delivering the intervention via an app, praising the contemporary design and flexibility. Hence we expected a similar positive attitude from trial participants, who were of a comparable age, and we assumed would be keen on using apps. Participant feedback revealed that this assumption was too simplistic.

Using our qualitative data, we were able to explore reasons for low app usage that had been recorded in our feasibility study.(30) Our thematic analysis suggests that the low app use in the trial occurred because many patients were not familiar with apps in general or lacked capabilities with technology. This was particularly true for the more complex intervention app. The other themes we report did not differ between groups (although the three cases of weak altruism' all occurred in the intervention arm) which suggests more generic issues that

For peer review only - http://bmjopen.bmj.com/site/about/guidelines.xhtml

can be transferable to other app use situations. For example, women stated limited motivation to use the app because of a lack of perceived benefit, or a lack of opportunity to use the app due to Wi-Fi issues or due to other commitments.

Similar findings were reported by Laurie *et al*.(38) who interviewed 16 healthy city-dwelling participants (25-38 years) about their user behaviour before and after 30-40 days of Headspace app exposure. Like us, they reported barriers of busy lives, failure to establish a routine and a lack of perceived benefit; all users in their study tried the app at least once hoping it could deliver a quick fix but were disappointed if this did not happen. In our study many patients failed to perceive a benefit from using the app. Hence excuses stating other commitments may mask a deeper lack of motivation linked to perceptions of benefits.(39)

The advantages and disadvantages of using the app stand-alone were also illustrated by our data. Some suggestions made by participants to improve usage, such as more guidance at the start, seem obvious in hindsight. But they had not been considered because of the feedback from the pre-study PPI group and the commercial success of Headspace. The use of community contacts may be a helpful alternative.(40) Social support can create a community of practice, help to clarify expectations,(41) and improve health outcomes (as shown for example in internet based psychological treatment for depression (42).

The data suggest that for successful app use we need to understand what motivates individuals with clinical need to use the app for clinical reasons (which our PPI group did not focus on) and target this, for example by setting appropriate expectations. Incentivisation might also improve motivation. This could be achieved through app gamification (7), or encouragement through integration with patient-clinician face-to-face encounters, which was lacking in our study since the app was used stand-alone. The present study provided extensive initial technical support but no coaching and incentivising, in keeping with the protocol. Future app studies should take this into account. Participants in our study may

For peer review only - http://bmjopen.bmj.com/site/about/guidelines.xhtml

have also have benefitted from training and support to improve their app use capabilities and guidance on how to create more opportunities for app use – such as through sharing experiences in clinic support groups. This is in keeping with the COM-B model of behaviour change (43) which our themes matched, though this was only realised after analysis. The COM-B model says that Capability, Opportunity and Motivation are key drivers of behaviour and has been used to develop a number of complex interventions including smartphone apps (e.g. 3).

Lack of engagement after recruitment, or good initial engagement but minimal or inconsistent use, have been reported in other studies (44,45), including in Headspace trials other than our own (46, 47). Settings were a university and a psychiatric inpatients clinic, both in the USA. Inconsistent app use was noted by Wen (48) in junior doctors who used self-guided Headspace. Morrison Wylde (49) compared face-to-face MM with headspace use in novice paediatric nurses. However, unlike our study there were no recorded dropouts/non-users and also no record of whether or how long the app was used for which is an important omission.

None of these studies included a qualitative component. Yet, each of the Headspace study groups were very different, and so will likely have differed in motivations, contexts for opportunity to use the app, and incentivisations (50). While these aspects were not considered in the other studies, our use of qualitative research has enabled us to explore these in more depth. Our findings suggest these are important considerations in any study of app use and therefore this study makes a contribution to the field. For example, some of the groups in other studies may have differed from ours in likelihood of using mhealth apps in the first place, and familiarity with technology. In-patients may have more time to use the app and more support – and may also have had specific barriers to app use, such as related to setting and to illness.

For peer review only

Patients in the qualitative part of our study tended not to use apps on a regular basis (or at least apps other than simple games), and in terms of our themes, also represented in the COM-B model, may be said to have few capabilities in technology use. They therefore do not represent the typical users of the Headspace app in a commercial setting. Accessing the app regularly requires energy, time and effort, but patients with CPP often suffer from fatigue and anxiety as co-morbidities, perhaps whilst having to juggle family life and work. Therefore, this may be seen as a challenging clinical population in which to trial an app. Further Headspace trials with diabetic (NCT03274362) and pain (NCT03495726) outpatients are underway.

Our study has also shown that clear consideration of unexpected benefits should be included in future studies and these can be informed by our finding that benefits for patients may be more diffuse than anticipated (e.g. app relieving stress rather than pain). Such benefits were found in the active control as well as the intervention arm and so it may be that they represent a placebo effect though the effect could equally be real. Our data also suggest that staff benefits may be less than anticipated, as participants sometimes needed a lot of support and scaffolding in technology use at least initially.

Young age, co-morbid anxiety and low educational attainment are predictors for dropping out of web-based interventions according to studies in the field of depression. (51, 52) This may be true despite regular phone support (52) though our participants all considered active motivational support from staff or app support groups would have improved app use. Our intervention arm participants were particularly likely to be young and with low educational attainment.

Our data suggest that it is important to involve real world end users in the agile design or development or modification of apps in close collaboration with researchers and commercial app developers.(7) Although the evaluation of existing apps has been recommended as a

For peer review only - http://bmjopen.bmj.com/site/about/guidelines.xhtml

cost-effective and rapid process, (24) our findings suggest that in actual clinical practice these may be problematic.

Strengths and weaknesses of the study and in relation to other studies

One strength of this study is that it creates much-needed evidence in the field of evaluating existing health apps in a clinic population (6,8,24) and recording user experience. This provides us with lessons to be learned.

Researchers conducting interviews and focus groups were: a senior mixed methods medical sociology researcher, a recruiting nurse, a representative from the pelvic pain support network and an experienced health psychologist. Findings were similar across the data and the different backgrounds of the researchers therefore does not appear to have influenced findings. The main analysis was undertaken by the medical sociologist and so the concordance with the COM-B model is not due to background discipline bias.

We were able to recruit successfully, and we obtained valuable information from patients with CPP, who were recruited from a deprived urban area of the UK as typical local clinical patients.

However, we report a marked discrepancy between the attitudes of the pre-study PPI group of volunteer patients from the local area, who actively put themselves forward for a 7-day trial of the app, and the participants asked to take part when they attended clinics. The opinions of pre-study PPI volunteers meeting in their private time may not be representative of the opinions of patients recruited at a routine clinic appointment. Women in the PPI group were used to using apps, which had led them to be interested in the study in the first place. Whereas women in the PPI group had all trialled the app at home and work without support from us, many patients from clinic were unable to use their phone beyond calls, texts and photos. Moreover, most of the women we interviewed used the intervention app. We can only speculate as to why this is so but it does mean that concordances and divergences across the intervention and active control arm do need to be treated with circumspection.

For peer review only - http://bmjopen.bmj.com/site/about/guidelines.xhtml

To our knowledge the present observation on failure of PPI work to translate into practice in a trial has not been formally reported before, and is lacking from a recent comprehensive systematic review.(53) PPI involvement is a stipulated requirement when applying for some funding, and the present research findings should be taken into account when drafting guidelines for future PPI involvement in study planning. PPI groups are able to provide significant help and advice in any study but our findings shows the value of adding agile co-development as a requirement for app intervention development as likely to provide a more effective intervention than one informed by PPI alone. Moreover, there is a difference between app use for active clinical management (as with our study participants), and consideration of the potential for app use for this (as with our PPI group).

Implications for clinicians and policymakers

Given the patchy use of the app and the way that some participants did not manage to unlock its full functionality, and an indication of diffusion of benefit, more work is needed to see whether the app reduces pain per se. This study is a good example of the need to move away from 'one size fits all' behavioural interventions. Future studies should do more work on implementation before doing an effectiveness trial. This will enable researchers to be more nuanced about saying who the app is effective for, if at all.

Strategies to involve busy, less motivated, and less technologically experienced individuals in PPI and lay app design groups need to be further developed. These groups should include considerable scaffolding, which we have shown extends to study involvement by patients. More care is also needed to obtain PPI input that is representative of the target group, taking into account their capabilities, opportunities and motivational aspects. It may be useful to give the PPI group a small condition management task that emulates what trial participants will be required to do. Moreover, we can confirm a recent review suggesting

For peer review only - http://bmjopen.bmj.com/site/about/guidelines.xhtml

that health apps should be co- designed with users (7), rather than presenting them with a pre-existing app. These implications for our study are also generalisable to other technology studies.

Author contributions

EB led the study as the CI. EB and CR were the main authors of the grant application for this study, and co-lead authors of the current paper. FR, SJCT, JD, JB, SN and LS contributed to the study design and initial protocol. All authors provided support throughout the trial and contributed towards the final paper. CR led on the PPI and CR and LS led the interview and focus group field work and analysis reported here. CR, LS, SN, CCC, JD and JB were all involved in the field work.

Role of the funding source

The UK National Institute of Health Research, Research for Patient Benefit (RfPB No. PB-PG1013-32025) funded the MEMPHIS study. The funder had no role in the study design, in the collection, analysis, and interpretation of the data, in the writing of this report, or in the decision to submit the paper for publication. The first and last authors vouch for the integrity, completeness and accuracy of the data and analyses, and for the fidelity of this report to the protocol and statistical analysis plan. The views and opinions expressed herein are those of the authors and do not necessarily reflect those of the RfPB, NIHR, NHS or the Department of Health.

Competing interests

We have read and understood BMJ policy on declaration of interests. We have no competing interests to declare.

Data sharing

The data are collected from a small number of people which could compromise their identity if shared with others. Therefore we are not making them available except under exceptional circumstances which will be determined by the custodian of the data (Elizabeth Ball) on an individual basis.

Transparency

For peer review only - http://bmjopen.bmj.com/site/about/guidelines.xhtml

The lead author confirms that the manuscript is an honest, accurate and transparent account of the study being reported; that no important aspects of the study have been omitted; and that any discrepancies from the study as planned and registered have been explained.

Acknowledgements

We would like to thank all the researchers, consultant obstetricians and gynaecologists and data assistants at each of the recruiting clinics for their hard work in promoting the study, recruiting participants and for data entry. Our thanks go to the Trial Steering Committee; Andrew Horne, Sohinee Bhattacharya, Christina Liossi, and Hulya Guzel for their constant support throughout the trial.

We thank the Pelvic Pain Support Network and Endometriosis UK for their promotion and guidance in developing the study design. We would also like to acknowledge the NIHR RfPB programme for their on-going support.

Lastly, thank you to Headspace Ltd for providing our participants with access to the Headspace platform, designing novel content for the study, and for their continuous support and advise throughout the study.

References

1. Ali EE, Chew L, Yap KY. Evolution and current status of mhealth research: a systematic review. BMJ Innovations 2016;2:33-40
2. Ofcom. The Communications Market Report. Ofcom: United Kingdom 2015. https://www.ofcom.org.uk/research-and-data/multi-sector-research/cmr/cmr15/uk [accessed 21 March 2019]
3. Kayyali R. Peletidi A. Ismail M, Hashim Z. Bandeira P, Bonnah J. Awareness and Use of mHealth Apps: A Study from England. Pharmacy 2017, 5, 33.
4. Sezgin E, Yildirim S, Özkan-Yildirim S, Sumuer E. Current and Emerging mHealth Technologies: Adoption, Implementation, and Use: Springer International Publishing; 2018 2018.

For peer review only - http://bmjopen.bmj.com/site/about/guidelines.xhtml

5. Lunden I. 6.1B Smartphone Users Globally By 2020, Overtaking Basic Phone Subscriptions https://techcrunch.com/2015/06/02/6-1b-smartphone-users-globally-by-2020-overtaking-basic-fixed-phone-subscriptions/?guccounter=12015 [cited 2018 28 August 2018].
6. Subhi Y, Bube SH, Rolskov Bojsen S, Skou Thomsen AS, Konge L. Expert Involvement and Adherence to Medical Evidence in Medical Mobile Phone Apps: A Systematic Review. JMIR Mhealth Uhealth. 2015;3(3):e79.
7. Edwards EA, Lumsden J, Rivas C, et al. Gamification for health promotion: systematic review of behaviour change techniques in smartphone apps. BMJ Open. 2016;6(10):e012447. doi:10.1136/bmjopen-2016-012447.
8. de la Vega R, Miro J. mHealth: a strategic field without a solid scientific soul. a systematic review of pain-related apps. PLoS One. 2014;9(7):e101312.
9. Engeler D, Baranowski AP, Borovicka J, et al. European Association of Urology. Guidelines on chronic pelvic pain. http://uroweb.org/wp-content/uploads/EAU-Guidelines-Chronic-Pelvic-Pain-2015.pdf. Accessed Sept 19, 2019.
10. Ahangari, A. Prevalence of chronic pelvic pain among women: an updated review. Pain Physician. 2014 Mar-Apr;17(2):E141-7.
11. Latthe, P., Latthe, M., Say, L., Gülmezoglu, M., & Khan, K. S. (2006). WHO systematic review of prevalence of chronic pelvic pain: a neglected reproductive health morbidity. BMC public health, 6, 177. doi:10.1186/1471-2458-6-177
12. Ayorinde AA, Macfarlane GJ, Saraswat L, Bhattacharya S. Chronic pelvic pain in women: an epidemiological perspective. Womens Health (Lond). 2015;11(6):851-64.
13. Howard F. The Role of Laparoscopy in Chronic Pelvic Pain: Promise and Pitfalls. Obstetrical & Gynecological Survey. 1993;48(6):357-87.
14. Zondervan KT, Yudkin PL, Vessey MP, Jenkinson CP, Dawes MG, Barlow DH, et al. Chronic pelvic pain in the community--symptoms, investigations, and diagnoses. Am J Obstet Gynecol. 2001;184(6):1149-55.

For peer review only - http://bmjopen.bmj.com/site/about/guidelines.xhtml

15. Curtis L. Unit Costs of Health and Social Care 2014 Personal Social Services Research Unit, University of Kent, Canterbury.: University of Kent, Canterbury; 2014 [Available from: https://www.pssru.ac.uk/project-pages/unit-costs/unit-costs-2014/.

16. Simoens S, Dunselman G, Dirksen C, The burden of endometriosis: costs and quality of life of women with endometriosis and treated in referral centres. Hum Reprod. 2012 May;27(5):1292-9. doi: 10.1093/humrep/des073. Epub 2012 Mar 14.

17. Peters AA, van Dorst E, Jellis B, van Zuuren E, Hermans J, Trimbos JB. A randomized clinical trial to compare two different approaches in women with chronic pelvic pain. Obstet Gynecol. 1991;77(5):740-4.

18. Ball EF, Nur Shafina Muhammad Sharizan E, Franklin G, Rogozinska E. Does mindfulness meditation improve chronic pain? A systematic review. Curr Opin Obstet Gynecol. 2017;29(6):359-66.

19. Kold M, Hansen T, Vedsted-Hansen H, Forman A. Mindfulness-based psychological intervention for coping with pain in endometriosis. Nordic Psychology. 2012;64(1):2-16.

20. Fox SD FE, Allen RH. Mindfulness meditation for women with chronic pelvic pain: a pilot study. J Reprod Med 2011;56(3-4):158-62.

21. Bishop SR. Mindfulness: A proposed operational definition. Clinical Psychology: Science and Practice; Autumn 2004; 11, 3; Health Module, pg. 230)

22. Plaza I, Demarzo MM, Herrera-Mercadal P, Garcia-Campayo J. Mindfulness-based mobile applications: literature review and analysis of current features. JMIR Mhealth Uhealth. 2013;1(2):e24.

23. Hilton L, Hempel S, Ewing BA, Apaydin E, Xenakis L, Newberry S, et al. Mindfulness Meditation for Chronic Pain: Systematic Review and Meta-analysis. Ann Behav Med. 2017;51(2):199-213.

24. Wang J,Wang Y, Wei C, Yao NA, Yuan A, Shan Y, Yuan C. Smartphone interventions for long-term health management of chronic diseases: an integrative

For peer review only - http://bmjopen.bmj.com/site/about/guidelines.xhtml

review. Telemed J E Health. 2014 Jun;20(6):570-83. doi: 10.1089/tmj.2013.0243. Epub 2014 May 1.

25. Boudreaux ED, Waring ME, Hayes RB, Sadasivam RS, Mullen S, Pagoto S. Evaluating and selecting mobile health apps: strategies for healthcare providers and healthcare organizations. Transl Behav Med. 2014;4(4):363-71.

26. My Health Apps [Available from: http://myhealthapps.net/app/details/127/Headspace-on-the-go] (Accessed 28 August 2018).

27. Mani M, Kavanagh DJ, Hides L, Stoyanov SR. Review and Evaluation of Mindfulness-Based iPhone Apps. JMIR Mhealth Uhealth. 2015;3(3):e82.

28. App Annie 2018 [Available from: https://www.appannie.com/en/] (Accessed 28 August 2018).

29. Ball E, Newton S, Kahan BC, Forbes G, Wright N, Cantalapiedra Calvete C, et al. Smartphone App Using Mindfulness Meditation for Women With Chronic Pelvic Pain (MEMPHIS): Protocol for a Randomized Feasibility Trial. JMIR Res Protoc. 2018;7(1):e8.

30. Forbes G, Newton S, Cantalapiedra C, Birch J, Dodds J, Steed E, Rivas C, Khan KS, Rohricht F, Taylor SJC, Kahan B, Ball E. A smartphone app using psychological approaches for women with chronic pelvic pain (MEMPHIS): a randomised feasibility trial. submitted

31. Gerhardt-Powals J. Cognitive engineering principles for enhancing human-computer performance. International Journal of Human-Computer Interaction. 1996;8(2):189-211.

32. Murray E, Treweek S, Pope C, MacFarlane A, Ballini L, Dowrick C, et al. Normalisation process theory: a framework for developing, evaluating and implementing complex interventions. BMC Med. 2010;8:63.

33. Borkan J. Immersion/Crystallization. In BF Crabtree and WL Miller (Eds) Doing Qualitative Research (2nd edition). Thousand Oaks, CA: Sage Publication; 1999. p. pp. 179-94.

For peer review only

34. O'Brien BC, Harris IB, Beckman TJ, Reed DA, Cook DA. Standards for reporting qualitative research: a synthesis of recommendations. Acad Med. 2014;89(9):1245-1251.

35. McCann, S. K., Campbell, M. K., & Entwistle, V. A. (2010). Reasons for participating in randomised controlled trials: conditional altruism and considerations for self. Trials, 11, 31. doi:10.1186/1745-6215-11-31

36. Palmer, M., Sutherland, J., Barnard, S., Wynne, A., Rezel, E., Doel, A., Grigsby-Duffy, L., Edwards, S., Russell, S., Hotopf, E., Perel, P. Free, C. (2018). The effectiveness of smoking cessation, physical activity/diet and alcohol reduction interventions delivered by mobile phones for the prevention of non-communicable diseases: A systematic review of randomised controlled trials. PloS one, 13(1), e0189801. doi:10.1371/journal.pone.0189801

37. Marcolino MS, Oliveira JAQ, D'Agostino M, Ribeiro AL, Alkmim MBM, Novillo-Ortiz D. The Impact of mHealth Interventions: Systematic Review of Systematic Reviews. JMIR Mhealth Uhealth. 2018;6(1):e23.

38. Laurie J, Blandford A. Making time for mindfulness. Int J Med Inform. 2016;96:38-50.

39. Collins M, Shattell M, Thomas SP. Problematic Interviewee Behaviors in Qualitative Research. Western Journal of Nursing Research. 2016;27(2):188-99.

40. Michie S, Richardson M, Johnston M, Abraham C, Francis J, Hardeman W, et al. The behavior change technique taxonomy (v1) of 93 hierarchically clustered techniques: building an international consensus for the reporting of behavior change interventions. Ann Behav Med. 2013;46(1):81-95.

41. Mohr DC, Cuijpers P, Lehman K. Supportive accountability: a model for providing human support to enhance adherence to eHealth interventions. J Med Internet Res. 2011;13(1):e30.

42. Andersson G. Using the Internet to provide cognitive behaviour therapy. Behav Res Ther. 2009;47(3):175-80.

For peer review only - http://bmjopen.bmj.com/site/about/guidelines.xhtml

43. Michie S, van Stralen MM, West R. The behaviour change wheel: a new method for characterising and designing behaviour change interventions. Implement Sci. 2011;6:42.

44. Walsh JC, Corbett T, Hogan M, Duggan J, McNamara A. An mHealth Intervention Using a Smartphone App to Increase Walking Behavior in Young Adults: A Pilot Study. JMIR Mhealth Uhealth. 2016;4(3):e109. Published 2016 Sep 22. doi:10.2196/mhealth.5227

45. Geraghty AW, Torres LD, Leykin Y, Perez-Stable EJ, Munoz RF. Understanding attrition from international Internet health interventions: a step towards global eHealth. Health Promot Int. 2013;28(3):442-52.

46. Mistler LA, Ben-Zeev D, Carpenter-Song E, Brunette MF, Friedman MJ. Mobile Mindfulness Intervention on an Acute Psychiatric Unit: Feasibility and Acceptability Study. JMIR Ment Health. 2017;4(3):e34.

47. Noone C, Hogan MJ. A randomised active-controlled trial to examine the effects of an online mindfulness intervention on executive control, critical thinking and key thinking dispositions in a university student sample. BMC Psychol. 2018;6(1):13.

48. Wen L, Sweeney TE, Welton L, Trockel M, Katznelson L. Encouraging Mindfulness in Medical House Staff via Smartphone App: A Pilot Study. Acad Psychiatry. 2017;41(5):646-50.

49. Morrison Wylde C, Mahrer NE, Meyer RML, Gold JI. Mindfulness for Novice Pediatric Nurses: Smartphone Application Versus Traditional Intervention. J Pediatr Nurs. 2017;36:205-12.

50. Lim D, Condon P, DeSteno D. Mindfulness and compassion: an examination of mechanism and scalability. PLoS One. 2015;10(2):e0118221.

51. Arean PA, Hallgren KA, Jordan JT, Gazzaley A, Atkins DC, Heagerty PJ, et al. The Use and Effectiveness of Mobile Apps for Depression: Results From a Fully Remote Clinical Trial. J Med Internet Res. 2016;18(12):e330.

For peer review only

For peer review only - http://bmjopen.bmj.com/site/about/guidelines.xhtml

52. Gilbody S, Lewis H, Adamson J, Atherton K, Bailey D, Birtwistle J, et al. Effect of Collaborative Care vs Usual Care on Depressive Symptoms in Older Adults With Subthreshold Depression: The CASPER Randomized Clinical Trial. JAMA. 2017;317(7):728-37.

53. Brett J, Staniszewska S, Mockford C, Herron-Marx S, Hughes J, Tysall C, et al. A systematic review of the impact of patient and public involvement on service users, researchers and communities. Patient. 2014;7(4):387-95.

For peer review only

For peer review only - http://bmjopen.bmj.com/site/about/guidelines.xhtml

APPENDIX 1

Feature	Positive aspects	Negative aspects	Solutions
**Voice**	Liked by some participants, considered soothing and even spiritual	The voice was not relaxing. One had to listen carefully which meant you could not switch off; otherwise you could not hear instruction clearly during meditation.<br><br>It was also too repetitive, after 10 times it felt like a chore and not something to look forward to.	Choice of different voices<br><br>participants should be told it takes time to get into the rhythm of the instructions, but you can get used to it
**General interface aesthetics**	colours really fresh, interface not too busy, with pleasing layout and aesthetics	one participant would prefer different, bright, colours such as purple	None needed
**Graphics**		The pictures were often not appropriate as they focused on the problem (e.g. tooth pain logo) rather than something positive or soothing. Unanticipated problems could arise according to one participant; she found it hard to lose weight and felt the food icons a) did not represent success and b) reminded her of eating.<br><br>One participant found the pictures hard to see.	Psychological theory supports the view that positive images would be advisable

For peer review only

**Animations**	liked	poorly chosen	None needed
**Progress**	One participant liked the way the app helped progress through the stages.<br><br>Another participant, with cyclical pain and hence cyclical use, did not aim to progress but simply chose to use the baseline video each time.	Three participants were frustrated and even 'angry' and 'patronised' at having to do the same (basic) meditation several times before moving up a stage.<br><br>There was no indication of goals, duration of each level or gamification rewards.	Goal setting is recommended by psychological theory and could be as simple as using "day 1 of 30" rather than simply Day 1.<br><br>Changing emojis could show progress, for example from a sad face to a smiley face to a heart; the inbuilt progress function was not felt sufficiently motivating.
**Introduction**		Experienced app users suggested the introduction could be improved for initiates	overview of the entire app and its levels suggested for the start.<br><br>One participant had done mindfulness before but thought an introduction to mindfulness might be helpful for others.
**Enjoyment**	considered fun by some participants.	One said it was not very 'interesting'.	Include examples of a patient's day with the app to cater for a greater variety of participants<br><br>It would be good to have other features as drop down options that were fun and

For peer review only

			did not involve meditation.

**Session intensity**		Would be good to choose the duration of the meditation.	15 or 20 mins would be better than 10, which is not enough time shut off and meditate deeply.<br><br>The option to control this was important as even 10 minutes was a difficult commitment for some busy participants (1074, 1075, 1078) or those battling with chronic fatigue (1074)
**Pain modules**		it can be hard to focus on something like using the app when you are in pain.	None needed

Comments on app acceptability and usability made by n=13 patients. Two patients walked through the app with the researcher

For peer review only

Reporting checklist for qualitative study.

Based on the SRQR guidelines.

Instructions to authors

Complete this checklist by entering the page numbers from your manuscript where readers will find each of the items listed below.

Your article may not currently address all the items on the checklist. Please modify your text to include the missing information. If you are certain that an item does not apply, please write "n/a" and provide a short explanation.

Upload your completed checklist as an extra file when you submit to a journal.

In your methods section, say that you used the SRQR reporting guidelines, and cite them as:

O'Brien BC, Harris IB, Beckman TJ, Reed DA, Cook DA. Standards for reporting qualitative research: a synthesis of recommendations. Acad Med. 2014;89(9):1245-1251.

		Reporting Item	Page Number
	#1	Concise description of the nature and topic of the study identifying the study as qualitative or indicating the approach (e.g. ethnography, grounded theory) or data collection methods (e.g. interview, focus group) is recommended	6
	#2	Summary of the key elements of the study using the abstract format of the intended publication; typically includes background, purpose, methods, results and conclusions	2
Problem formulation	#3	Description and signifcance of the problem / phenomenon studied: review of relevant theory and empirical work; problem statement	4 and 5
Purpose or research question	#4	Purpose of the study and specific objectives or questions	5
Qualitative approach and research paradigm	#5	Qualitative approach (e.g. ethnography, grounded theory, case study, phenomenolgy, narrative research) and guiding theory if appropriate; identifying the research paradigm (e.g. postpositivist, constructivist / interpretivist) is also recommended; rationale. The	7

For peer review only

		rationale should briefly discuss the justification for choosing that theory, approach, method or technique rather than other options available; the assumptions and limitations implicit in those choices and how those choices influence study conclusions and transferability. As appropriate the rationale for several items might be discussed together.	
Researcher characteristics and reflexivity	#6	Researchers' characteristics that may influence the research, including personal attributes, qualifications / experience, relationship with participants, assumptions and / or presuppositions; potential or actual interaction between researchers' characteristics and the research questions, approach, methods, results and / or transferability	19
Context	#7	Setting / site and salient contextual factors; rationale	7
Sampling strategy	#8	How and why research participants, documents, or events were selected; criteria for deciding when no further sampling was necessary (e.g. sampling saturation); rationale	7
Ethical issues pertaining to human subjects	#9	Documentation of approval by an appropriate ethics review board and participant consent, or explanation for lack thereof; other confidentiality and data security issues	6
Data collection methods	#10	Types of data collected; details of data collection procedures including (as appropriate) start and stop dates of data collection and analysis, iterative process, triangulation of sources / methods, and modification of procedures in response to evolving study findings; rationale	7
Data collection instruments and technologies	#11	Description of instruments (e.g. interview guides, questionnaires) and devices (e.g. audio recorders) used for data collection; if / how the instruments(s) changed over the course of the study	8
Units of study	#12	Number and relevant characteristics of participants, documents, or events included in the study; level of participation (could be reported in results)	See note 1
Data processing	#13	Methods for processing data prior to and during analysis, including transcription, data entry, data management and security, verification of data integrity, data coding, and anonymisation / deidentification of excerpts	8
Data analysis	#14	Process by which inferences, themes, etc. were identified and	8

For peer review only

		developed, including the researchers involved in data analysis; usually references a specific paradigm or approach; rationale	
Techniques to enhance trustworthiness	#15	Techniques to enhance trustworthiness and credibility of data analysis (e.g. member checking, audit trail, triangulation); rationale	8
Syntheses and interpretation	#16	Main findings (e.g. interpretations, inferences, and themes); might include development of a theory or model, or integration with prior research or theory	8-16
Links to empirical data	#17	Evidence (e.g. quotes, field notes, text excerpts, photographs) to substantiate analytic findings	See note 2
Intergration with prior work, implications, transferability and contribution(s) to the field	#18	Short summary of main findings; explanation of how findings and conclusions connect to, support, elaborate on, or challenge conclusions of earlier scholarship; discussion of scope of application / generalizability; identification of unique contributions(s) to scholarship in a discipline or field	16
Limitations	#19	Trustworthiness and limitations of findings	19
Conflicts of interest	#20	Potential sources of influence of perceived influence on study conduct and conclusions; how these were managed	4
Funding	#21	Sources of funding and other support; role of funders in data collection, interpretation and reporting	3

Author notes

1. 7,8,9 and especially 10

2. 8-16 and appendix

The SRQR checklist is distributed with permission of Wolters Kluwer © 2014 by the Association of American Medical Colleges. This checklist was completed on 26. March 2019 using https://www.goodreports.org/, a tool made by the EQUATOR Network in collaboration with Penelope.ai

For peer review only
